# Comparative mitogenomics and phylogenetics of the family Carangidae with special emphasis on the mitogenome of the Indian Scad *Decapterus russelli*

**DOI:** 10.1038/s41598-022-09636-5

**Published:** 2022-04-04

**Authors:** Anjaly Jose, Sandhya Sukumaran, Lakshmi P. Mukundan, Neenu Raj, Sujitha Mary, K. Nisha, A. Gopalakrishnan

**Affiliations:** 1 Marine Biotechnology Division, ICAR-Central Marine Fisheries Research Institute, Ernakulam North P O, Kochi, Kerala 682018 India; 2 Mangalore University, Mangalagangotri, Mangalore, Karnataka 574199 India

**Keywords:** Genetics, Evolutionary biology, Genome, Sequencing

## Abstract

Carangids are abundant and commercially important marine fish that contribute to a significant portion of the fisheries in many parts of the world. In the present study, we characterized the complete mitogenome of the Indian scad, *Decapterus russelli* and performed a comprehensive comparative mitogenomic analysis of the family Carangidae. The comparative mitogenomics provided valuable insights into the structure, variability, and features of the coding and non-coding regions that evolved across species over millions of years. The structural features of tRNAs revealed changes in the frequency of mismatched and wobble base pairs, which is reflected in the base composition of H and L strands. The highly conserved sequence motif of the mTERF binding site in carangids over the ~ 400 MYA of their divergence demonstrated the functional importance of these sites. The control region of carangids was characterized by the presence of discontinuous repeat units with a high rate of sequence divergence in the form of base substitutions, insertions, and deletions. The maintenance of secondary structures in the control region independent of the rapid evolution of primary structure suggested the effect of selective constraints on their maintenance. Maximum likelihood (ML) and Bayesian inference (BI) phylogeny revealed a similar topology consistent with previous taxonomic studies. The extant carangids diverged through the evolutionary events experienced during the Cretaceous, Paleogene, and Neogene periods.

## Introduction

The carangids represent an important group of exploited pelagic coastal resources of the world with diverse morphological features comprising approximately 147 species^1^. They occupy a variety of habitats consisting of pelagic, benthopelagic and reef-associated regions in all tropical and subtropical seas^2,3^. Currently, 4 subfamilies are recognized within the Carangidae^4^. Annual global capture production of Carangidae was estimated at 5.13 million tons in 2018, 65% of which came from Asia (https://www.fao.org/fishery/en/statistics). Among the carangids, one of the commercially most important species is the Indian scad, *D. russelli* (Ruppell, 1830), providing a cheap source of animal protein. It is also commonly used as live fish bait^5^. It is widespread in the Indo-Pacific and forms an important fishery resource in India and Southeast Asia^6^. Most studies on carangids have focused on biology, ecology, and taxonomy^7–18^, and it is important to study the genetic and genomic traits to resolve taxonomic ambiguities, understand evolutionary traits, and implement conservation strategies.

The vertebrate mitochondrial genome, which plays a crucial role in cell metabolism and organism function, is ~ 15–20 kb in size and consists of 13 protein-coding genes (PCGs), 22 tRNA genes, 2 rRNA genes and a non-coding region (control region)^19^. The PCGs, tRNA and rRNA have a relatively low mutation rate because they are subject to strong purifying selection^20^. In contrast, the control region evolves at a high rate compared to the other parts of the mitogenome, and these high rates of sequence divergence are mostly localized in the variable flanking region, which is adjacent to the conserved sequence motifs^21–23^.The extensive comparative studies in fish^24^ have demonstrated the ability of the control region to form stable secondary structures and the existence of selective constraints for their maintenance. Although Zhu et al.^25^ analyzed the phylogenetic relationships of carangids using the control region, there is a gap in knowledge regarding the mutational pattern and the non-random distribution of these mutations between the conserved motifs and their flanking regions. There is also a lack of knowledge about the structure and variability of tRNA genes in carangids and the frequency of occurrence of G-U wobble base pairs, the fundamental building block of RNA structure critical for RNA function in various biological systems.

The phylogenetic relatedness between the carangids has been extensively studied using comparative morphological^4,26–31^ and molecular tools^32–37^. Although there has been a discourse on monophyly^4,28–31^ vs. paraphyly^36,37^ of carangids, the congruent topologies of phylogenetic trees constructed using DNA sequences suggested paraphyly of carangids if Echeneoids were not included^35–38^. Carangids can be considered as promising candidates for evolutionary studies, as they have a wide diversity in terms of habitat (marine, brackish water, freshwater), morphology (having variable body size; less than 20 cm to ~ 2 m), and shape (deep-bodied, streamlined). Some species of remoras live even as commensals on large pelagic vertebrates^39^. The phylogeographic study inferred from nuclear gene supermatrix suggested a terminal cretaceous crown age for carangids with major lineages diversifying during the Paleogene^36^. A late Eocene age has also been proposed for the crown carangids^35^. But densely sampled molecular studies indicated a Cenomanian stem age of the Carangoids which had undergone a significant diversification during the late cretaceous^37,38^. The subfamily Naucratinae originated early in the Paleocene^40^, Trachinotinae date back to the early Oligocene and the remaining Carangoid lineages including the morphologically heterogeneous group, Caranginae comprising the genus *Decapterus* appear to have originated during the middle Eocene^38^. The genus *Decapterus* originated in the upper Miocene^41^. The genetic distance between the genus *Decapterus* was significantly greater than that between *Trachurus*^42^, suggesting that *Decapterus* arose long before its sister group *Trachurus*, making them the most divergent species group. The morphology of the genus *Decapterus* includes multiple forms such as long-bodied, cylindrical, mackerel-like with a small mouth, reduced teeth and reduced scutes on the straight part of the lateral line, suggesting adaptations to the wide ranging off-shore life^43^.

In the present study, we report the complete mitogenome of *D. russelli* for the first time elucidating the special characteristics. Although recent studies have examined the evolutionary history of carangids^44^, we reanalyzed the patterns by including mitogenomes from 37 species and describing the relationships and evolutionary patterns among species with a robust phylogenetic tree. We also performed a comprehensive comparative analysis of carangid mitogenomes especially the mutational patterns of coding and non-coding regions, control region secondary structure, and structural features of tRNA to gain insights into the evolutionary dynamics of mitochondrial DNA.

## Results and discussion

### Mitochondrial genome organization and composition

The total length of the mitochondrial genome of *D. russelli* was 16,542 bp. The genome organization is congruent with that observed in other teleost fishes with a standard set of 13 putative PCGs, 22 tRNA genes, 2 rRNA genes, and a control region. All genes were encoded on the heavy strand (H) except for the ND6 and 8 tRNA genes which were encoded on the light strand (L) (Supplementary Fig. S1). A similar gene arrangement was observed in other Carangidae species studied (Supplementary Table S1). The overall nucleotide composition of *D. russelli* mitochondrial genome was 27.5% A, 30.2% C, 25.4% T, and 16.9% G. The AT and GC skew values of the whole mitogenome were 0.039 and − 0.028 respectively.

The results suggested that A and C are predominant base constituents than T and G, with the overall nucleotide composition being severely C-skewed. These results agree well with their respective counterparts in other *Decapterus* spp.^45–47^ The bias against G was presented following the results reported previously in other teleosts^48,49^. The AT-/GC- skew values of other Carangidae species were also determined (Supplementary Table S2). The sequence of the complete mitogenome of *D. russelli* has been deposited with NCBI, GenBank with the accession number MN711693.

### Overlapping and Intergenic spacer regions

Seven gene overlapping regions ranging from 1–7 bp and 11 spacer regions varying in length from 1 to 38 bp were detected in the *D. russelli* mitogenome with the longest intergenic spacer region observed between tRNA Asn and tRNA Cys. Significant differences in the number and length of both the intergenic spacer region and the overlapping gene regions were observed in carangids (Supplementary Table S3). The number of spacer and overlapping gene regions varied between 10–12 and 6–15, respectively, between species. The intergenic spacer is distributed over 11 regions in almost all carangid species studied so far. However, an additional spacer region was observed in *C. armatus* (tRNA-His-tRNA-Ser (GCT)), *E. bipinnulata* (*ND6*-tRNA-Glu), *G. speciosus* (tRNA-Val-16SrRNA), *P. niger* (16SrRNA-tRNA-Leu (TAA)), *S. crumenophthalmus* (*ND6*-tRNA Glu) and *S. dumerili* (*ND6*-tRNA Glu). In addition, the spacer region found between tRNA-Arg and ND4L in all species was absent in *Seriola* spp, *S. nigrofasciata*, *T. carolinus* and *T. ovatus*. Besides, *C. equula* and *P. dentex* lacked a spacer region found between *ND1* and tRNA-Ile which was present in all other taxa. A comparative analysis among 37 carangids revealed that the longest spacer (42 bp) occurred between tRNA-Asn and tRNA-Cys of *T. carolinus*. Seven regions of overlapping gene regions were observed in most carangids, with the longest overlapping region being between ATP8 and ATP6 in some species and between ND4L and ND4 in others.

### Protein Coding Genes (PCGs)

The total length of PCGs in the *D. russelli* mitogenome was 11,425 bp, ranging in size from 165 bp (*ATP8*) to 1839 bp (*ND5*) accounting for 69% of the entire genome. GC skew values of all PCGs were negative except for the case with the L-strand encoded gene, ND6, which was greater than zero (Table 1). Similarly, the majority of PCGs had negative AT skew values. These results suggested that the bases C and T are over-represented in most PCGs. The base skew values for PCGs of the other 36 carangid species are presented in Supplementary Table S2. The GC and AT skew values of all were negative, except the AT skew value for *M. cordyla* and *C. tille* which was positive. Besides, *T. ovatus* was presented with equal numbers of A and T in PCGs, a trend not observed in other carangids.

**Table 1:** Sequence characteristics of *Decapterus russelli* mitochondrial genome.

Locus	Strand	Position	Size (bp)	Codon		A%	T%	G%	C%	A + T	G + C	AT skew	GC skew	Anticodon	Intergenic nucleotide
				Start	Stop										
tRNAPhe	H	1–68	68			35.3	19.1	23.5	22.1	54.4	45.6	0.297	0.030	GAA	
12SrRNA	H	69–1022	954			30.8	21.4	22	25.8	52.2	47.8	0.180	− 0.079		
tRNA Val	H	1023–1094	72			30.6	19.4	23.6	26.4	50	50	0.224	− 0.056	TAC	
16SrRNA	H	1095–2817	1723			32.4	22	20.1	25.4	54.4	45.5	0.191	− 0.106		
tRNALeu (TAA)	H	2818–2891	74			28.4	23	23	25.7	51.4	48.7	0.105	− 0.060	TAA	
ND1	*H*	2892–3866	975	ATG	TAA	23.4	26.4	16	34.3	49.8	50.3	− 0.060	− 0.363		5
tRNA Ile	H	3872–3941	70			27.1	21.4	28.6	22.9	48.5	51.5	0.117	0.110	GAT	
tRNAGln	L	3941–4011	71			35.2	25.4	15.5	23.9	60.6	39.9	0.161	− 0.210	TTG	− 1
tRNA Met	H	4011–4080	70			25.7	25.7	22.9	25.7	51.4	48.6	0.00	− 0.057	CAT	
ND2	H	4081–5125	1045	ATG	T–	27.2	24.1	12.2	36.5	51.3	48.7	0.060	− 0.500		
tRNATrp	H	5126–5196	71			32.4	16.9	25.4	25.4	49.3	50.8	0.315	0.00	TCA	1
tRNAAla	L	5198–5266	69			34.8	24.6	14.5	26.1	59.4	40.6	0.171	− 0.285	TGC	1
tRNAAsn	L	5268–5340	73			32.9	20.5	17.8	28.8	53.4	46.6	0.232	− 0.236	GTT	38
tRNACys	L	5379–5445	67			26.9	26.9	22.4	23.9	53.8	46.3	0.00	− 0.032	GCA	
tRNA Tyr	L	5446–5515	70			30	22.9	18.6	28.6	52.9	47.2	0.134	− 0.211	GTA	1
COI	H	5517–7067	1551	GTG	TAA	24.5	29.7	18.6	27.2	54..2	45.8	− 0.095	− 0.187		
tRNASer (TGA)	L	7068–7138	71			28.2	23.9	19.7	28.2	52.1	47.9	0.082	− 0.180	TGA	3
tRNA Asp	H	7142–7212	71			29.6	28.2	21.1	21.2	53.8	46.3	0.026	− 0.002	GTC	7
COII	H	7220–7910	691	ATG	T–	27.9	25.3	16.6	30.1	53.2	46.7	0.049	− 0.289		
tRNA Lys	H	7911–7984	74			31.1	21.6	20.3	27	52.7	47.3	− 3.614	− 0.141	TTT	1
ATP8	H	7986–8150	165	ATG	TAG	24.1	26.8	13.9	35.2	50.9	49.1	− 0.053	− 0.430		− 7
ATP6	H	8144–8827	684	ATG	TAA	24.1	26.8	13.9	35.2	50.9	49.1	− 0.053	− 0.430		− 1
COIII	H	8827–9611	785	ATG	TA-	23.9	26.6	17.8	31.6	50.5	49.4	− 0.053	− 0.280		
tRNAGly	H	9612–9681	70			37.1	28.6	15.7	18.6	65.7	34.3	0.129	− 0.084	TCC	
ND3	H	9682–10,030	349	ATG	T–	22.1	29.2	15.5	33.2	51.3	48.7	− 0.138	− 0.363		
tRNAArg	H	10,031–10,099	69			31.9	33.3	18.8	15.9	65.2	34.7	− 0.021	0.083	TCG	1
ND4L	H	10,101–10,397	297	ATG	TAA	22.2	27.9	15.5	34.3	50.1	49.8	− 0.113	− 0.380		
ND4	H	10,391–11,771	1381	ATG	T–	25.6	25.8	14.7	34	51.4	48.7	− 0.003	− 0.396		
tRNA His	H	11,772–11,843	72			29.2	29.2	19.4	22.2	58.4	41.6	0.00	− 0.067	GTG	
tRNASer(GCT)	H	11,844–11,911	68			25	17.6	25	32.4	42.6	57.4	0.173	− 0.128	GCT	
tRNALeu(TAG)	H	11,917–11,989	73			31.5	23.3	20.5	24.7	54.8	45.2	0.149	− 0.0930	TAG	
ND5	H	11,990–13,828	1839	ATG	TAG	26.6	26.4	14.2	32.7	53	46.9	0.003	− 0.394		− 4
ND6	L	13,825–14,346	522	ATG	TAG	13.8	36	33.3	16.9	49.8	50.2	− 0.445	0.327		
tRNAGlu	L	14,347–14,415	69			33.3	21.7	17.4	27.5	55	44.9	0.210	− 0.300	TCC	4
Cytb	H	14,420–15,560	1141	ATG	T–	23.7	27.1	15.8	33.5	50.8	49.3	− 0.070	− 0.352		
tRNAThr	H	15,561–15,632	72			22.2	23.6	26.4	27.8	45.8	54.2	− 0.030	− 0.025	TGT	− 1
tRNA Pro	L	15,632–15,702	71			33.8	25.4	12.7	28.2	59.2	40.9	0.134	− 0.378	TGG	
Dloop	H	15,703–16,542	840			33	30.6	16.3	20.1	63.6	36.4	0.037	− 0.104		

The initiation and termination codons in *D. russelli* were confirmed by aligning the corresponding genes to other carangids. Among the 13 PCGs, 12 of them shared the same orthodox initiation codon, ATG, whereas *COI* had a different initiation codon and started with GTG instead of ATG (Table 1). Besides, 6 genes exhibited incomplete termination codons, either TA (*COIII*) or T (*ND2*, *COII*, *ND3*, *ND4*, and *Cyt-b*). The remaining genes revealed complete stop codons in open reading frames either TAA (*ND1*, *COI*, *ATP6*, and *ND4L*) or TAG (*ATP8*, *ND5*, and *ND6*). The incomplete codons can be completed by polyadenylation of the messenger RNA after cleavage^50,51^. Comparative analysis among carangids revealed that most PCGs started with the ATN initiation codon (Supplementary Table S4). *COI* of all the species started with GTG except ATC in *G. speciosus*. All other PCGs start with ATG except ATA for *ATP6* (*A. djedaba*, *A. kleinii*, *A. mate*, *C. ignobilis*, *C. melampygus*, *C. equula*, *C. tille and M. cordyla)* and *ND4L* (*T. trachurus*), and GTG for *ND5* (*T. blochii*, *T. carolinus* and *T. ovatus*).

We observed that the value of Ka was significantly smaller than Ks in all 13 PCGs on analyzing the evolutionary rate of PCGs. This indicates that there is a much lesser chance for a mutation to be different between species that can modify the amino acid sequences of a protein sufficiently to change its function than one which is silent^52^. The average Ka/Ks ratios substantially differed among PCGs indicating varying functional constraints experienced by individual genes (Supplementary Fig. S2; Supplementary Table S5). The fundamental features of genome evolution were largely determined by the influence of two non-adaptive forces, mutation pressure and genetic drift^53^. Nevertheless, the deleterious effect of mutation is very difficult to establish under purifying selection^54^. In PCGs, the average Ka/Ks ratio varied from 0.008 in *COI* to 0.469 in *ATP8*. Altogether the results indicated that the functional genes of carangids have undergone strong purifying selection to eliminate deleterious mutations thus maintaining the protein structure.

We estimated the degree of mitochondrial gene conservation by assessing the total p-genetic distance between 37 Carangidae species (Supplementary Fig. S3). In our analysis, we obtained the highest overall p genetic distance for the *ATP8* gene (0.301) and the lowest value for the *COI* gene (0.019) based on the data of the first and second nucleotide position of codons. The same trend was also observed in the values discerned from the full-length comparison of each gene, with *ATP8* and *COI* represented by a total p-genetic distance value of 0.390 and 0.134, respectively. Based on these results it could be inferred that gene, *ATP8* has the fastest evolutionary rate among Carangidae and *COI* the slowest. Besides, all genes had overall high p-genetic distance values for wobble nucleotide position, ranging from 0.393 to 0.564. This result was consistent with previous reports in other fish species that most of the differences occurred at the 3^rd^ codon position of PCGs in mtDNA^55^.

### Codon usage analysis

A total of 3800 amino acids of PCGs are encoded in the mitogenome of *D. russelli*. The codon usage patterns are shown in Supplementary Fig. S4A. The amino acid Leu was represented by six different codons and the rest of them by either four or two. The amino acids Leu (16.46), Ala (8.75), and Thr (7.96) were most abundant in the *D. russelli* mitogenome. Among these, the frequency of Leu (CUC, CUA), Ala (GCC), and Thr (ACC) was highest. The frequencies of stop (UAG), Cys (UGU), and Arg (CGG) in PCGs were lowest. A comparative analysis showed that the codons ending in A or C occurred most frequently in amino acids with sixfold and fourfold degenerate third positions in Carangidae. In a two-fold degenerate codon, C was used more frequently than T. Consistent with the overall bias against G^56^, it was the least common wobble position nucleotide in all categories. However, analysis of relative synonymous codon usage (RSCU) showed that the codons UCC for Ser, CUC and CUA for Leu and GCC for Ala occurred most frequently, while UUG for Leu, AGU for Ser and GCG for Ala were rare in the mitogenome of Carangidae (Supplementary Fig. S4B). It was also inferred from the RSCU value that the synonymous codons NNA and NNC were in the majority than the codons NNT and NNG (Supplementary Table S6).

### Transfer RNAs and ribosomal RNAs

The mitogenome of *D. russelli* contained 22 tRNA genes typical of the metazoan mitogenome. In total, the tRNA genes contributed 1555 bp to the entire genome, which ranged from 67 bp (tRNA-Cys) to 74 bp (tRNA-Lys (TAA)). The secondary structure of tRNA genes predicted by ARWEN^57^ makes it clear that all tRNAs could be folded into a canonical cloverleaf structure, with the exception of tRNA-Ser (GCT), in which no recognizable dihydrouridine (DHU) stem was found (Supplementary Fig. S5). Comparative analysis with other carangids revealed that the total length of tRNAs varied from 1548 (*S. rivoliana* and *E. bipinnulata)* to 1564 (*T. blochii*). The overall A + T content was estimated at 54.2%. Base skew analysis of 22 concatenated tRNA genes (Supplementary Table S2 revealed a positive AT skew (0.125) and a negative GC skew (− 0.098). The comparisons showed that the AT skew varied from 0.105 (*S. nigrofasciata*) to 0.224 (*T. ovatus*) and the GC skew varied from -0.120 (*S. rivoliana*) to − 0.091 (*T. carolinus*). The anticodons of these 22 tRNA genes were identical to those reported in other carangids (Supplementary Table S7). Despite the general one-to-one codon-anticodon correspondence, tRNA genes for Ser (TGA and GCT) and Leu (TAA and TAG) have been determined by two types of anticodons. Furthermore, G-U mismatches were observed for 16 of the tRNAs in their respective secondary structures (Supplementary Fig. S5). Five of the tRNA genes appeared to have A-C mismatches (tRNA-Val (TAC), tRNA-Leu (TAA), tRNA-Ile (GAT), tRNA-Arg (TCG), tRNA-His (GTG) in their respective amino acid acceptor stems. Similarly, two A-A mismatches, each for tRNA-Phe (GAA) and tRNA-Ser (GCT) and two C–C mismatches each for tRNA-Leu (TAG) and tRNA-Thr (TGT) were also detected. The presence of unmatched base pairs in mitochondrial tRNAs appeared to be a general trend that can later be repaired by post-transcriptional editing mechanisms prevalent in vertebrate mtDNA^58^.

Two rRNA genes, a small (12 s rRNA) and a large (16 s RNA) subunit were located in the H strand of *D. russelli*. The total length of two concatenated rRNA genes was estimated to be 2677 bp with an A + T content of 53.7% (Supplementary Table S2). The AT content of the 12S rRNA gene was 52.2% with a positive AT skew of 0.172 and a negative GC skew of  − 0.079. Similarly, the AT content of the 16S rRNA gene was 54.4% with a positive AT skew (0.191) and a negative GC skew (− 0.116). Moreover, the presence of a higher proportion of A and C bases in rRNA genes indicates an AC-rich trend, consistent with previous reports from other teleosts^53,59,60^. Consistent with previously reported results from other mitochondrial genomes^61–63^, these two rRNA genes were positioned between tRNA-Phe and tRNA- Leu (TAA) separated by tRNA-Val. Comparative analysis among carangids (Supplementary Fig. S6) indicated that the length of two concatenated rRNA genes varied from 2655 bp (*G. speciosus*) to 2683 bp (*A. kleinii*). The AT skew varied from 0.1615 to 0.2256 and the GC skew varied from − 0.124 to − 0.077 (Supplementary Table S2).

### Features of tRNAs

#### Secondary structure

**Figure 1:**
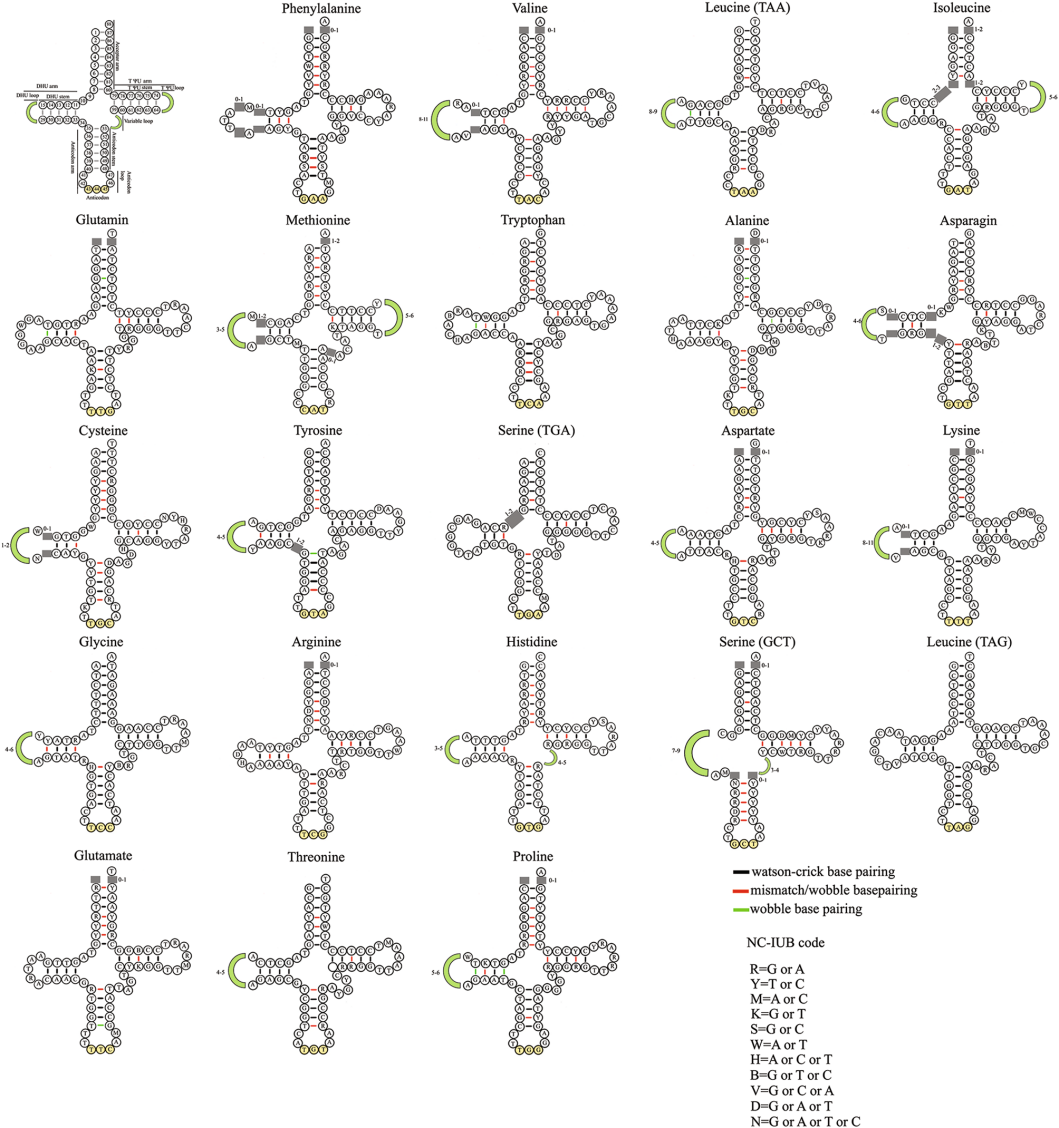
Secondary structure of tRNAs showing structural variations in 37 species. The first structure represents the nucleotide positions common to all tRNAs (1–88) and details of stem- loop. Length variations in stems and loops are indicated by grey squares and green arcs respectively. The secondary structure of tRNAs were predicted by ARWEN software (http://130.235.244.92/ARWEN/) and edited manually in Adobe Photoshop CS6.0.

The comparative analysis of 37 carangids revealed that most of the tRNAs are structurally different in both the stem and the loop. However, 2 tRNA genes, tRNA-Trp, and tRNA-Leu (TAG) exhibited similarities in their secondary structure (Fig. 1). As can be seen from Table 2, length variation was detected in all four stems. The anticodon stem (AC stem) of all the tRNAs was 5 bp, except for tRNA-Ser (GCT), which displayed length variation (5 or 6 bp). Though the pseudouridine (TΨU) stem of most tRNAs was 5 bp, tRNA-Phe (4 bp), tRNA-Met (4 or 5 bp), tRNA-Lys (4 bp), and tRNA-Ser (GCT) (6 bp) indicated length variation. In the acceptor stem (AA stem), tRNA-Ser (TGA) displayed a length of 6 bp, and the rest of the tRNAs showed more of a length variation of either 7 bp or a range of 7 to 8 bp. Although the DHU stem was mostly 4 bp in length with a range of 3 to 5 bp, tRNA-Ser (GCT) of all carangids examined had only a small loop with no stem.

**Table 2:** Size of stems and loops of 22 tRNA genes in the mitogenomes of 37 species of Carangidae.

tRNA	AA stem (bp)			DHU stem (bp)				DHU loop (nucleotide)											AC stem (bp)		V loop (nucleotide)			TΨU stem (bp)			TΨU loop (nucleotide)			
	6	7	8	0	3	4	5	3	4	5	6	7	8	9	10	11	12	13	5	6	3	4	5	4	5	6	6	7	8	9
**H strand coded**																														
Phe		35	2		12	25				24		13							37			37		37					37	
Val		35	2		33	4							4		33				37			37			37			37		
Leu(TAA)		37				37									21	16			37			37			37			37		
Ile		18	19		37						1	36							37				37		37			26	11	
Met		2	35		8	29				7	18	12							37			34	3	1	36			36	1	
Trp		37				37						36	1						37			37			37			37		
Asp		36	1			37					3	34							37			37			37			37		
Lys		33	4		36	1									1	6	29	1	37				37	37						37
Gly		37				37					28	6	3						37			37			37			37		
Arg		31	6			37				37									37			37			37			37		
His		37				37				3	2	32							37			11	26		37			37		
Ser(GCT)		3	34	34												6	30	1	7	30	31	6				37		37		
Leu(TAG)		37				37							37						37				37		37			37		
Thr		37				37					3	32	2						37				37		37			37		
**L strand coded**																														
Gln		32	5			37						37							37			37			37			37		
Ala		20	17			37				37									37			37			37			37		
Asn		37				27	10				14		23						37				37		37			37		
Cys			37		5	32		23	14										37			37			37		2	35		
Tyr		37				37					34	3							37			37			37			37		
Ser(TGA)	37				37									37					37			37			37			37		
Glu		32	5			37				37									37			37			37			37		
Pro		32	5			37						36	1						37			34			37			37		

Of the four loops, the length of the AC loop was fixed (4 nucleotides), whereas the remaining three loops showed considerable variation in length. Of these, the DHU loop was extremely variable, ranging from 3–13 nucleotides. Conversely, the variable loop (V loop), mostly 4 nucleotides with a range of 3–5, and T ΨU loop, mostly 7 nucleotides with a range of 6–9 displayed the least variation.

#### Wobble base pairs in the stem regions

The wobble base pair is a unique feature of RNA structure and their often high evolutionary conservation^64^ underscores their functional and structural importance. Due to its thermodynamic stability, the G-U often substitutes G-C or A-U base pairs and it differs from Watson–Crick and other mismatched base pairs owing to its structural, conformational, and functional properties^65^. Diverse proteins and other ligands specifically targeting tRNA exploit these features^66^ and thus the comparative analysis of tRNAs is very crucial for understanding the structural and functional features of the mitogenome. Supplementary Table S8 illustrates the frequencies of Watson–Crick and Wobble pairing at each of the 25 base pair sites of each tRNA in 37 carangids (8 in AA stem + 5 in DHU stem + 6 in AC stem + 6 in TΨU stem). Seven sites from each stem (1–87, 2–86 and 8–80 base pair sites in the AA stem, 12–32 and 15–30 in the DHU stem, 40–48 in the AC stem, and 64–74 in the TΨU stem) revealed a high rate of (100%) Watson–Crick and Wobble base pairing (hydrogen bonding) and these sites may play a pivotal role in maintaining stem structure. Among the tRNAs, tRNA-Ala, tRNA-Asn, tRNA-Cys, tRNA-Ser (TGA), tRNA-Asp, and tRNA-Pro had a higher frequency (≥ 98% overall average) of hydrogen bonding than other tRNAs. Moreover, L strand-encoding tRNAs exhibited, on average, a higher frequency of hydrogen bonding (97.48%) than H strand encoding tRNAs (94.78%).

Table 3 shows the number of G-U wobble base pairs in each stem of 22 tRNAs for the 37 carangid species. Individual stems and tRNAs rather displayed considerable variations in the frequency of GU base pairs, 6% of the base pairs comprised of wobble base pairs on average in the four stems of mitochondrial tRNA with a large difference between stems (4.57% in the TΨU stem and 12.26% in the DHU stem). A substantial difference was also discerned between the tRNAs (0.72% in tRNA-Ile versus 19% in tRNA-Glu) with no wobble base pairs observed in the case of tRNA-Lys and tRNA-Leu (TAG). Furthermore, the tRNAs encoded by different strands exhibited a significant difference in the frequency of wobble base pairs, as the L- strand encoded tRNAs had a higher frequency of wobble base pairs (13.18%) than those of H- strand encoded tRNAs (3%) (Mann–Whitney U test, *p* < 0.01).

**Table 3:** Occurrence of wobble pairings in the stem regions of 22 tRNA genes in the mitogenome of 37 species of Carangidae.

	tRNA	AA stem	DHU stem	AC stem	TΨU stem	Total	Percentage
	Total base pairs	5871	3066	4066	3999	17,002	
		Wobble bp	Wobble bp	Wobble bp	Wobble bp		
H strand coded	Phe	10	6	0	1	17	2.5
	Val	2	2	0	3	7	0.93
	Leu(TAA)	0	37	0	3	40	4.6
	Ile	6	0	0	0	6	0.72
	Met	33	0	0	11	44	5.50
	Trp	2	35	0	0	37	4.80
	Asp	*2*	0	0	17	19	2.50
	Lys	0	0	0	0	0	0
	Gly	0	65	0	0	65	8.40
	Arg	8	6	2	18	34	4.34
	His	2	7	0	23	32	4.20
	Ser(GCT)	0		5	7	12	1.64
	Leu(TAG)	0	0	0	0	0	0
	Thr	0	0	6	0	6	0.80
	Average (%)	1.72	8.27	0.50	3.29	3.00	
L strand coded	Gln	37	37	0	0	74	9.46
	Ala	42	30	41	34	150	18.82
	Asn	24	37	0	18	79	10.03
	Cys	52	34	1	1	88	10.81
	Tyr	37	0	37	0	74	9.52
	Ser(TGA)	3	6	29	34	66	9.40
	Glu	68	0	71	9	148	19.00
	Pro	35	74	32	1	142	18.13
	Average	14.13	18.84	14.25	6.75	13.18	
	Coefficient of varitaion	1.30	1.29	1.84	1.32	0.88	
	Grand average	6.18	12.26	5.50	4.57	6.70	

#### Mitochondrial transcription termination factor (mTERF) binding site

We compared tRNA-Leu (TAA) which has been reported to contain the target binding site for mTERF in humans^67^ to identify its presence in Carangidae. An important tridecameric sequence (5'TGGCAGAGCCCGG3') reported in the human mitochondrial tRNA-Leu (TAA) gene^48,49^ was also identified in Carangidae in the same region as the corresponding part of their DHU arm. The mTERF binds to this site with high affinity and regulates the level of transcription from the rRNA genes into the remaining genes of the major coding strand^50^.

All bases in the 13 nucleotide positions identified in 37 carangid taxa were completely identical to those in humans (Supplementary Fig. S7), suggesting that this site in carangids may follow the same mechanism of function as proposed for humans.

#### Control region: mutational patterns and secondary structure

The control region of *D. russelli* was shorter than other carangids, consisting of 840 bp flanked by tRNA-Pro and tRNA-Phe on either side. The control region comprised the highest A + T content (63.6%) compared to other regions of the mitogenome (Supplementary Table S2). AT skew was positive (0.038) and GC skew was negative (− 0.104). Considerable variation was observed in the AT and GC skew values of other carangids, ranging from − 0.047 (*S. crumenophthalmus*) to 0.065 (*T. blochii*) and − 0.278 (*C. ignobilis*) to − 0.093 (*D. maruadsi*), respectively. The composition of the nucleotide adenine (A) is higher than that of thymine (T) in *D. russelli* compared to other carangids (Supplementary Table S2). However, the control region of *A. mate*, *A. djedaba, C. ignobilis, G. speciosus,* and *T. blochii* possess a slightly higher percentage of adenine (A) than that of *D. russelli*. Besides, the composition of thymine (T) is higher than adenine (A) in *C. armatus, C. malabaricus, C. tille, C. melampygus, T. japonicas, S. crumenophthalmus, S. dumerili,* and *S. lalandi*. The GC skew of all the carangids was negative. It was found that the maximum length variation for the control region was observed in the range of 840 bp (*D. russelli*) to 904 bp (*S. crumenophthalmus*) in the carangids examined. Among the carangids, *S. crumenophthalmus* possessed the longest mitogenome, and *S. rivoliana* and *S. dumerili* were determined to have the shortest mitogenome. It was evident that these species do not exhibit any notable variation with respect to their PCGs, tRNA and rRNA (Supplementary Fig. S6), hence the 61–62 bp variation in the control region alone contributed to the difference in their overall mitogenome length. The majority of the primary sequences of the control region are presumed to be not involved in regulatory function as this region shows great variation even among closely related species^68^.

**Figure 2:**
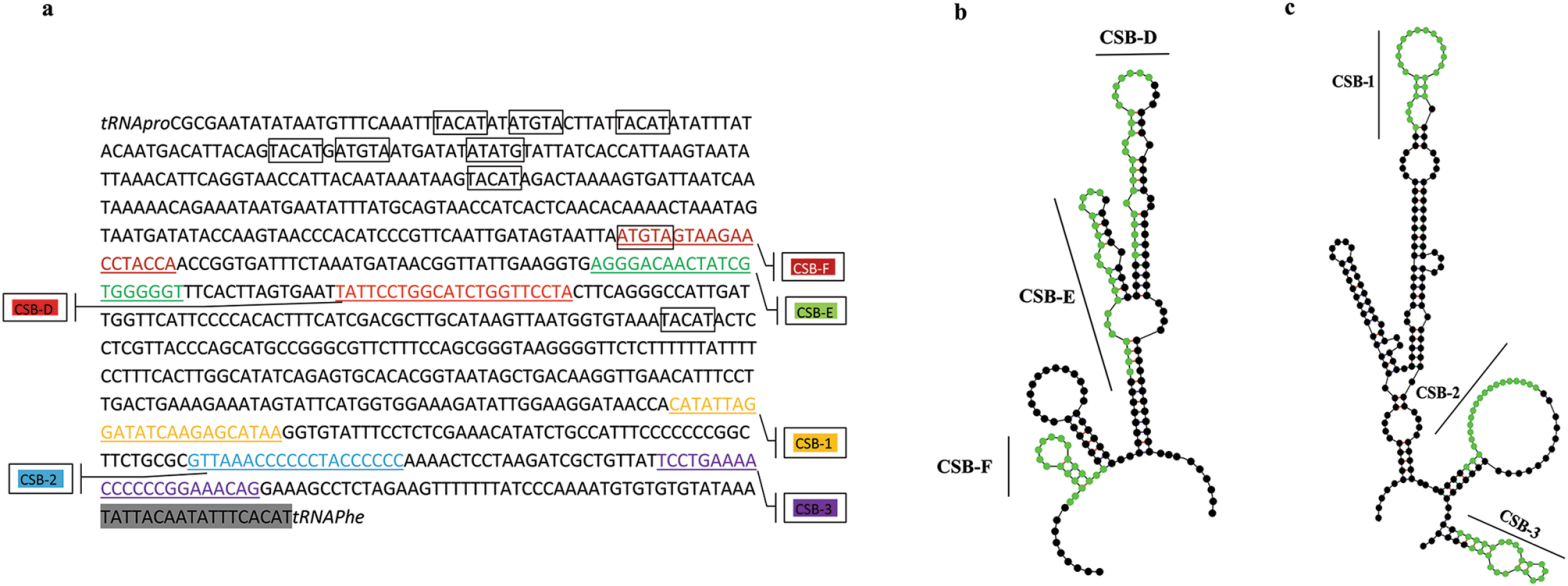
Sequence and structure of the mitochondrial DNA control region of carangids (**a**) Features of the control region. CSB-F,-D,-E: central conserved sequence blocks; CSB-1,-2,-3: conserved sequence blocks. Palindromic sequence motifs 'TACTA' and 'ATGTA' are framed. Grey colored bar represents the characteristic 3' terminal sequence. (**b**) The secondary structure identified in CSB-F, CSB-D, and CSB -E. (**c**) Secondary structure identified in CSB-1, CSB-2, and CSB-3. The secondary structures of conserved sequence motifs were predicted by the online Mfold web server (http://www.unafold.org/).

Comparative mitogenomics of carangids revealed high variability in the mitochondrial control region due to length variations and the accumulation of base substitutions and indels. Consistent with the recognition sites in many marine fish^69^ we have detected the 3 central conserved sequence blocks (CSB- F, CSB- E, and CSB-D) and 3 conserved sequence blocks (CSB- 1, CSB-2, and CSB- 3) in all taxa. Palindromic sequence motifs—'TACAT' and 'ATGTA' were found in multiple copies distributed towards the 5' end of the control region [Fig. 2; Supplementary Table S9] and are believed to act as the termination site of heavy strand replication^69^. The segment corresponding to Extended Termination Associated Sequence (ETAS) previously reported in *Scomberoides commersonianus*, a member of the Carangidae^25^, could not be aligned to the corresponding segment in the control region of either species.

No tandem repeat sequences were detected in the control region of Carangidae, but the T homopolymer of 5–12 nucleotides between CSB-D and CSB-1 was found in all taxa. Along with these discontinuous multiple interval repeats of ATATTA and TATAAT were also observed in the control region of Carangidae.

All species except *D. maruadsi* displayed substantial differences from the key sequences of CSB- E^25^ and all were substitutions ranging from 1 to 9 nucleotides. Furthermore, *M. cordyla, P. dentex,* and *Caranx* spp. lacked a 'GTGGG'- box which is a typical feature of CSB- E in teleosts^69^. The CSB- D was highly conserved among carangids and exhibited high sequence similarity between species (Table 4). Although the functions of the CSBs remain unclear, CSB-D is highly conserved in fish families and is involved in regulation of H-strand replication and initiation of D-loop structure^70^ and could play a potential role in mitochondrial metabolism^66^. Conserved sequence blocks (CSB-1,-2, and -3) exhibit a higher level of divergence in carangids with interspecies substitutions, insertions, and deletions (Table 4). Except for *P. dentex*, S. *nigrofasciata, S. leptolepis* and *Seriola* spp. other species showed a characteristic sequence at the 3' end (Table 4). These variations imply rapid evolution of the primary structure in the Carangidae control region, which can provide information for understanding their structural and functional relationship. Furthermore, these conserved regions are involved in the formation of secondary structures in the control region (Fig. 2) that serve as the target sites for proteins or enzymes^69^. These conserved sequence motifs formed similar secondary structures in Carangidae although their primary sequences are not conserved. Therefore, the data reinforce that although there is sequence divergence in the conserved sequence motif, their structural elements and locations appeared to be conserved in Carangidae and some selective constraints on these regions acted to maintain their structure and function.

**Table 4:** The comparison of Central conserved blocks, Conserved sequence blocks and 3’ terminal region in 37 species of Carangidae.

Species	Central conserved blocks		
	CSB- F	CSB- E	CSB- D
Reference sequence	ATGTAGTAAGAACCTACCA	AGGGACAACTATTGTGGGGGT	TATTCCTGGCATTTGGTTCCTA
*Dr*	*******************	************C********	************C*********
*Ac*	***********G*******	*******GAA***********	**********************
*Ai*	*******************	********************G	**********************
*Ad*	*******************	********TA***********	**********************
*Ak*	*******************	********AA***********	**********************
*Am*	*******************	*******GAA***********	**********************
*Ca*	*******************	********AA***********	**********************
*Cb*	*******************	*******GAA***********	**********************
*Ce*	*******************	********AA*CC********	**********************
*Cm*	*******************	*A******AA******A****	**********************
*Cp*	GCCC*A*************	*******GAA***********	**********************
*Ci*	**A****G*AGG*TC****	***A*****C***ACATTC*C	**********************
*Ct*	*C******G**C*****GG	*A******AA**C***A****	****************C*****
*Cme*	*C******G**C*****GG	*A******AA******A****	****************C*****
*Dmac*	*******************	********AA***********	************C*********
*Dm*	**************G****	********AA***********	**********************
*Dmar*	*******************	*********************	************C*********
*Dt*	*******************	********AA**C********	**********************
*Eb*	***C***************	*******GTA***********	**********************
*Gs*	***********G*******	*******GAA***********	****************C*****
*Mc*	*C*C**********C****	*A*****GAA******A****	****************C*****
*Pn*	*******************	********AA***********	**********************
*Pd*	*******************	********AA*****A*****	**********************
*Tt*	***********G*******	********AA**C********	**********************
*Tj*	***********G*******	********AA**C********	**********************
*Sc*	*******************	G*A****GAA**C********	**********************
*Sd*	*CTC*A********G****	*******GAA**C********	**********************
*Sl*	*CTC*A********G****	*******GTA***********	**********************
*Sq*	*CCC*A********G****	********TA**C********	**********************
*Sle*	*******************	*A*****GAA***********	**********************
*Sr*	*******************	*******GAA***********	**********************
*Sn*	*CCC*A********G****	********TA**C********	**********************
*Tb*	***********G*******	********AA**C********	**********************
*To*	GCAC*******GA*C****	********A*C**********	**********************
*Tc*	***********G*******	********TA***********	**********************
*Uh*	*******************	*******GAA***********	**********************
*Us*	*******************	******AGAA***********	**********************

Species	Conserved sequence blocks		
	CSB- 1	CSB- 2	CSB- 3
Reference sequence	CATATTAGGATATCAAGAGCATAA	GTAAAACCCCCCTACCCCCC	TCCTGAAAACCCCCCGGAAAAAA
*Dr*	************************	**T*****************	********************C*G
*Ac*	*****A*A*********TA*****	**T*****************	********************C*G
*Ai*	*****************T******	**T*****************	**T*****************C*G
*Ad*	****C*TT****************	********************	**T*****************C*G
*Ak*	******T*****************	*******************T	********************C*G
*Am*	******T*****************	********************	********************C*G
*Ca*	*****************TA*****	********************	********************C*G
*Cb*	*****************T******	********************	A*******************C*G
*Ce*	*****A***********GA*****	*****A******C******T	***************A****C*G
*Cm*	*****************T******	********************	********************C*G
*Cp*	*****************T******	********************	********************C*G
*Ci*	*******TT***C***********	******AC******C*******	********************C*G
*Ct*	*******TTT**************	********************	********************C*G
*Cme*	*******TTT**************	*****A**************	********************C*G
*Dmac*	************************	*******************T	********************C*G
*Dm*	************************	*******************T	********************C*G
*Dmar*	************************	********************	********************C*G
*Dt*	************************	*******************T	********************C*G
*Eb*	*****CT*****************	**T*****************	********************C*G
*Gs*	**C****AT**********T******	********************	********************C*G
*Mc*	*******TA***************	**T*****************	********************C*G
*Pn*	*****ATT*********T******	**T*********C******T	********************C*G
*Pd*	***C***A*********GA*****	C*C**********C******	*T**A**G*T****GAA*GG*C*
*Tt*	************************	*******************T	********************C*G
*Tj*	************************	*******************T	********************C*G
*Sc*	****CGT******************	************CCA*****	********************C*G
*Sd*	******G*****************	T*******************	*****************G***C*
*Sl*	************************	******A**************	*********A****T*******C*
*Sq*	*******A*******T********	******A**************	*********A****TT******C*
*Sle*	**C****CT***************	********************	********************C*G
*Sr*	*****A******************	T*******************	*****************G***C*
*Sn*	*****AGA*******T********	********************	****************T***C*G
*Tb*	****A*T**********T******	***G*****************	********************C*G
*To*	****A*TA*********T******	************C********	********************C*G
*Tc*	G*C****ATA*********T******	******A**************	********************C*G
*Uh*	************************	*******************T	********************C*G
*Us*	************************	*******************T	********************C*G

Species	3’ end terminal sequence		
Reference sequence	TATTATAATATTTCACAT		
*Dr*	*****C**********		
*Ac*	****************		
*Ai*	****************		
*Ad*	****************		
*Ak*	****************		
*Am*	****************		
*Ca*	****************		
*Cb*	****************		
*Ce*	****************		
*Cp*	****************		
*Cm*	****************		
*Dmac*	*****C**********		
*Dm*	*****C**********		
*Dmar*	*****C**********		
*Dt*	*****C**********		
*Eb*	*****A**********		
*Gs*	****************		
*Mc*	****************		
*Pn*	****************		
*Pd*	absent		
*Ci*	****************		
*Ct*	****************		
*Cme*	****************		
*Tt*	****************		
*Tj*	****************		
*Sc*	*****C*********T**		
*Sd*	Absent		
*Sl*	Absent		
*Sq*	Absent		
*Sle*	Absent		
*Sr*	Absent		
*Sn*	Absent		
*Tb*	************G**T**		
*To*	*************T**		
*Tc*	************G**T**		
*Uh*	****************		
*Us*	****************		

*Dr* = *D. russelli, Ac* = *A. ciliaris, Ai* = *A. indica, Ad* = *A. djedaba, Ak* = *A. kleinii, Am* = *A. mate, Ca* = *C. armatus, Cb* = *C. bajad, Ce* = *C. equula, Cm* = *C. malabaricus, Cp* = *C. plagiotaenia, Ci* = *C. ignobilis, Ct* = *C. tille, Cmel* = *C. melampygus, Dmac* = *D. macerellus, Dm* = *D. macrosoma, Dmar* = *D. maruadsi, Dt* = *D. tabl, Eb* = *E. bipinnulata, Gs* = *G. speciosus, Mc* = *M. cordyla, Pn* = *P. niger, Pd* = *P. dentex, Tt* = *T. trachurus, Tj* = *T. japonicas, Sc* = *S. crumenophthalmus, Sd* = *S. dumerili, Sl* = *S. lalandi, Sq* = *S. quinqueradiata, Sle* = *S. leptolepis, Sr* = *S. rivoliana, Sn* = *S. nigrofasciata, Tb* = *T. blochii, To* = *T. ovatus, Tc* = *T. carolinus, Uh* = *U. helvola, Us* = *U. secunda.*

Asterisks indicate the same nucleotide as the reference sequence. Insertions are represented as bold and deletions as underlined. Other nucleotides denote base substitutions.

#### Carangidae mitogenome sequence comparison

We compared the complete mitochondrial genome of *D. russelli* with other available mitogenomes of the Carangidae family. The DNA coding sequences showed high interspecies identity (Supplementary Figs. S8, S9). The control region contributed to a larger percentage difference between the nucleotide sequences. It has been found that *ND1*, *COI*, *COII* and *COIII* are highly conserved among the DNA coding sequences. In addition, ATP8 was the least conservative of all species, in good agreement with the results obtained from analysis of Ka/Ks and overall mean p genetic distance. With the exception of ND6 and Cyt-b, other PCGs expressed orthologous group (COG) clusters. A total of 12 COGs of proteins were detected from11 PCGs. *ND5* was determined to have 2 COGs, pCOG, and cCOG, and the remaining 10 PCGs each expressed a single COG each (cCOG). COGs are a group of proteins inferred to be orthologous i.e. they are direct evolutionary counterparts^71^. Lineage-specific duplications (orthologous relationships) exist mainly between gene (protein) families rather than between individual proteins^72^. The identification of COGs associated with specific PCGs could indicate the presence of a specific protein in a specific species, and it is crucial to determine the functional relevance of these proteins in that specific species^73^. The results indicated that the COGs present in carangids are involved in inorganic ion transport and metabolism (pCOG) and energy production and conservation (cCOG)^72^. The ND5 gene has been found to perform these two functions, while energy metabolism is the sole function of the remaining 10 PCGs.

#### Phylogenetic analysis

**Figure 3:**
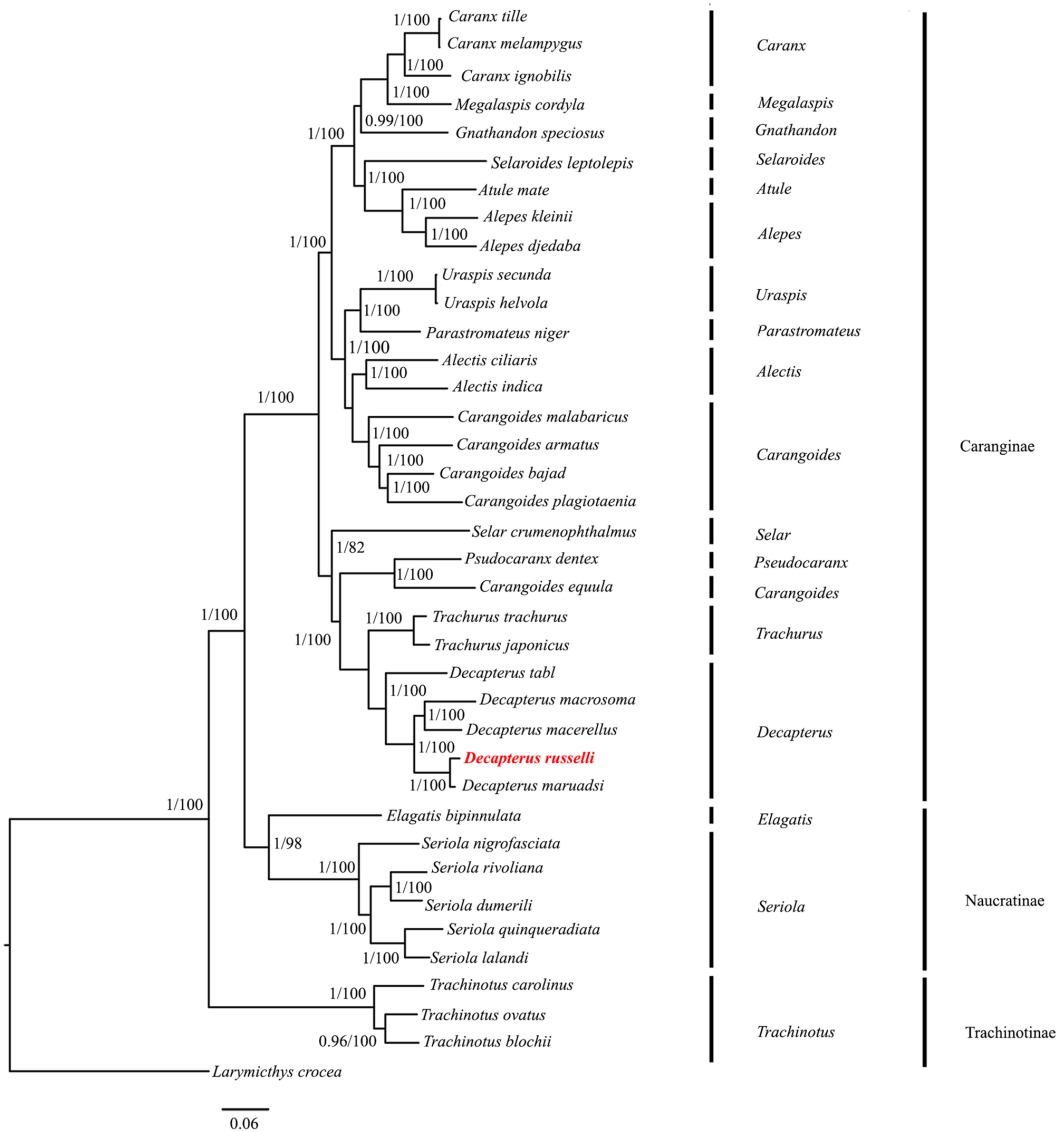
Phylogenetic tree inferred from the nucleotide sequences of 13 concatenated PCGs of 37 species of Carangidae. Bayesian posterior probabilities (left) and bootstrap support values (right) are superimposed with each node. The phylogenetic tree was generated by using MrBayes 3.2.7a^93^.

The phylogenetic position of *D. russelli* is shown in Fig. 3 relative to 36 species of carangids based on 1380 bp sequences. Analogous topologies were obtained for both maximum likelihood analysis (ML) and Bayesian inference (BI) with almost 100% bootstrap support and strong posterior probability values, respectively (Fig. 3). The result implied that 37 species formed 3 lineages corresponding to the subfamilies Caranginae, Naucratinae, and Trachinotinae. The reconstructed phylogenetic tree places *D. russelli* in the same branch with other *Decapterus* spp. and forms a sister group to the genus *Trachurus* and the findings support the previous classification of carangids^44,74^. The phylogenetic tree recovered the three carangid subfamilies as monophyletic and it also supported the groupings (Trachinotinae + (Naucratinae + Caranginae)). This result confirmed the phylogenetic relationship of carangids described by previous findings based on morphological synapomorphic characters^4,31^ and partial sequences of mitochondrial genes^32,75^. Within the Naucratinae, the monotypic *E. bipinnulata* branched individually early from *Seriolina* and *Seriola* spp. After diverging from *Seriolina,* all *Seriola* species formed a monophyletic clade. Furthermore, both phylogenetic trees indicated two well-separated lineages in Caranginae. The first lineage included the genus *Selar, Trachurus,* and *Decapterus.* This lineage also encompassed the species *C. equula* on the same branch with *P. dentex* in contrast to other *Carangoides* Spp. which were grouped into a different branch (Fig. 3). This result confirms the findings of previous studies^44,76^ and thus provides compelling evidence for the assignment of *C. equula* to its previously described genus, *Kaiwarinus*. The second lineage was the most taxonomically diverse group of the family Carangidae delineated in the present study as it consisted of 10 genera divided into two larger groups. The first lineage comprised the genera: *Caranx* + *Megalaspis* + *Gnathandon* as well as *Atule* + *Alepes.* The latter also included the monotypic *S. leptolepis* which individually branched off from this lineage early. The second lineage included *Uraspis* + *Parastromateus* and *Alectis* + *Carangoides.* In addition, a paraphyletic relationship between *Caranx* + *M. cordyla, Alepes* + *A. mate,* and *Uraspis* + *P. niger* was also observed*.*

#### Divergence time estimation

**Figure 4:**
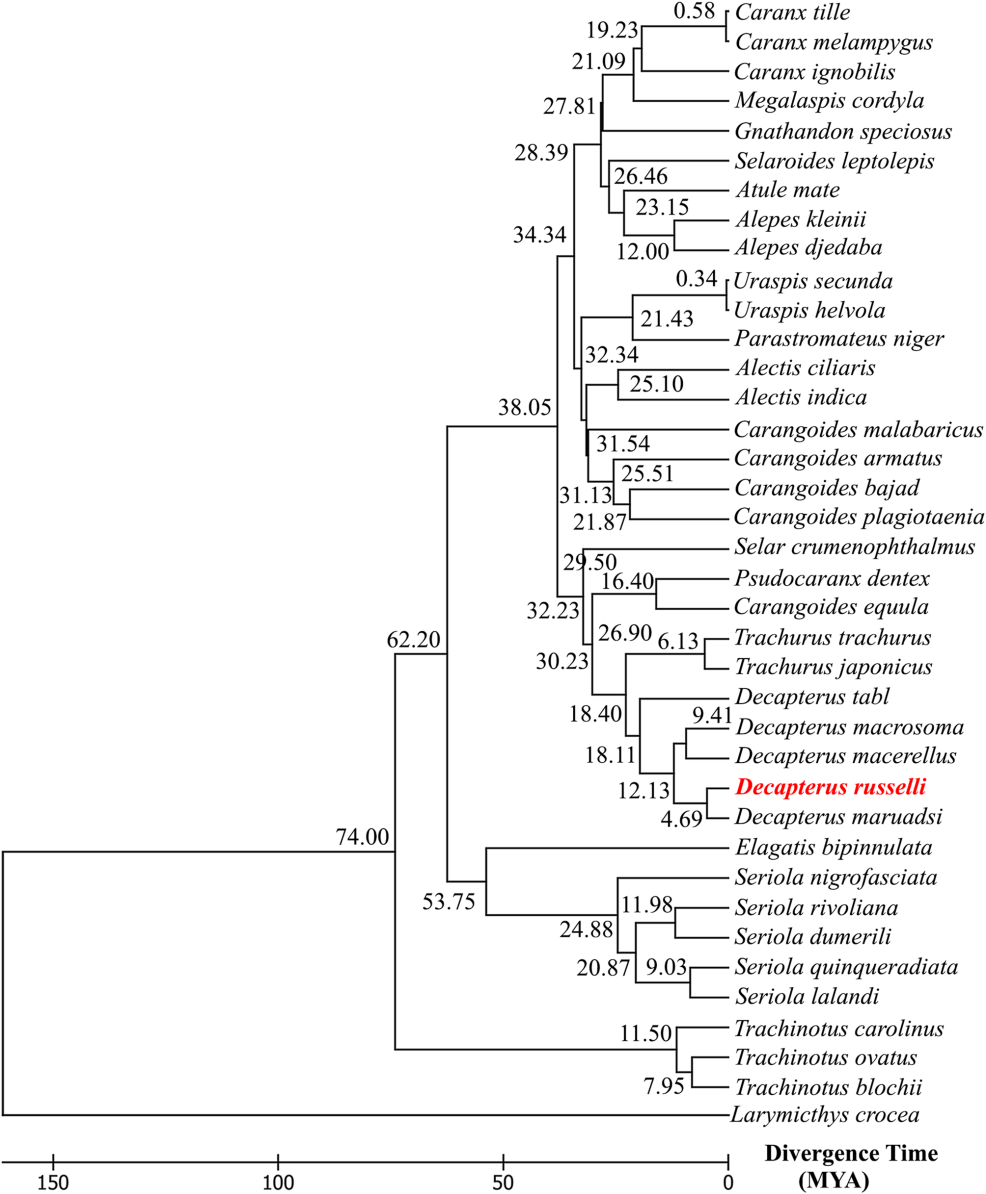
Timetree chronogram of 37 species of Carangidae for the 13 PCGs inferred from Bayesian topology. Divergence times were estimated using 32 calibrations. The red text indicates the species sequenced in this study. The Timetree was generated by using MEGA X^96^.

The RelTime ML analysis revealed that the differentiation of *D. russelli* could be dated back to about 4.69 Mya in the early Pliocene. The family Carangidae had diverged from other lineages when the Cretaceous–Paleogene extinction event occurred and it was estimated to be around 79 Mya towards the end of the cretaceous (Supplementary Fig. S10). It was observed that the separation of the two groups, Trachinotinae and (Caranginae + Naucratinae) occurred at 74 Mya at the end of the Cretaceous. Further, the split between the subfamilies Caranginae and Naucratinae occurred at 62.20 Mya (Early Paleocene). Although the diversification of most species after the Cretaceous mass extinction occurred in the Paleogene^75^, several more recent Neogene radiations can also be observed (Fig. 4).The most recent split was observed at 0.34 Mya between the two *Uraspis* species. Our analyzes of the diversification of the genus Trachurus (18.40 Mya, early Miocene) were consistent with previous studies based on the data of morphological characters and ecological aspects^77^ and cyt b gene sequence analysis^78^. The split between *Seriola* + *Seriolina* and *Elgatis* occurred during the early Paleocene, consistent with Swart et al.^40^ but much younger than Le et al.^44^. The radiation of *Seriola* appears to be 20.87 Mya which is in direct agreement with Le et al.^44^, but slightly predates the estimates by Santini and Carnevale^38^.

## Conclusion

The present investigation focused on the characterization of the complete mitochondrial genome of the Indian Scad, *D. russelli*, and a comprehensive comparative analysis of Carangidae with special emphasis on the structure and variability of tRNA and control region. The carangid tRNA was structurally different in both the stem and loop, with the exception of tRNA-Trp, and tRNA-Leu (TAG). These structural features resulted in mismatched or wobble base pairs in tRNAs. The high rate of Watson–Crick and Wobble base pairing in the stem regions of tRNA suggested their role in stem structure maintenance. The classical cloverleaf secondary structure was retained in all tRNAs except tRNA-Ser (GCT). The presence of an mTERF binding site and a very high evolutionary conservation of its sequence suggest the functional significance of these sites. The high rate of sequence divergence in the flanking regions of conserved sequence motifs indicated the absence of typical mitochondrial control region coding constraints. The presence of evolutionarily conserved secondary structures of the conserved sequence motifs despite sequence divergence revealed the effect of selective constraints on their maintenance. The length variations in protein-coding, tRNA, and rRNA genes among Carangidae were negligible.

The phylogeny inferred from 13 gene concatenated data set resolved the monophyletic lineage of each subfamily and robustly supported the grouping (Trachinotinae + (Caranginae + Naucrautinae)) with the caveat that the subfamily Scomberoidinae could not be included as the mitogenome sequences were not available. Further, the speciation of the genus *Decapterus* occurred before the separation of Tethys Sea and *D. russelli* diverged before the mid-Pliocene warm period. Future studies involving the mitogenomic data of Scomberoidinae are required for a better understanding of the phylogenetic and evolutionary relationship between major groups within the Carangidae. Mitogenomic phylogeny is also influenced by the habitat characteristics of each group, as habitat-specific selection and adaptation in mitochondrial coding genes has recently been reported^79^. The subfamilies Trachinotinae, Naucratinae, and Caranginae consist of species with differential habitat preferences (pelagic, benthopelagic, and reef-associated) and schooling behavior. The mutational patterns in the protein-coding regions of these fish need to be further analyzed for adaptive variation and selection and correlated with habitat characteristics to understand the forces of selection, if any.

## Materials and methods

### Sampling

The wild specimen of *D. russelli* was collected from Kochi, Kerala (9°56′19"N 76°15′45" E) from trawl and purse seines. The species was confirmed based on morphological characters. Tissue samples were preserved in 95% ethanol for DNA extraction and further analysis. The fish sample used in this study was handled according to the guidelines for the care and use of fish in research by De Tolla et al.^80^*.* The protocols were approved by the ethical committee of the ICAR- Central Marine Research Institute, Kochi. These methods are also reported following ARRIVE guidelines (http://arriveguidelines.org).

### DNA extraction and PCR amplification

Genomic DNA was isolated as previously described^81^. The extracted DNA was then eluted in T.E buffer (1X) and stored at − 20 °C for later use as a PCR template. The quality and quantity (concentration) of the isolated total DNA was checked by gel electrophoresis and NanoDrop One Spectrophotometer (Thermo Scientific, USA), respectively. The complete mitochondrial genome of *D. russelli* was determined by combining the sequences of contiguous and overlapping fragments amplified by PCR using both universal as well as custom-designed primers. Initial amplifications were performed using mitochondrial universal primer sequences (FishF1 + FishR1, 16SaRL + 16SbRH, 12S1 + 12S2, Dloop-Thr-F + Dloop-Phe-R, ATP 8.2 L8331 + CO3.2 H9236)^82–86^. The sequenced products of this reaction then served as a template for subsequent amplification. Fifteen sets of new primer pairs were designed based on the aligned complete mitogenome sequences of 36 species of carangids using Primer 3 software (http://frodo.wi.mit.edu/primer3/)^87^. The above primers were screened for potential secondary targets using Primer Blast (https://www.ncbi.nlm.nih.gov/tools/primer-blast/)^88^. PCR amplifications were performed in a Biorad T100 thermal cycler (Bio-Rad, USA). The reaction mixture included 15.6 µl sterile deionized water, 2 µl buffer (10X), 0.4 µl dNTP mix (10 mM each), 0.4 µl of each oligonucleotide (10 µM), 1 µl of template DNA (50 ng/µl) and 0.2 µl (1 unit) of Taq DNA polymerase with a total reaction volume of 20 µl. For confirmation, PCR products were electrophoresed on 1.5% agarose gel (1X TBE) and visualized using a gel documentation system (Bio-Rad, USA). The products were then sequenced completely in both directions using sanger sequencing.

### Sequence analysis

Target sequences were assembled with the software MEGA X^89^. The identity of the *D. russelli* mitogenome was verified with other carangid mitogenomes (Supplementary Table S2) by BLAST search. These mitogenome sequences served as the reference for assembly and manual annotations. The organization and the order of the genes were initially determined using the MitoFish database^90^ and later verified manually by comparison with the reference sequences. The 13 PCGs and their codon usage were summarized with MEGA X^89^. The rate of Ka/Ks substitutions in the mitogenome of Carangidae was calculated by DnaSP 6.12.03^91^. tRNA genes were first annotated by using tRNA scan SE^92^ with default parameters coupled with ARWEN software^57^. The secondary structure of conserved sequence motifs of the control region was inferred using Mfold web server^93^. The complete mitogenome map of the species was graphically visualized by OrganellarGenomeDRAW^94^. The 37 mitogenomes of Carangidae were compared by using CG view comparison tool (CCT)^95^ with *D. russelli* as the reference sequence. Genes were assigned by Clusters of Orthologous Groups (COG) using the CG view comparison tool (CCT) and BLAST was used to align other genomes to that of *D. russelli*.

### Phylogenetic analysis

All available mitogenome sequences of other carangid species were retrieved from GenBank to find out the relative position of *D. russelli* among carangids and to delineate its phylogenetic relationship. *L. crocea* (GenBank accession number **NC011710**) was taken as an outgroup. The 13 PCGs were aligned using Clustal W and translated into amino acid sequences in MEGA X^57^. For the subsequent phylogenetic analysis, multiple alignments were generated for the 13 concatenated protein-coding sequences of *D. russelli* and 36 other Carangidae species. The phylogenetic relationship was inferred by constructing a tree based on Bayesian inference (BI) and maximum likelihood (ML) methods. The best model, GTR + G + I, was selected using Akaike Information Criterion (AIC). Bayesian phylogenetic analysis was performed using MrBayes^96^ with the Markov Chain Monte Karlo Method (MCMC). MCMC chains were run for 10 million generations and obtained a standard deviation of split frequency less than 0.01. The ML phylogenetic tree was inferred using RaxML^97^ and a consensus tree was obtained with 1000 bootstrap replicates using default parameters. The final phylogenetic tree was displayed, annotated, and embellished in FigTree software (http://tree.bio.ed.ac.uk/software/figtree/)^98^.

### Divergence time estimation

Molecular dating of 37 Carangidae species was performed with the RelTimeML method in MEGA X^99^ using GTR + G + I modeling. The phylogenetic tree inferred from Bayesian analysis was imported to MEGA X to estimate divergence times. The time tree web resource (http://www.timetree.org/)^100^ was used to infer calibration boundaries for a pair of taxa (minimum and maximum) and the same database was used to establish the time tree of 27 genera of carangids.

## Supplementary Information

Supplementary Information.

## Data Availability

All data generated or analyzed during this study is included in this published article (and its Supplementary information files).
