# Supplementary material for: Comparative mitogenomics and phylogenetics of the family Carangidae with special emphasis on the mitogenome of the Indian Scad Decapterus russelli
Source: Sci Rep. 2022 Apr 4;12:5642. doi: 10.1038/s41598-022-09636-5 (PMC8980026; doi:10.1038/s41598-022-09636-5)
Supplement: Supplementary file 1 — Supplementary Information. [file 41598_2022_9636_MOESM1_ESM.pdf]

Comparative mitogenomics and phylogenetics of the family Carangidae with special emphasis on the mitogenome of the Indian Scad *Decapterus russelli*

Anjaly Jose[1,2*], Sandhya Sukumaran[1], Lakshmi P. Mukundan[1], Neenu Raj[1], Sujitha Mary[1], Nisha. K[1], A. Gopalakrishnan[1]

[1]Marine Biotechnology Division, ICAR-Central Marine Fisheries Research Institute, Ernakulam North P O, Kochi, Kerala, India-682018
[2]Mangalore University, Mangalagangotri, Mangalore, Karnataka, India-574199

*corresponding author

Email address:anjalijose2@gmail.com

Table S1. Comparison of gene rearrangements in light (-) and heavy (+) strand within 37 Carangidae species. *Dr=D. russelli, Ac= A. ciliaris, Ai= A. indica, Ad= A. djedaba, Ak= A. kleinii, Am= A. mate, Ca= C. armatus, Cb= C. bajad, Ce= C. equula, Cm=c. malabaricus, Cp= C. plagiotaenia, Ci= C. ignobilis, Cmel=C. melampygus, Ct= C. tille, Dmac = D. macerellus, Dm= D. macrosoma, Dmar= D. maruadsi,, Dt= D. tabl, Eb= E. bipinnulata, Gs= G. speciosus, Mc= M. cordyla, Pn= P. niger, Pd= P. dentex, Sc=S. crumenophthalmus, Sle= S. leptolepis, Sd= S. dumerili, Sl=S. lalandi, Sq=S. quinqueradiata, Sr= S. rivoliana, Sn= S. nigrofasciata, Tb=T. blochii, Tc=T. carolinus, To= T. ovatus, Tj= T. japonicas, Tt= T. trachurus, Uh= U. helvola, Us= U. secunda.*

Species	F	12S	V	16S	L	ND 1	I	Q	M	ND 2	W	A	N	C	Y	CO 1	S	D	CO 2	K	ATP8	ATP6	CO 3	G	ND 3	R	ND4L	ND 4	H	S	L	ND 5	N D6	E	Cyt b	T	P	D loop
Dr	+	+	+	+	+	+	+	-	+	+	+	-	-	-	-	+	-	+	+	+	+	+	+	+	+	+	+	+	+	+	+	+	-	-	+	+	-	+
Ac	+	+	+	+	+	+	+	-	+	+	+	-	-	-	-	+	-	+	+	+	+	+	+	+	+	+	+	+	+	+	+	+	-	-	+	+	-	+
Ai	+	+	+	+	+	+	+	-	+	+	+	-	-	-	-	+	-	+	+	+	+	+	+	+	+	+	+	+	+	+	+	+	-	-	+	+	-	+

Ad	+	+	+	+	+	+	+	-	+	+	+	-	-	-	-	+	-	+	+	+	+	+	+	+	+	+	+	+	+	+	+	+	-	-	+	+	-	+
Ak	+	+	+	+	+	+	+	-	+	+	+	-	-	-	-	+	-	+	+	+	+	+	+	+	+	+	+	+	+	+	+	+	-	-	+	+	-	+
Am	+	+	+	+	+	+	+	-	+	+	+	-	-	-	-	+	-	+	+	+	+	+	+	+	+	+	+	+	+	+	+	+	-	-	+	+	-	+
Ca	+	+	+	+	+	+	+	-	+	+	+	-	-	-	-	+	-	+	+	+	+	+	+	+	+	+	+	+	+	+	+	+	-	-	+	+	-	+
Cb	+	+	+	+	+	+	+	-	+	+	+	-	-	-	-	+	-	+	+	+	+	+	+	+	+	+	+	+	+	+	+	+	-	-	+	+	-	+
Ce	+	+	+	+	+	+	+	-	+	+	+	-	-	-	-	+	-	+	+	+	+	+	+	+	+	+	+	+	+	+	+	+	-	-	+	+	-	+
Cm	+	+	+	+	+	+	+	-	+	+	+	-	-	-	-	+	-	+	+	+	+	+	+	+	+	+	+	+	+	+	+	+	-	-	+	+	-	+
Cp	+	+	+	+	+	+	+	-	+	+	+	-	-	-	-	+	-	+	+	+	+	+	+	+	+	+	+	+	+	+	+	+	-	-	+	+	-	+
Ci	+	+	+	+	+	+	+	-	+	+	+	-	-	-	-	+	-	+	+	+	+	+	+	+	+	+	+	+	+	+	+	+	-	-	+	+	-	+
Cmel	+	+	+	+	+	+	+	-	+	+	+	-	-	-	-	+	-	+	+	+	+	+	+	+	+	+	+	+	+	+	+	+	-	-	+	+	-	+
Ct	+	+	+	+	+	+	+	-	+	+	+	-	-	-	-	+	-	+	+	+	+	+	+	+	+	+	+	+	+	+	+	+	-	-	+	+	-	+
Dmac	+	+	+	+	+	+	+	-	+	+	+	-	-	-	-	+	-	+	+	+	+	+	+	+	+	+	+	+	+	+	+	+	-	-	+	+	-	+
Dm	+	+	+	+	+	+	+	-	+	+	+	-	-	-	-	+	-	+	+	+	+	+	+	+	+	+	+	+	+	+	+	+	-	-	+	+	-	+
Dmar	+	+	+	+	+	+	+	-	+	+	+	-	-	-	-	+	-	+	+	+	+	+	+	+	+	+	+	+	+	+	+	+	-	-	+	+	-	+
Dt	+	+	+	+	+	+	+	-	+	+	+	-	-	-	-	+	-	+	+	+	+	+	+	+	+	+	+	+	+	+	+	+	-	-	+	+	-	+
Eb	+	+	+	+	+	+	+	-	+	+	+	-	-	-	-	+	-	+	+	+	+	+	+	+	+	+	+	+	+	+	+	+	-	-	+	+	-	+
Gs	+	+	+	+	+	+	+	-	+	+	+	-	-	-	-	+	-	+	+	+	+	+	+	+	+	+	+	+	+	+	+	+	-	-	+	+	-	+
Mc	+	+	+	+	+	+	+	-	+	+	+	-	-	-	-	+	-	+	+	+	+	+	+	+	+	+	+	+	+	+	+	+	-	-	+	+	-	+
Pn	+	+	+	+	+	+	+	-	+	+	+	-	-	-	-	+	-	+	+	+	+	+	+	+	+	+	+	+	+	+	+	+	-	-	+	+	-	+
Pd	+	+	+	+	+	+	+	-	+	+	+	-	-	-	-	+	-	+	+	+	+	+	+	+	+	+	+	+	+	+	+	+	-	-	+	+	-	+
Sc	+	+	+	+	+	+	+	-	+	+	+	-	-	-	-	+	-	+	+	+	+	+	+	+	+	+	+	+	+	+	+	+	-	-	+	+	-	+
Sle	+	+	+	+	+	+	+	-	+	+	+	-	-	-	-	+	-	+	+	+	+	+	+	+	+	+	+	+	+	+	+	+	-	-	+	+	-	+
Sd	+	+	+	+	+	+	+	-	+	+	+	-	-	-	-	+	-	+	+	+	+	+	+	+	+	+	+	+	+	+	+	+	-	-	+	+	-	+
Sl	+	+	+	+	+	+	+	-	+	+	+	-	-	-	-	+	-	+	+	+	+	+	+	+	+	+	+	+	+	+	+	+	-	-	+	+	-	+
Sq	+	+	+	+	+	+	+	-	+	+	+	-	-	-	-	+	-	+	+	+	+	+	+	+	+	+	+	+	+	+	+	+	-	-	+	+	-	+
Sr	+	+	+	+	+	+	+	-	+	+	+	-	-	-	-	+	-	+	+	+	+	+	+	+	+	+	+	+	+	+	+	+	-	-	+	+	-	+

Sn	+	+	+	+	+	+	+	-	+	+	+	-	-	-	-	+	-	+	+	+	+	+	+	+	+	+	+	+	+	+	+	+	-	-	+	+	-	+
Tb	+	+	+	+	+	+	+	-	+	+	+	-	-	-	-	+	-	+	+	+	+	+	+	+	+	+	+	+	+	+	+	+	-	-	+	+	-	+
Tc	+	+	+	+	+	+	+	-	+	+	+	-	-	-	-	+	-	+	+	+	+	+	+	+	+	+	+	+	+	+	+	+	-	-	+	+	-	+
To	+	+	+	+	+	+	+	-	+	+	+	-	-	-	-	+	-	+	+	+	+	+	+	+	+	+	+	+	+	+	+	+	-	-	+	+	-	+
Tj	+	+	+	+	+	+	+	-	+	+	+	-	-	-	-	+	-	+	+	+	+	+	+	+	+	+	+	+	+	+	+	+	-	-	+	+	-	+
Tt	+	+	+	+	+	+	+	-	+	+	+	-	-	-	-	+	-	+	+	+	+	+	+	+	+	+	+	+	+	+	+	+	-	-	+	+	-	+
Uh	+	+	+	+	+	+	+	-	+	+	+	-	-	-	-	+	-	+	+	+	+	+	+	+	+	+	+	+	+	+	+	+	-	-	+	+	-	+
Us	+	+	+	+	+	+	+	-	+	+	+	-	-	-	-	+	-	+	+	+	+	+	+	+	+	+	+	+	+	+	+	+	-	-	+	+	-	+

Table S2. Nucleotide composition and skewness level of the mitogenomes in different Carangidae species

Speies	Accession No.	Length	Whole genome composition						
			A%	G%	T%	C%	A+T	AT skew	GC skew
Decapterus ruselli	MN_711693	16542	27.5	16.9	25.4	30.2	52.9	0.039	−0.282
Alectis ciliaris	NC_025566	16,570	28.3	16.2	26.8	28.8	55.1	0.027	−0.280
Alectis indica	KP_710215	16553	28	16.2	25.6	30.2	53.6	0.050	-0.200
Alepes djedaba	KP_408222	16,563	27.9	16.7	26.4	29	54.3	0.027	−0.269
Alepes kleinii	NC_023524	16,571	28.1	16.5	27	28.5	55.1	0.019	−0.266
Atule mate	NC_026222	16,565	28.4	16.3	27.6	27.7	56	0.014	-0.260
Carangoides armatus	NC_004405	16,556	28	16.1	26.5	29.4	54.5	0.027	−0.292
Carangoides bajad	LC_557137	16,556	28.4	15.8	26.1	29.8	54.5	0.043	-0.307
Carangoides equula	NC_025644	16,588	26.3	18.1	25.3	30.2	51.6	0.019	−0.250
Carangoides malabaricus	NC_023968	16,561	27.8	16.4	26.2	29.6	53.6	0.037	−0.286
Carangoides plagiotaenia	MT_677872	165551	28.2	15.9	26.7	29.1	54.9	0.027	-0.293
Carannx ignobilis	NC_022932	16,588	28.8	16	25.8	29.3	54.6	0.054	−0.293
Caranx melampygus	AP_004445	16,593	28.9	15.8	26.3	28.9	55.2	0.047	−0.293
Caranx tille	NC_029421	16,593	28.9	15.8	26.3	28.9	55.2	0.0471	−0.293
Decapterus macarellus	NC_026718	16,544	27.3	17.0	25.3	30.4	52.6	0.038	−0.282
Decapterus macrosoma	KF_841444	16,545	27	17.2	25.4	30.4	52.4	0.030	−0.277
Decapterus maruadsi	NC_024556	16,541	27.5	16.8	25.4	30.2	52.9	0.039	−0.285
Decapterus tabl	MN_102718	16545	27.3	17.1	25	30.6	52.3	0.043	-0.283
Elagatis bipinnulata	NC_029880	16542	27.9	16.8	25.8	29.5	53.7	0.040	-0.274
Gnathandon speciosus	NC_054367	16555	29.4	15.5	26.3	28.7	55.7	0.060	-0.230
Megalaspis cordyla	NC_025565	16,566	28.8	15.9	25.8	29.4	54.6	0.054	−0.298
Parastromateus niger	KJ_102332	16561	28.3	16.2	26	29.5	54.3	0.042	-0.30
Pseudocaranx dentex	MZ_359280	16569	27.2	17.2	25.4	30.2	52.6	0.034	-0.274
Selar crumenophthalmus	NC_023954	16610	27.2	16.8	26.6	29.5	53.8	0.011	−0.274
Selaroides leptolepis	NC_029184	16560	27.8	16.8	26.5	28.9	54.3	0.024	-0.264
Seriola dumerili	AB_517558	16,530	26.8	17.6	25.5	30.1	52.3	0.024	−0.262
Seriola lalandi	AB_517557	16,532	26.7	17.8	25.3	30.2	52	0.026	−0.258
Seriola quinqueradiata	AB_517556	16,537	26.6	18	25.2	30.2	51.8	0.027	−0.253
Seriola rivoliana	NC_027183	16530	27.2	17.2	25.7	29.9	52.9	0.028	-0.269
Seriolina nigrofasciata	NC_028420	16531	26.7	17.5	25.8	30	52.5	0.017	-0.263

Trachinotus blochii	NC_024026	16,558	29.2	15.7	26.5	28.6	55.7	0.048	−0.291
Trachinotus carolinus	NC_024184	16,544	28.7	16.3	26	29.1	54.7	0.049	−0.281
Trachinotus ovatus	KJ_642220	16,564	29	15.9	26.2	28.9	55.2	0.050	−0.290
Trachurus japonicus	AP_003092	16,559	27.8	16.5	25.8	29.9	53.6	0.037	−0.288
Trachurus trachurus	AB_108498	16559	27.7	16.6	25.8	29.9	53.5	0.035	−0.286
Uraspis helvola	NC_033402	16555	28.1	16.2	25.8	29.8	53.9	0.042	-0.030
Uraspis secunda	NC_029488	16554	28.2	16.2	25.8	29.8	54	0.050	-0.030
**Protein coding genes**									
Decapterus ruselli		11425	25.6	15.6	26.1	32.8	51.7	−0.009	−0.355
Alectis ciliaris		11425	26.9	14.6	28.1	30.5	55	−0.021	−0.352
Alectis indica		11428	25.4	15.6	27.6	31.4	53	-0.042	-0.40
Alepes djedaba		11427	26.2	15.2	27.5	31.2	53.7	−0.024	−0.344
Alepes kleinii		11422	26.4	15.0	28.2	30.4	54.6	−0.001	−0.339
Atule mate		11426	25.6	15.6	30.3	28.5	55.9	-0.09	-0.30
Carangoides armatus		11427	26.5	14.6	27.5	31.4	54	−0.018	−0.365
Carangoides bajad		11427	25.7	15.3	28.1	31.0	53.8	-0.050	-0.40
Carangoides equula		11427	24.1	17.2	26.2	32.5	50.3	−0.041	−0.307
Carangoides malabaricus		11428	26.1	15.1	27.1	31.7	53.2	−0.018	−0.354
Carangoides plagiotaenia		11426	25.6	15.4	28.9	30.1	54.5	-0.060	-0.323
Carannx ignobilis		11422	27.4	14.6	26.9	31.1	54.3	−0.009	−0.361
Caranx melampygus		11421	27.6	14.3	27.2	30.8	54.8	−0.018	−0.354
Caranx tille		11422	27.7	14.3	27.2	30.8	54.9	0.009	−0.365
Decapterus macarellus		11425	25.5	15.7	25.8	33	51.3	−0.005	−0.355
Decapterus macrosoma		11425	25	16	26	33	51	−0.019	−0.346
Decapterus maruadsi		11425	25.7	15.4	26.1	32.8	51.8	−0.007	−0.360
Decapterus tabl		11425	24.3	16.5	26.7	32.5	51	-0.050	-0.326
Elagatis bipinnulata		11432	25.3	16.2	27.9	30.7	53.2	-0.050	0.320
Gnathandon speciosus		11416	27	14.7	28.6	29.7	55.6	-0.030	-0.40
Megalaspis cordyla		11422	27.7	14.4	26.6	31.4	54.3	0.020	−0.371
Parastromateus niger		11430	25.7	15.5	28.1	30.6	53.8	-0.050	-0.30
Pseudocaranx dentex		11397	24.2	16.8	27.2	31.8	51.4	-0.058	-0.308
Selar crumenophthalmus		11425	25.6	15.3	27.5	31.6	53.1	−0.035	−0.349
Selaroides leptolepis		11421	25.3	16.2	28.6	29.9	53.9	-0.061	-0.30
Seriola dumerili		11433	25.3	16.2	26.3	32.2	51.6	−0.019	−0.330
Seriola lalandi		11433	25	16.6	26	32.4	51	−0.019	−0.322
Seriola quinqueradiata		11433	24.8	16.9	26	32.3	50.8	−0.023	−0.313
Seriola rivoliana		11429	24.9	16.5	27.6	31.1	52.5	-0.051	-0.030
Seriolina nigrofasciata		11429	24.2	16.9	27.5	31.4	51.7	-0.063	-0.300
Trachinotus blochii		11427	27.5	14.3	27.7	30.5	55.2	−0.003	−0.354
Trachinotus carolinus		11429	26.9	14.9	27.1	31.1	54	−0.003	−0.352
Trachinotus ovatus		11427	27.4	14.4	27.4	30.8	54.8	0.000	−0.362
Trachurus japonicus		11425	26.2	15	26.6	32.2	52.8	−0.007	−0.364
Trachurus trachurus		11424	26.1	15.1	26.6	32.2	52.7	−0.009	−0.361

Uraspis helvola	11422	25.4	15.6	28.2	30.9	53.6	-0.053	-0.329
Uraspis secunda	11428	25.4	15.6	28.1	30.9	53.5	-0.050	-0.329
**tRNA genes**								
Decapterus ruselli	1555	30.5	20.6	23.7	25.1	54.2	0.125	−0.098
Alectis ciliaris	1555	30.6	20.3	24.3	24.8	54.9	0.114	−0.099
Alectis indica	1555	30.5	20.2	23.9	25.3	54.4	0.121	-0.112
Alepes djedaba	1552	30.7	20.4	23.8	25.1	54.5	0.126	−0.103
Alepes kleinii	1557	30.8	20.2	24.1	24.9	54.9	0.122	−0.104
Atule mate	1553	31.1	20	24.1	25.3	55.2	0.126	-0.109
Carangoides armatus	1558	30.5	20.4	24.2	24.9	54.7	0.115	−0.099
Carangoides bajad	1556	30.9	20.0	24.2	24.9	55.1	0.121	-0.109
Carangoides equula	1560	30.9	20.1	23.5	25.4	54.4	0.136	−0.116
Carangoides malabaricus	1554	30.9	20	23.8	25.2	54.7	0.129	−0.115
Carangoides plagiotaenia	1555	31.1	19.9	23.9	25.1	55.0	0.130	-0.115
Carannx ignobilis	1558	31.0	20	24	25	55.0	0.127	−0.111
Caranx tille	1558	30.9	20	24.3	24.7	55.2	0.119	−0.105
Caranx melampygus	1558	30.9	20	24.3	24.7	55.2	0.119	−0.105
Decapterus macarellus	1555	30.5	20.3	23.7	25.4	54.2	0.125	−0.111
Decapterus macrosoma	1556	30.4	20.6	23.7	25.3	54.1	0.123	−0.102
Decapterus maruadsi	1552	30.4	20.7	23.7	25.3	54.1	0.123	−0.100
Decapterus tabl	1555	30.8	20.4	23.7	25.1	54.5	0.130	-0.103
Elgatis bipinnulata	1548	30.8	20.2	23.9	25.1	54.7	0.126	-0.108
Gnathanodon speciosus	1557	31.1	20	24.3	24.5	55.4	0.122	-0.101
Megalaspis cordyla	1552	30.7	20.1	24	25.2	54.7	0.1224	−0.112
Parastromateus niger	1554	29.2	21	23.8	26	53.0	0.101	-0.106
Pseudocaranx dentex	1550	30.7	20.1	24.0	25.2	54.7	0.122	-0.112
Trachurus trachurus	1553	30.7	20.5	24	24.8	54.7	0.122	−0.094
Trachurus japonicus	1556	30.7	20.5	23.9	24.9	54.6	0.124	−0.096
Selar crumenophthalmus	1555	30.2	20.5	24.1	25.2	54.3	0.112	−0.102
Seriola dumerili	1552	29.4	20.8	23.7	26	53.1	0.107	−0.111
Seriola lalandi	1552	29.7	20.6	24	25.8	53.7	0.106	−0.112
Seriola quinqueradiata	1552	29.5	20.9	23.6	26	53.1	0.111	−0.108
Seriola rivoliana	1548	*29.3*	20.8	23.4	26.5	52.7	0.111	-0.120
Seriolina nigrofasciata	1552	*29.4*	21	23.8	25.8	52.9	0.105	-0.102
Selaroides leptolepis	1559	*30.5*	20.3	24.5	24.8	55	0.109	-0.099
Trachinotus blochii	1564	31.9	19.9	23.8	24.4	55.7	0.145	−0.101
Trachinotus carolinus	1554	31.5	20.3	23.8	24.4	55.3	0.139	−0.091
Trachinotus ovatus	1552	31.6	20	20	24.5	51.6	0.224	−0.101
Uraspis helvola	1554	*30.6*	20.2	23.5	25.7	54.1	0.131	-0.119
Uraspis secunda	1556	*30.5*	20.3	23.7	25.6	54.7	0.125	-0.115
**rRNA genes**								
Decapterus ruselli	2677	31.9	21.8	20.8	25.6	53.7	0.188	-0.103
Alectis ciliaris	2667	31.5	21.6	21	25.8	53.1	0.184	-0.102
Alectis indica	2667	31.6	20.9	20.9	26.5	52.5	0.203	-0.118
Alepes djedaba	2680	31.7	22.1	21.2	25	53.8	0.178	-0.082
Alepes kleinii	2683	31.6	22.4	21.1	24.9	54	0.170	-0.082

Atule mate	2681	31.6	21	22.2	25.2	53.8	0.174	-0.090
Decapterus macarellus	2675	31.6	21.6	21.2	25.5	53.2	0.187	-0.092
Decapterus macrosoma	2674	31.9	21.8	20.9	25.4	53.7	0.188	-0.097
Decapterus maruadsi	2674	31.9	21.7	20.9	25.6	53.6	0.190	-0.101
Decapterus tabl	2673	31.7	21.2	21.3	25.8	53	0.196	-0.097
Carangoides armatus	2668	31.9	21.5	20.8	25.7	53.4	0.194	-0.105
Carangoides bajad	2669	32	20.7	21.7	25.7	53.7	0.191	-0.108
Carangoides equula	2669	31.5	21.5	21.5	25.7	52.9	0.190	-0.088
Carangoides malabaricus	2668	32	21.4	20.9	25.7	53.4	0.198	-0.103
Carangoides plagiotaenia	2668	32.1	20.7	21.6	25.7	53.7	0.196	-0.108
Carannx ignobilis	2676	31.9	21.4	20.9	25.8	53.3	0.196	-0.104
Caranx melampygus	2673	32.2	21.3	20.8	25.7	53.5	0.203	-0.105
Caranx tille	2673	32.2	21.3	20.8	25.7	53.5	0.203	-0.105
Elgatis bipinnulata	2664	31.7	21.1	21.3	25.8	53	0.190	-0.100
Gnathanodon speciosus	2655	31.9	20.9	21.6	25.6	53.5	0.196	-0.101
Megalaspis cordyla	2678	31.7	21.8	20.9	25.6	53.5	0.185	-0.101
Parastromateus niger	2672	31.8	20.8	21.4	26	53.2	0.195	-0.111
Pseudocaranx dentex	2668	31.9	21.3	21.4	25.5	53.3	0.196	-0.089
Selar crumenophthalmus	2679	31.5	22	21.2	25.2	53.5	0.177	-0.086
Selaroides leptolepis	2671	31	21.5	21.9	25.6	52.9	0.172	-0.088
Seriola dumerili	2668	30.8	21.3	22.1	25.8	52.1	0.182	-0.077
Seriola lalandi	2668	30.5	21	22.2	26.3	51.5	0.184	-0.084
Seriola quinqueradiata	2673	30.7	21	21.8	26.5	51.7	0.187	-0.097
Seriola rivoliana	2669	30.8	21.9	21.5	25.8	52.3	0.177	-0.081
Seriolina nigrofasciata	2668	30.2	22.2	21.8	25.7	52	0.161	-0.073
Trachinotus blochii	2676	33.6	21.3	19.8	25.3	54.9	0.224	-0.121
Trachinotus carolinus	2672	33.4	21.1	19.9	25.5	54.5	0.225	-0.123
Trachinotus ovatus	2677	33.3	21.1	20	25.7	54.4	0.224	-0.124
Trachurus japonicus	2672	32	21.4	21	25.6	53.4	0.198	-0.098
Trachurus trachurus	2672	31.8	21.6	21.1	25.	53.4	0.191	-0.096
Uraspis helvola	2670	31.7	20.7	21.3	26.3	53	0.196	-0.119
Uraspis secunda	2669	31.7	20.8	21.3	26.2	53	0.196	-0.114
**Control region**								
Decapterus ruselli	840	33	16.3	30.6	20.1	63.6	0.038	-0.104
Alectis ciliaris	878	32	15.1	29.8	23	61.8	0.035	-0.207
Alectis indica	857	32.1	14.6	30.7	22.6	62.8	0.022	-0.215
Alepes djedaba	857	33.7	15.2	30.1	21	63.8	0.056	-0.160
Alepes kleinii	861	33	14.3	31.4	21.4	64.4	0.024	-0.198
Atule mate	858	33.8	14.3	31.2	20.6	65	0.040	-0.180
Carangoides armatus	865	31.7	14.2	32.6	21.5	64.3	-0.019	-0.204
Carangoides bajad	860	31.9	13.5	32.3	22.3	64.2	-0.006	-0.245
Carangoides equula	889	30.7	17	29.6	22.7	60.3	0.018	-0.143
Carangoides malabaricus	866	32	13	32.1	22.9	64.1	-0.001	-0.275
Carangoides plagiotaenia	859	31.2	13.6	33.6	21.5	64.8	-0.037	-0.225
Carannx ignobilis	883	33.6	13.6	28.7	24.1	62.3	0.078	-0.278
Caranx melampygus	893	32.4	13.2	32.9	21.5	65.3	-0.007	-0.239

Caranx tille	893	32.1	13.3	32.7	21.8	64.8	-0.009	-0.242
Decapterus macarellus	845	31.6	16.1	31.6	20.6	63.2	0.00	-0.122
Decapterus macrosoma	845	32.8	15.6	31.4	20.2	64.2	0.021	-0.128
Decapterus maruadsi	842	32.8	16.4	31	19.8	63.8	0.028	-0.093
Decapterus tabl	848	32.5	15.3	30.3	21.8	62.8	0.035	-0.175
Elgatis bipinnulata	855	31.6	15.4	30.6	22.3	62.2	0.016	-0.183
Gnathanodon speciosus	856	34.8	14	30.7	30.7	65.5	0.062	-0.186
Megalaspis cordyla	863	31.9	14	30.9	23.2	62.8	0.015	-0.247
Parastromateus niger	861	32.1	14.9	31.2	21.8	64.2	0.014	-0.188
Pseudocaranx dentex	877	32.7	14.8	29.1	23.4	61.8	0.058	-0.225
Selar crumenophthalmus	904	29.3	15.6	32.2	22.9	61.6	-0.047	-0.189
Selaroides leptolepis	862	31.8	13.7	31.3	23.2	63.1	0.007	-0.257
Seriola dumerili	842	30.3	16.6	31.1	22	61.4	-0.013	-0.139
Seriola lalandi	845	31.6	16.3	31.8	20.3	63.4	-0.003	-0.109
Seriola quinqueradiata	845	31.4	16.6	30.3	21.8	61.7	0.017	-0.135
Seriola rivoliana	841	31.4	16.1	30.2	22.4	61.6	0.019	-0.163
Seriolina nigrofasciata	843	32.1	15.2	31.8	20.9	63.9	0.004	-0.157
Trachinotus blochii	858	34.3	14.6	30.1	21.1	64.4	0.065	-0.182
Trachinotus carolinus	849	32.4	15.7	30.4	21.6	62.8	0.031	-0.158
Trachinotus ovatus	862	32.7	15.2	30.7	21.4	63.4	0.031	-0.169
Trachurus japonicus	862	31.1	15.8	31.3	21.8	62.4	-0.003	-0.159
Trachurus trachurus	862	31.1	16	31	21.9	62.1	0.001	-0.155
Uraspis helvola	861	32.4	14.9	30.5	22.2	62.9	0.030	-0.196
Uraspis secunda	861	32.9	14.5	30.3	22.3	63.2	0.041	-0.211

Table S3. Comparison of overlapping and intergenic spacer regions of 37 Carangidae species. *Dr=D. russelli, Ac= A. ciliaris, Ai= A. indica, Ad= A. djedaba, Ak= A. kleinii, Am= A. mate, Ca= C. armatus, Cb= C. bajad, Ce= C. equula, Cm= C. malabaricus, Cp= C. plagiotaenia, Ci= C. ignobilis, Cmel=C. melampygus, Ct= C. tille, Dmac = D. macerellus, Dm= D. macrosoma, Dmar= D. maruadsi,, Dt= D. tabl, Eb= E. bipinnulata, Gs= G. speciosus, Mc= M. cordyla, Pn= P. niger, Pd= P. dentex, Sc=S. crumenophthalmus, Sle= S. leptolepis, Sd= S. dumerili, Sl=S. lalandi, Sq=S. quinqueradiata, Sr= S. rivoliana, Sn= S. nigrofasciata, Tb=T. blochii, Tc=T. carolinus, To= T. ovatus, Tj= T. japonicas, Tt= T. trachurus, Uh= U. helvola, Us= U. secunda.*

Locus	*Dr*	*Ac*	*Ai*	*Ad*	*Ak*	*Am*	*Ca*	*Cb*	*Ce*	*Cm*	*Cp*	*Ci*	*Cmel*	*Ct*	*Dmac*	*Dm*	*Dmar*	*Dt*	*Eb*	*Gs*	*Mc*	*Pn*	*Pd*	*Sc*	*Sl*	*Sd*	*Sle*	*Sq*	*Sn*	*Sr*	*Tb*	*Tc*	*To*	*Tj*	*Tt*	*Uh*	*Us*
tRNAF	0	0	0	0	0	0	0	0	0	0	0	0	0	0	0	0	0	0	0	0	0	0	0	0	0	0	0	0	0	0	0	0	0	0	0	0	0
12S rRNA	0	0	0	0	0	0	0	0	0	0	0	0	0	0	0	0	0	0	0	0	0	0	0	0	0	0	0	0	0	0	0	0	0	0	0	0	0
tRNAV	0	0	0	0	0	0	0	0	0	0	0	0	0	0	0	0	0	0	0	9	0	0	0	0	0	0	0	0	0	0	0	0	0	0	0	0	0
16S rRNA	0	0	0	0	0	0	0	0	0	0	0	0	0	0	0	0	0	0	0	0	0	5	0	0	0	0	0	0	0	0	-1	-1	0	0	0	0	0
tRNAL	0	0	0	0	0	0	0	0	0	0	0	0	0	0	0	0	0	0	0	0	0	0	0	0	0	0	0	0	0	0	-1	0	0	0	0	0	0
ND1	5	5	5	6	6	6	5	6	0	5	0	6	6	6	5	5	5	5	4	6	6	6	5	5	7	4	4	4	4	4	3	2	2	5	5	6	6
tRNAI	-1	-1	-1	-3	-1	-1	-1	-1	-1	-1	5	0	-1	-1	-1	-1	-1	-1	-1	-1	1	-1	-1	1	-1	-1	-1	-1	-1	-1	-1	-1	-1	-1	-1	1	-1
tRNAQ	-1	-1	-1	-1	-1	-1	-1	-1	-1	-1	-1	-1	-1	-1	-1	-1	-1	-1	-1	-1	-1	-1	-1	-1	-1	-1	-1	-1	-1	-1	-2	-1	-1	-1	-1	-1	-1
tRNAM	0	0	0	0	0	0	0	0	0	0	-1	0	0	0	0	0	0	0	0	0	0	0	0	0	0	0	0	0	0	0	-1	0	0	0	0	0	0
ND2	0	0	0	0	0	0	0	0	5	0	0	0	0	0	0	0	0	0	0	0	0	0	-1	0	-3	0	0	0	0	0	0	0	1	0	0	0	0
tRNAW	1	1	1	1	1	1	1	2	0	1	0	1	1	1	1	1	1	1	1	1	1	1	1	1	1	1	1	1	1	1	0	1	1	1	1	1	1
tRNAA	1	1	1	1	1	1	1	1	1	1	1	2	1	1	1	1	1	1	1	1	1	1	1	1	1	1	1	1	1	1	0	1	1	1	1	1	1

tRNAN	*38*	*38*	*38*	*37*	*38*	*37*	*35*	*38*	*38*	*39*	*38*	*37*	*37*	*37*	*38*	*38*	*38*	*37*	*39*	*37*	*37*	*36*	*36*	*37*	*37*	*34*	*34*	*34*	*34*	*34*	*41*	*42*	*41*	*38*	*38*	*36*	*34*
tRNAC	*0*	*0*	*0*	*0*	*0*	*0*	*-2*	*0*	*0*	*0*	*0*	*0*	*0*	*0*	*0*	*0*	*0*	*0*	*0*	*0*	*0*	*-2*	*0*	*0*	*0*	*0*	*0*	*0*	*0*	*0*	*-1*	*0*	*0*	*0*	*0*	*0*	*-2*
tRNAY	*1*	*1*	*1*	*1*	*1*	*1*	*1*	*0*	*1*	*1*	*1*	*1*	*1*	*1*	*1*	*1*	*1*	*1*	*1*	*7*	*1*	*1*	*1*	*1*	*1*	*1*	*1*	*1*	*1*	*1*	*1*	*1*	*1*	*1*	*1*	*1*	*1*
CO1	*0*	*0*	*0*	*0*	*0*	*0*	*0*	*0*	*0*	*0*	*0*	*0*	*0*	*0*	*0*	*0*	*0*	*0*	*0*	*0*	*0*	*0*	*0*	*0*	*0*	*0*	*0*	*0*	*0*	*0*	*-1*	*0*	*0*	*0*	*0*	*0*	*0*
tRNAS	*3*	*3*	*3*	*3*	*3*	*3*	*3*	*3*	*3*	*3*	*3*	*3*	*3*	*3*	*3*	*3*	*3*	*3*	*3*	*3*	*3*	*3*	*3*	*3*	*3*	*4*	*4*	*4*	*4*	*4*	*2*	*3*	*3*	*3*	*3*	*3*	*3*
tRNAD	*7*	*7*	*7*	*6*	*6*	*6*	*6*	*7*	*7*	*7*	*7*	*6*	*6*	*6*	*7*	*7*	*7*	*7*	*8*	*7*	*6*	*7*	*7*	*6*	*6*	*8*	*8*	*8*	*8*	*8*	*7*	*8*	*8*	*7*	*7*	*7*	*7*
CO2	*0*	*0*	*0*	*0*	*0*	*0*	*0*	*0*	*0*	*0*	*0*	*0*	*0*	*0*	*0*	*0*	*0*	*0*	*0*	*0*	*0*	*0*	*0*	*0*	*0*	*0*	*0*	*0*	*0*	*0*	*0*	*0*	*0*	*0*	*0*	*0*	*0*
tRNAK	*1*	*1*	*1*	*1*	*1*	*1*	*1*	*1*	*1*	*1*	*1*	*1*	*1*	*1*	*1*	*1*	*1*	*1*	*1*	*1*	*1*	*1*	*1*	*1*	*1*	*1*	*1*	*1*	*1*	*1*	*0*	*1*	*1*	*1*	*1*	*1*	*1*
ATP8	*-7*	*-7*	*-10*	*-4*	*-4*	*-4*	*-8*	*-10*	*-7*	*-10*	*-10*	*-4*	*-4*	*-4*	*-7*	*-7*	*-7*	*-7*	*-10*	*-4*	*-4*	*-10*	*-7*	*-7*	*-10*	*-10*	*-10*	*-10*	*-10*	*-10*	*-10*	*-10*	*-10*	*-7*	*-7*	*-4*	*-10*
ATP6	*-1*	*-1*	*-1*	*-1*	*-1*	*-1*	*-1*	*0*	*-1*	*-1*	*0*	*-1*	*-1*	*-1*	*-1*	*-1*	*-1*	*-1*	*-1*	*-1*	*-1*	*-1*	*-1*	*-1*	*-1*	*-1*	*-1*	*-1*	*-1*	*-1*	*-1*	*-1*	*-1*	*-1*	*-1*	*-1*	*-1*
CO3	*0*	*0*	*0*	*0*	*0*	*0*	*0*	*0*	*0*	*0*	*0*	*0*	*0*	*0*	*0*	*0*	*0*	*0*	*0*	*0*	*0*	*0*	*-1*	*0*	*0*	*0*	*0*	*0*	*0*	*0*	*0*	*0*	*1*	*0*	*1*	*0*	*0*
tRNAG	*0*	*0*	*0*	*0*	*0*	*0*	*0*	*0*	*0*	*0*	*0*	*0*	*0*	*0*	*0*	*0*	*0*	*0*	*0*	*0*	*0*	*0*	*0*	*0*	*0*	*0*	*0*	*0*	*0*	*0*	*-1*	*0*	*0*	*0*	*0*	*0*	*0*
ND3	*0*	*0*	*0*	*0*	*0*	*0*	*0*	*0*	*0*	*0*	*0*	*0*	*0*	*0*	*0*	*0*	*0*	*0*	*0*	*0*	*0*	*0*	*-2*	*0*	*0*	*0*	*0*	*0*	*0*	*0*	*0*	*0*	*0*	*0*	*0*	*0*	*0*
tRNAR	*1*	*1*	*1*	*1*	*1*	*1*	*1*	*1*	*1*	*1*	*1*	*1*	*1*	*1*	*1*	*1*	*1*	*1*	*0*	*1*	*1*	*2*	*1*	*1*	*1*	*0*	*0*	*0*	*0*	*0*	*6*	*0*	*0*	*1*	*1*	*1*	*2*
ND4L	*-7*	*-7*	*-7*	*-7*	*-7*	*-7*	*-7*	*-7*	*-7*	*-7*	*-7*	*-7*	*-7*	*-7*	*-7*	*-7*	*-7*	*-7*	*-7*	*-7*	*-7*	*-7*	*-7*	*-7*	*-7*	*-7*	*-7*	*-7*	*-7*	*-7*	*-7*	*-7*	*-7*	*-7*	*-7*	*-7*	*-7*
ND4	*0*	*1*	*1*	*0*	*0*	*0*	*0*	*0*	*0*	*0*	*0*	*0*	*0*	*0*	*0*	*0*	*0*	*0*	*0*	*0*	*0*	*0*	*0*	*0*	*0*	*0*	*0*	*0*	*0*	*0*	*0*	*0*	*0*	*0*	*0*	*0*	*0*
tRNAH	*0*	*0*	*0*	*0*	*0*	*0*	*1*	*0*	*0*	*0*	*0*	*0*	*0*	*0*	*0*	*0*	*0*	*0*	*0*	*0*	*0*	*1*	*0*	*0*	*0*	*0*	*-1*	*-1*	*0*	*0*	*-1*	*0*	*0*	*0*	*0*	*0*	*1*
tRNAS	*5*	*4*	*4*	*4*	*4*	*4*	*4*	*4*	*4*	*4*	*4*	*5*	*5*	*5*	*5*	*5*	*5*	*5*	*4*	*4*	*4*	*5*	*4*	*4*	*4*	*4*	*3*	*3*	*4*	*4*	*3*	*2*	*4*	*4*	*4*	*4*	*4*
tRNAL	*0*	*0*	*0*	*0*	*0*	*0*	*0*	*0*	*0*	*0*	*0*	*0*	*0*	*0*	*0*	*0*	*0*	*0*	*0*	*0*	*0*	*0*	*0*	*0*	*0*	*0*	*0*	*0*	*0*	*0*	*-1*	*0*	*0*	*0*	*0*	*0*	*0*

ND5	-4	-4	-4	-4	-4	-4	-4	-4	-4	-4	-4	-4	-4	-4	-4	-4	-4	-4	-4	-4	-4	-4	-4	-4	-4	-4	-4	-4	-4	-4	-3	-4	-4	-4	-4	-4	-4
ND6	0	0	0	0	0	0	0	0	0	0	0	0	0	0	0	0	0	0	1	0	0	0	0	1	0	1	1	1	1	1	0	0	0	0	0	0	0
tRNAE	4	4	4	5	5	5	4	4	4	4	4	4	4	4	4	4	4	4	4	4	4	4	4	4	4	4	4	4	5	5	3	4	4	4	4	4	4
Cytb	0	0	0	0	0	0	0	0	0	0	0	0	0	0	0	0	0	0	0	0	0	0	0	0	0	0	0	0	0	0	0	0	0	0	0	0	0
tRNAT	-1	-1	-1	-1	-1	-1	-1	-1	-1	-1	-1	-1	-1	-1	-1	-1	-1	-1	-1	-1	-1	-1	-1	-1	-1	-1	-1	-1	-1	-1	-1	-1	-1	-1	-1	-1	-1
tRNAP	0	0	0	0	0	0	0	0	0	0	0	0	0	0	0	0	0	0	0	0	0	0	0	0	0	0	0	0	0	0	0	1	0	0	0	0	0
D loop	0	0	0	0	0	0	0	0	0	0	0	0	0	0	0	0	0	0	0	0	0	0	0	0	0	0	0	0	0	0	0	0	0	0	0	0	0

Table S4. Comparison of start and stop codon of PCGs within 37 species of Carangidae. *Dr=D. russelli, Ac= A. ciliaris, Ai= A. indica, Ad= A. djedaba, Ak= A. kleinii, Am= A. mate, Ca= C. armatus, Cb= C. bajad, Ce= C. equula, Cm=c. malabaricus, Cp= C. plagiotaenia, Ci= C. ignobilis, Cmel=C. melampygus, Ct= C. tille, Dmac = D. macerellus, Dm= D. macrosoma, Dmar= D. maruadsi,, Dt= D. tabl, Eb= E. bipinnulata, Gs= G. speciosus, Mc= M. cordyla, Pn= P. niger, Pd= P. dentex, Sc=S. crumenophthalmus, Sle= S. leptolepis, Sd= S. dumerili, Sl=S. lalandi, Sq=S. quinqueradiata, Sr= S. rivoliana, Sn= S. nigrofasciata, Tb=T. blochii, Tc=T. carolinus, To= T. ovatus, Tj= T. japonicas, Tt= T. trachurus, Uh= U. helvola, Us= U. secunda.*

Locus		ND1	ND2	CO1	CO2	ATP8	ATP6	CO3	ND3	ND4L	ND4	ND5	ND6	Cytb
Dr	Start	ATG	ATG	GTG	ATG	ATG	ATG	ATG	ATG	ATG	ATG	ATG	ATG	ATG
	Stop	TAA	T	TAA	T	TAG	TAA	TA	T	TAA	T	TAG	TAG	T
Ac	Start	ATG	ATG	GTG	ATG	ATG	ATG	ATG	ATG	ATG	ATG	ATG	ATG	ATG
	Stop	TAG	T	TAA	T	TAA	TAA	TA	T	TAA	T	TAA	TAG	T
Ai	Start	ATG	ATG	GTG	ATG	ATG	ATG	ATG	ATG	ATG	ATG	ATG	ATG	ATG
	Stop	TAG	T	TAA	T	TAA	TAA	TA	T	TAA	T	TAA	TAA	T
Ad	Start	ATG	ATG	GTG	ATG	ATG	ATA	ATA	ATG	ATG	ATG	ATG	ATG	ATG
	Stop	TAA	TAG	TAA	T	TAA	TAA	TAA	TAG	TAA	T	TAG	TAA	T
Ak	Start	ATG	ATG	GTG	ATG	ATG	ATA	ATG	ATG	ATG	ATG	ATG	ATG	ATG
	Stop	TAA	T	TAA	T	TAA	TAA	TA	T	TAA	T	TAA	TAG	T
Am	Start	ATG	ATG	GTG	ATG	ATG	ATA	ATG	ATG	ATG	ATG	ATG	ATG	ATG
	Stop	TAA	TAG	TAA	T	TAA	TAA	TA	TAG	TAA	T	TAA	TAG	T
Ca	Start	ATG	ATG	GTG	ATG	ATG	ATG	ATG	ATG	ATG	ATG	ATG	ATG	ATG
	Stop	TAG	T	TAA	T	TAA	TA	TA	T	TAA	T	TAA	TAA	T
Cb	Start	ATG	ATG	GTG	ATG	ATG	ATG	ATG	ATG	ATG	ATG	ATG	ATG	ATG
	Stop	TAG	T	TAA	T	TAA	TAA	TA	T	TAA	T	TAG	TAG	T
Ce	Start	ATG	ATG	GTG	ATG	ATG	GTG	ATG	ATG	ATG	ATG	ATG	ATG	ATG
	Stop	TAA	TAG	TAA	T	TAA	TAA	TA	T	TAA	T	TAG	TAA	T
Cm	Start	ATG	ATG	GTG	ATG	ATG	ATG	ATG	ATG	ATG	ATG	ATG	ATG	ATG
	Stop	TAG	T	TAA	T	TAA	TAA	TA	T	TAA	T	TAA	TAG	T
Cp	Start	ATG	ATG	GTG	ATG	ATG	ATG	ATG	ATG	ATG	ATG	ATG	ATG	ATG
	Stop	TAG	T	TAA	T	TAA	TAA	TA	T	TAA	T	TAG	TAG	T
Ci	Start	ATG	ATG	GTG	ATG	ATG	ATA	ATG	ATG	ATG	ATG	ATG	ATG	ATG
	Stop	TAA	T	TAA	T	TAA	TAA	TA	T	TAA	T	TAA	TAG	T

Cmel	Start	ATG	ATG	GTG	ATG	ATG	ATA	ATG	ATG	ATG	ATG	ATG	ATG	ATG
	Stop	TAA	T	TAA	T	TAA	TAA	TA	T	TAA	T	TAA	TAG	T
Ct	Start	ATG	ATG	GTG	ATG	ATG	ATA	ATG	ATG	ATG	ATG	ATG	ATG	ATG
	Stop	TAA	T	TAA	T	TAA	TAA	TA	T	TAA	T	TAA	TAG	T
Dm	Start	ATG	ATG	GTG	ATG	ATG	ATG	ATG	ATG	ATG	ATG	ATG	ATG	ATG
	Stop	TAA	T	TAA	T	TAG	TAA	TA	T	TAA	T	TAA	TAG	T
Dmac	Start	ATG	ATG	GTG	ATG	ATG	ATG	ATG	ATG	ATG	ATG	ATG	ATG	ATG
	Stop	TAA	T	TAA	T	TAG	TAA	TA	T	TAA	T	TAG	TAG	T
Dmar	Start	ATG	ATG	GTG	ATG	ATG	ATG	ATG	ATG	ATG	ATG	ATG	ATG	ATG
	Stop	TAA	T	TAA	T	TAG	TAA	TA	T	TAA	T	TAG	TAG	T
Dt	Start	ATG	ATG	GTG	ATG	ATG	ATG	ATG	ATG	ATG	ATG	ATG	ATG	ATG
	Stop	TAA	T	TAA	T	TAG	TAA	TA	T	TAA	T	TAA	TAA	T
Eb	Start	ATG	ATG	GTG	ATG	ATG	ATG	ATG	ATG	ATG	ATG	ATG	ATG	ATG
	Stop	TAA	TAA	TAA	T	TAA	TAA	TAA	TAG	TAA	T	TAG	TAA	T
Gs	Start	ATG	ATG	ATC	ATG	ATG	ATG	ATG	ATG	ATG	ATG	ATG	ATG	ATG
	Stop	TAA	T	TAA	T	TAA	TAA	TA	T	TAA	T	TAA	TAG	T
Mc	Start	ATG	ATG	GTG	ATG	ATG	ATA	ATG	ATG	ATG	ATG	ATG	ATG	ATG
	Stop	TAA	T	TAA	T	TAA	TAA	TA	T	TAA	T	TAA	TAG	T
Pn	Start	ATG	ATG	GTG	ATG	ATG	ATG	ATG	ATG	ATG	ATG	ATG	ATG	ATG
	Stop	TAA	T	TAA	T	TAA	TAA	TA	T	TAA	T	TAA	TAG	T
Pd	Start	ATG	ATG	GTG	ATG	ATG	ATG	ATG	ATG	ATG	ATG	ATG	ATG	ATG
	Stop	TAA	T	TAA	T	TAG	TAA	TAA	T	TAA	T	TAA	TAA	T
Sc	Start	ATG	ATG	GTG	ATG	ATG	ATG	ATG	ATG	ATG	ATG	ATG	ATG	ATG
	Stop	TAG	T	TAA	T	TAG	TAA	TA	T	TAA	T	TAA	TAA	T
Sle	Start	ATG	ATG	GTG	ATG	ATG	ATG	ATG	ATG	ATG	ATG	ATG	ATG	ATG
	Stop	TAA	TAG	TAA	T	TAA	TAA	TA	T	TAA	T	TAA	TAG	T
Sd	Start	ATG	ATG	GTG	ATG	ATG	ATG	ATG	ATG	ATG	ATG	ATG	ATG	ATG
	Stop	TAA	TA	TAA	T	TAA	TAA	TA	T	TAA	T	TAA	TAG	T
Sl	Start	ATG	ATG	GTG	ATG	ATG	ATG	ATG	ATG	ATG	ATG	ATG	ATG	ATG
	Stop	TAA	TA	TAA	T	TAA	TAA	TA	T	TAA	T	TAA	TAG	T
Sq	Start	ATG	ATG	GTG	ATG	ATG	ATG	ATG	ATG	ATG	ATG	ATG	ATG	ATG
	stop	TAA	TA	TAA	T	TAA	TAA	TA	T	TAA	T	TAA	TAG	T

Sr	Start	ATG	ATG	GTG	ATG	ATG	ATG	ATG	ATG	ATG	ATG	ATG	ATG	ATG
	Stop	TAA	TAA	TAA	T	TAA	TAA	TAA	TAG	TAA	T	TAA	TAG	T
Sn	Start	ATG	ATG	GTG	ATG	ATG	ATG	ATG	ATG	ATG	ATG	ATG	ATG	ATG
	Stop	TAA	TA	TAA	T	TAA	TAA	TA	T	TAA	T	TAA	TAG	T
Tb	Start	ATG	ATG	GTG	ATG	ATG	ATG	ATG	ATG	ATG	ATG	ATG	GTG	ATG
	Stop	TAA	TA	TAA	T	TAA	TAA	TA	T	TAG	T	TA	TAA	T
Tc	Start	ATG	ATG	GTG	ATG	ATG	ATG	ATG	ATG	ATG	ATG	ATG	GTG	ATG
	Stop	TAA	TA	TAA	T	TAA	TAA	TA	T	TAG	T	TAA	TAA	T
To	Start	ATG	ATG	GTG	ATG	ATG	ATG	ATG	ATG	ATG	ATG	ATG	GTG	ATG
	Stop	TAA	TA	TAA	T	TAA	TAA	TA	T	TAG	T	TAA	TAA	T
Tj	Start	ATG	ATG	GTG	ATG	ATG	ATG	ATG	ATG	ATG	ATG	ATG	ATG	ATG
	Stop	TAA	T	TAA	T	TAG	TAA	TA	T	TAA	T	TAA	TAA	T
Tt	Start	ATG	ATG	GTG	ATG	ATG	ATG	ATG	ATG	ATA	ATG	ATG	ATG	ATG
	Stop	TAA	T	TAA	T	TAA	TAA	T	T	TAA	T	TAA	TAA	T
Uh	Start	ATG	ATG	GTG	ATG	ATG	ATA	ATG	ATG	ATG	ATG	ATG	ATG	ATG
	Stop	TAA	T	TAA	T	TAA	TAA	TA	T	TAA	T	TAA	TAG	T
Us	Start	ATG	ATG	GTG	ATG	ATG	ATG	ATG	ATG	ATG	ATG	ATG	ATG	ATG
	Stop	TAA	TA	TAA	T	TAA	TAA	TA	T	TAA	T	TAA	TAG	T

Table S5. The Ka/Ks value of 13 PCGs in 37 complete mitogenomes of Carangidae species. *Dr=D. russelli, Ac= A. ciliaris, Ai= A. indica, Ad= A. djedaba, Ak= A. kleinii, Am= A. mate, Ca= C. armatus,Cb= C. bajad, Ce= C. equula, Cm= C. malabaricus, Cp= C. plagiotaenia, Ci= C. ignobilis, Cmel=C. melampygus, Ct= C. tille, Dmac = D. macerellus, Dm= D. macrosoma, Dmar= D. maruadsi,, Dt= D. tabl, Eb= E. bipinnulata, Gs= G. speciosus, Mc= M. cordyla, Pn= P. niger, Pd= P. dentex, Sc=S. crumenophthalmus, Sle= S. leptolepis, Sd= S. dumerili, Sl=S. lalandi, Sq=S. quinqueradiata, Sr= S. rivoliana, Sn= S. nigrofasciata, Tb=T. blochii, Tc=T. carolinus, To= T. ovatus, Tj= T. japonicas, Tt= T. trachurus, Uh= U. helvola, Us= U. secunda.*

	ND1	ND2	CO1	CO2	ATP8	ATP6	CO3	ND3	ND4L	ND4	ND5	ND6	Cyt-B
Ac	0.013	0.036	0.006	0.012	0.107	0.023	0.010	0.026	0.016	0.019	0.025	0.053	0.012
Ai	0.0110	0.0485	0.0043	0.0094	0.0718	0.0268	0.009	0.0474	0.0343	0.0218	0.0243	0.0387	0.019
Ad	0.011	0.035	0.010	0.013	0.109	0.839	0.012	0.035	0.018	0.023	0.036	0.030	0.007
Ak	0.014	0.035	0.011	0.012	0.169	0.890	0.010	0.039	0.012	0.019	0.045	0.038	0.009
Am	0.014	0.026	0.012	0.011	0.107	1.135	0.011	0.012	0.021	0.025	0.031	0.049	0.013
Ca	0.009	0.050	0.006	0.009	0.052	0.022	0.007	0.020	0.008	0.011	0.030	0.044	0.016
Cb	0.009	0.041	0.007	0.007	0.047	0.019	0.015	0.015	0.017	0.017	0.017	0.027	0.021
Ce	0.017	0.079	0.008	0.079	0.087	0.022	0.012	0.040	0.063	0.022	0.032	0.317	0.011
Cm	0.010	0.037	0.005	0.019	0.071	0.007	0.016	0.020	0.023	0.024	0.027	0.058	0.016
Cp	0.012	0.049	0.007	0.009	0.057	0.020	0.007	0.019	0.029	0.015	0.031	0.047	0.016
Ci	0.011	0.038	0.009	0.038	0.087	1.202	0.011	0.031	0.013	0.012	0.028	0.045	0.012
Cmel	0.011	0.039	0.010	0.019	0.105	1.478	0.009	0.028	0.015	0.013	0.035	0.045	0.013
Ct	0.012	0.039	0.010	0.019	0.009	1.512	0.009	0.028	0.157	0.012	0.038	0.440	0.013
Dmac	0.008	0.024	0.011	0.019	0.050	0	0.004	0.036	0	0.015	0.007	0.035	0
Dm	0.017	0.025	0.008	0.010	0	0	0.003	0.008	0.016	0.012	0.011	0.040	0.002
Dmar	0.014	0.119	0.054	0.037	0.307	0	0.040	0	0	0.014	0.029	0.110	0
Dt	0.006	0.020	0.007	0.003	0.044	0.008	0.006	0.015	0.008	0.009	0.010	0.031	0
Eb	0.018	0.053	0.012	0.013	0.059	0.025	0.050	0.032	0.033	0.018	0.040	0.035	0.010
Gs	0.012	0.047	1.363	0.017	0.129	1.295	0.014	0.024	0.014	0.016	0.029	0.044	0.020
Mc	0.011	0.037	0.009	0.020	0.084	1.031	0.017	0.030	0.006	0.0174	0.0315	0.052	0.015

Pn	0.007	0.041	0.004	0.004	0.109	0.021	0.011	0.024	0.010	0.013	0.030	0.077	0.012
Pd	0.019	0.049	0.005	0.006	0.035	0.014	0.014	0.046	0.051	0.018	0.029	0.176	0.024
Sc	0.005	0.041	0.027	0.011	0.092	0.021	0.012	0.027	0.023	0.017	0.019	0.066	0.006
Sle	0.012	0.037	0.020	0.017	0.080	0.954	0.007	0.021	0.026	0.016	0.028	0.055	0.012
Sd	0.018	0.044	0.011	0.017	0.028	0.019	0.040	0.040	0.018	0.026	0.044	0.041	0.034
Sl	0.0159	0.05818	0.009	0.014	0.00	0.018	0.050	0.055	0.024	0.031	0.045	0.054	0.031
Sq	0.014	0.049	0.009	0.015	0.00	0.021	0.057	0.072	0.025	0.024	0.035	0.053	0.038
Sr	0.019	0.054	0.012	0.0167	0.018	0.017	0.046	0.051	0.030	0.026	0.046	0.040	0.037
Sn	0.024	0.069	0.014	0.017	0.040	0.021	0.040	0.082	0.036	0.040	0.032	0.696	0.029
Tb	0.027	0.058	0.014	0.016	0.087	0.017	0.039	0.051	0.047	0.106	0.060	0.057	0.032
Tc	0.024	0.060	0.014	0.168	0.115	0.025	0.035	0.029	0.046	0.103	0.049	0.059	0.037
To	0.023	0.050	0.014	0.015	0.115	0.028	0.035	0.035	0.036	0.117	0.049	0.061	0.029
Tj	0.007	0.023	0.009	0.009	0.056	0.008	0.005	0.022	0.008	0.020	0.016	0.043	0.004
Tt	0.009	0.023	0.009	0.010	0.059	0.007	0.003	0.032	0.009	0.017	0.018	0.040	0.005
Uh	0.013	0.035	0.004	0.005	0.080	1.080	0.013	0.029	0.009	0.014	0.030	0.068	0.016
Us	0.013	0.039	0.004	0.005	0.080	0.030	0.013	0.027	0.009	0.014	0.031	0.065	0.016

Table S6. The RSCU value of the complete PCGs in the mitogenome of 37 Carangidae species

D. russelli							
Codon	RCSU	Codon	RCSU	Codon	RCSU	Codon	RCSU
UUU(F)	0.71	UCU(S)	0.66	UAU(Y)	0.5	UGU(C)	0.43
UUC(F)	1.29	UCC(S)	2.13	UAC(Y)	1.5	UGC(C)	1.57
UUA(L)	0.48	UCA(S)	1.44	UAA(*)	1.6	UGA(W)	1.81
UUG(L)	0.06	UCG(S)	0.38	UAG(*)	0.4	UGG(W)	0.19
CUU(L)	1.47	CCU(P)	0.9	CAU(H)	0.51	CGU(R)	0.65
CUC(L)	1.79	CCC(P)	1.87	CAC(H)	1.49	CGC(R)	1.16
CUA(L)	1.79	CCA(P)	0.9	CAA(Q)	1.46	CGA(R)	1.86
CUG(L)	0.41	CCG(P)	0.32	CAG(Q)	0.54	CGG(R)	0.33
AUU(I)	0.97	ACU(T)	0.56	AAU(N)	0.45	AGU(S)	0.13
AUC(I)	1.03	ACC(T)	1.83	AAC(N)	1.55	AGC(S)	1.27
AUA(M)	0.99	ACA(T)	1.37	AAA(K)	1.78	AGA(*)	1.4
AUG(M)	1.01	ACG(T)	0.24	AAG(K)	0.22	AGG(*)	0.6
GUU(V)	1.1	GCU(A)	0.68	GAU(D)	0.39	GGU(G)	0.58
GUC(V)	1.43	GCC(A)	1.93	GAC(D)	1.61	GGC(G)	1.55
GUA(V)	1.3	GCA(A)	1.24	GAA(E)	1.67	GGA(G)	1.31
GUG(V)	0.17	GCG(A)	0.15	GAG(E)	0.33	GGG(G)	0.56
A.ciliaris							
Codon	RCSU	Codon	RCSU	Codon	RCSU	Codon	RCSU
UUU(F)	0.78	UCU(S)	0.76	UAU(Y)	0.87	UGU(C)	0.41
UUC(F)	1.22	UCC(S)	1.8	UAC(Y)	1.13	UGC(C)	1.59
UUA(L)	0.83	UCA(S)	1.67	UAA(*)	1.63	UGA(W)	1.78
UUG(L)	0.09	UCG(S)	0.22	UAG(*)	0.74	UGG(W)	0.22
CUU(L)	1.63	CCU(P)	1.15	CAU(H)	0.74	CGU(R)	0.58
CUC(L)	1.59	CCC(P)	1.44	CAC(H)	1.26	CGC(R)	0.95
CUA(L)	1.6	CCA(P)	1.27	CAA(Q)	1.85	CGA(R)	2.21
CUG(L)	0.26	CCG(P)	0.14	CAG(Q)	0.15	CGG(R)	0.26
AUU(I)	1.08	ACU(T)	0.9	AAU(N)	0.61	AGU(S)	0.37
AUC(I)	0.92	ACC(T)	1.44	AAC(N)	1.39	AGC(S)	1.18
AUA(M)	1.39	ACA(T)	1.52	AAA(K)	1.72	AGA(*)	1.04
AUG(M)	0.61	ACG(T)	0.14	AAG(K)	0.28	AGG(*)	0.59
GUU(V)	1.21	GCU(A)	0.97	GAU(D)	0.54	GGU(G)	0.66
GUC(V)	1.4	GCC(A)	1.61	GAC(D)	1.46	GGC(G)	1.4
GUA(V)	1.19	GCA(A)	1.3	GAA(E)	1.72	GGA(G)	1.47
GUG(V)	0.21	GCG(A)	0.13	GAG(E)	0.28	GGG(G)	0.47
A.indica							
Codon	RSCU	Codon	RSCU	Codon	RSCU	Codon	RSCU
UUU(F)	0.69	UCU(S)	1.19	UAU(Y)	0.77	UGU(C)	0.4
UUC(F)	1.31	UCC(S)	1.78	UAC(Y)	1.23	UGC(C)	1.6
UUA(L)	0.71	UCA(S)	1.58	UAA(*)	0	UGA(W)	1.64
UUG(L)	0.15	UCG(S)	0.18	UAG(*)	0	UGG(W)	0.36
CUU(L)	1.31	CCU(P)	1.22	CAU(H)	0.74	CGU(R)	0.35
CUC(L)	1.77	CCC(P)	1.67	CAC(H)	1.26	CGC(R)	1.06
CUA(L)	1.68	CCA(P)	0.95	CAA(Q)	1.78	CGA(R)	2.18
CUG(L)	0.38	CCG(P)	0.16	CAG(Q)	0.22	CGG(R)	0.41
AUU(I)	0.94	ACU(T)	0.71	AAU(N)	0.47	AGU(S)	0.18
AUC(I)	1.06	ACC(T)	1.7	AAC(N)	1.53	AGC(S)	1.09
AUA(M)	1.26	ACA(T)	1.46	AAA(K)	1.81	AGA(*)	0
AUG(M)	0.74	ACG(T)	0.13	AAG(K)	0.19	AGG(*)	0
GUU(V)	1.26	GCU(A)	0.8	GAU(D)	0.45	GGU(G)	0.5

GUC(V)	1.24	GCC(A)	1.72	GAC(D)	1.55	GGC(G)	1.34
GUA(V)	1.09	GCA(A)	1.3	GAA(E)	1.7	GGA(G)	1.34
GUG(V)	0.41	GCG(A)	0.18	GAG(E)	0.3	GGG(G)	0.81
A.djedaba							
Codon	RCSU	Codon	RCSU	Codon	RCSU	Codon	RCSU
UUU(F)	0.64	UCU(S)	0.93	UAU(Y)	0.7	UGU(C)	0.28
UUC(F)	1.36	UCC(S)	1.8	UAC(Y)	1.3	UGC(C)	1.72
UUA(L)	0.73	UCA(S)	1.62	UAA(*)	2.1	UGA(W)	1.8
UUG(L)	0.15	UCG(S)	0.22	UAG(*)	0	UGG(W)	0.2
CUU(L)	1.75	CCU(P)	1.21	CAU(H)	0.48	CGU(R)	0.67
CUC(L)	1.42	CCC(P)	1.68	CAC(H)	1.52	CGC(R)	0.87
CUA(L)	1.52	CCA(P)	0.94	CAA(Q)	1.59	CGA(R)	2.26
CUG(L)	0.43	CCG(P)	0.17	CAG(Q)	0.41	CGG(R)	0.21
AUU(I)	1.08	ACU(T)	0.77	AAU(N)	0.61	AGU(S)	0.22
AUC(I)	0.92	ACC(T)	1.59	AAC(N)	1.39	AGC(S)	1.2
AUA(M)	1.11	ACA(T)	1.36	AAA(K)	1.68	AGA(*)	1.14
AUG(M)	0.89	ACG(T)	0.28	AAG(K)	0.32	AGG(*)	0.76
GUU(V)	1.11	GCU(A)	0.68	GAU(D)	0.67	GGU(G)	0.64
GUC(V)	1.19	GCC(A)	2.01	GAC(D)	1.33	GGC(G)	1.42
GUA(V)	1.56	GCA(A)	1.23	GAA(E)	1.66	GGA(G)	1.4
GUG(V)	0.14	GCG(A)	0.07	GAG(E)	0.34	GGG(G)	0.55
A.kleinii							
Codon	RCSU	Codon	RCSU	Codon	RCSU	Codon	RCSU
UUU(F)	0.86	UCU(S)	0.87	UAU(Y)	0.83	UGU(C)	0.47
UUC(F)	1.14	UCC(S)	2.08	UAC(Y)	1.17	UGC(C)	1.53
UUA(L)	0.79	UCA(S)	1.47	UAA(*)	2	UGA(W)	1.75
UUG(L)	0.16	UCG(S)	0.37	UAG(*)	1.22	UGG(W)	0.25
CUU(L)	1.71	CCU(P)	1.06	CAU(H)	0.63	CGU(R)	0.75
CUC(L)	1.22	CCC(P)	1.68	CAC(H)	1.37	CGC(R)	0.96
CUA(L)	1.57	CCA(P)	1.03	CAA(Q)	1.5	CGA(R)	1.92
CUG(L)	0.54	CCG(P)	0.24	CAG(Q)	0.5	CGG(R)	0.37
AUU(I)	1.23	ACU(T)	0.7	AAU(N)	0.73	AGU(S)	0.31
AUC(I)	0.77	ACC(T)	1.55	AAC(N)	1.27	AGC(S)	0.89
AUA(M)	1.14	ACA(T)	1.42	AAA(K)	1.8	AGA(*)	0.52
AUG(M)	0.86	ACG(T)	0.33	AAG(K)	0.2	AGG(*)	0.26
GUU(V)	1.47	GCU(A)	0.9	GAU(D)	0.88	GGU(G)	0.61
GUC(V)	0.92	GCC(A)	1.81	GAC(D)	1.12	GGC(G)	1.38
GUA(V)	1.36	GCA(A)	1.15	GAA(E)	1.47	GGA(G)	1.52
GUG(V)	0.24	GCG(A)	0.14	GAG(E)	0.53	GGG(G)	0.49
A.mate							
Codon	RSCU	Codon	RSCU	Codon	RSCU	Codon	RSCU
UUU(F)	1.02	UCU(S)	1.45	UAU(Y)	0.74	UGU(C)	0.54
UUC(F)	0.98	UCC(S)	1.53	UAC(Y)	1.26	UGC(C)	1.46
UUA(L)	1.16	UCA(S)	1.45	UAA(*)	0	UGA(W)	1.77
UUG(L)	0.16	UCG(S)	0.26	UAG(*)	0	UGG(W)	0.23
CUU(L)	1.8	CCU(P)	1.3	CAU(H)	0.58	CGU(R)	0.62
CUC(L)	1.13	CCC(P)	1.43	CAC(H)	1.42	CGC(R)	0.82
CUA(L)	1.39	CCA(P)	1.09	CAA(Q)	1.71	CGA(R)	2.26
CUG(L)	0.36	CCG(P)	0.18	CAG(Q)	0.29	CGG(R)	0.31
AUU(I)	1.27	ACU(T)	0.94	AAU(N)	0.77	AGU(S)	0.31
AUC(I)	0.73	ACC(T)	1.42	AAC(N)	1.23	AGC(S)	0.99
AUA(M)	1.06	ACA(T)	1.46	AAA(K)	1.81	AGA(*)	0
AUG(M)	0.94	ACG(T)	0.19	AAG(K)	0.19	AGG(*)	0

GUU(V)	1.3	GCU(A)	1.17	GAU(D)	0.8	GGU(G)	0.76
GUC(V)	1.14	GCC(A)	1.34	GAC(D)	1.2	GGC(G)	1.25
GUA(V)	1.32	GCA(A)	1.32	GAA(E)	1.75	GGA(G)	1.43
GUG(V)	0.24	GCG(A)	0.17	GAG(E)	0.25	GGG(G)	0.56
C.armatus							
Codon	RCSU	Codon	RCSU	Codon	RCSU	Codon	RCSU
UUU(F)	0.83	UCU(S)	1.08	UAU(Y)	0.61	UGU(C)	0.47
UUC(F)	1.17	UCC(S)	1.82	UAC(Y)	1.39	UGC(C)	1.53
UUA(L)	0.76	UCA(S)	1.44	UAA(*)	1.73	UGA(W)	1.8
UUG(L)	0.07	UCG(S)	0.18	UAG(*)	0.67	UGG(W)	0.2
CUU(L)	1.65	CCU(P)	1.02	CAU(H)	0.49	CGU(R)	0.37
CUC(L)	1.65	CCC(P)	1.85	CAC(H)	1.51	CGC(R)	1.16
CUA(L)	1.59	CCA(P)	0.98	CAA(Q)	1.73	CGA(R)	2.11
CUG(L)	0.29	CCG(P)	0.15	CAG(Q)	0.27	CGG(R)	0.37
AUU(I)	1.09	ACU(T)	0.74	AAU(N)	0.65	AGU(S)	0.18
AUC(I)	0.91	ACC(T)	1.58	AAC(N)	1.35	AGC(S)	1.31
AUA(M)	1.35	ACA(T)	1.56	AAA(K)	1.86	AGA(*)	0.93
AUG(M)	0.65	ACG(T)	0.12	AAG(K)	0.14	AGG(*)	0.67
GUU(V)	1.23	GCU(A)	0.87	GAU(D)	0.49	GGU(G)	0.52
GUC(V)	1.19	GCC(A)	1.82	GAC(D)	1.51	GGC(G)	1.59
GUA(V)	1.41	GCA(A)	1.22	GAA(E)	1.68	GGA(G)	1.37
GUG(V)	0.18	GCG(A)	0.09	GAG(E)	0.32	GGG(G)	0.52
C. bajad							
Codon	RSCU	Codon	RSCU	Codon	RSCU	Codon	RSCU
UUU(F)	0.89	UCU(S)	1.11	UAU(Y)	0.67	UGU(C)	0.4
UUC(F)	1.11	UCC(S)	1.86	UAC(Y)	1.33	UGC(C)	1.6
UUA(L)	0.78	UCA(S)	1.73	UAA(*)	0	UGA(W)	1.8
UUG(L)	0.1	UCG(S)	0.05	UAG(*)	0	UGG(W)	0.2
CUU(L)	1.38	CCU(P)	1.12	CAU(H)	0.58	CGU(R)	0.62
CUC(L)	1.69	CCC(P)	1.77	CAC(H)	1.42	CGC(R)	1.03
CUA(L)	1.74	CCA(P)	1.01	CAA(Q)	1.79	CGA(R)	2
CUG(L)	0.31	CCG(P)	0.11	CAG(Q)	0.21	CGG(R)	0.36
AUU(I)	1.19	ACU(T)	0.75	AAU(N)	0.42	AGU(S)	0.18
AUC(I)	0.81	ACC(T)	1.59	AAC(N)	1.58	AGC(S)	1.06
AUA(M)	1.22	ACA(T)	1.54	AAA(K)	1.95	AGA(*)	0
AUG(M)	0.78	ACG(T)	0.12	AAG(K)	0.05	AGG(*)	0
GUU(V)	1.27	GCU(A)	0.86	GAU(D)	0.52	GGU(G)	0.38
GUC(V)	1.33	GCC(A)	1.74	GAC(D)	1.48	GGC(G)	1.54
GUA(V)	1.1	GCA(A)	1.26	GAA(E)	1.67	GGA(G)	1.48
GUG(V)	0.3	GCG(A)	0.14	GAG(E)	0.33	GGG(G)	0.61
C.equula							
Codon	RCSU	Codon	RCSU	Codon	RCSU	Codon	RCSU
UUU(F)	0.76	UCU(S)	0.79	UAU(Y)	0.67	UGU(C)	0.43
UUC(F)	1.24	UCC(S)	1.74	UAC(Y)	1.33	UGC(C)	1.57
UUA(L)	0.59	UCA(S)	1.56	UAA(*)	0.44	UGA(W)	1.56
UUG(L)	0.24	UCG(S)	0.4	UAG(*)	1.33	UGG(W)	0.44
CUU(L)	1.4	CCU(P)	0.52	CAU(H)	0.38	CGU(R)	0.47
CUC(L)	1.68	CCC(P)	2.26	CAC(H)	1.62	CGC(R)	1.26
CUA(L)	1.36	CCA(P)	0.87	CAA(Q)	1.5	CGA(R)	1.72
CUG(L)	0.73	CCG(P)	0.35	CAG(Q)	0.5	CGG(R)	0.56
AUU(I)	1.05	ACU(T)	0.61	AAU(N)	0.45	AGU(S)	0.12
AUC(I)	0.95	ACC(T)	1.72	AAC(N)	1.55	AGC(S)	1.39
AUA(M)	0.99	ACA(T)	1.19	AAA(K)	1.29	AGA(*)	0.89

AUG(M)	1.01	ACG(T)	0.48	AAG(K)	0.71	AGG(*)	1.33
GUU(V)	0.99	GCU(A)	0.65	GAU(D)	0.45	GGU(G)	0.49
GUC(V)	1.53	GCC(A)	1.98	GAC(D)	1.55	GGC(G)	1.52
GUA(V)	1.1	GCA(A)	1.03	GAA(E)	1.4	GGA(G)	1.32
GUG(V)	0.38	GCG(A)	0.34	GAG(E)	0.6	GGG(G)	0.67
C.malabaricus							
Codon	RCSU	Codon	RCSU	Codon	RCSU	Codon	RCSU
UUU(F)	0.87	UCU(S)	1.35	UAU(Y)	0.72	UGU(C)	0.63
UUC(F)	1.13	UCC(S)	1.88	UAC(Y)	1.28	UGC(C)	1.38
UUA(L)	1.11	UCA(S)	1.5	UAA(*)	1.91	UGA(W)	1.47
UUG(L)	0.49	UCG(S)	0.75	UAG(*)	1.76	UGG(W)	0.53
CUU(L)	1.21	CCU(P)	0.99	CAU(H)	0.85	CGU(R)	0.55
CUC(L)	1.16	CCC(P)	1.71	CAC(H)	1.15	CGC(R)	1.1
CUA(L)	1.51	CCA(P)	0.97	CAA(Q)	1.2	CGA(R)	1.93
CUG(L)	0.52	CCG(P)	0.33	CAG(Q)	0.8	CGG(R)	0.41
AUU(I)	1.08	ACU(T)	0.82	AAU(N)	0.76	AGU(S)	0.09
AUC(I)	0.92	ACC(T)	1.5	AAC(N)	1.24	AGC(S)	0.44
AUA(M)	1.19	ACA(T)	1.29	AAA(K)	1.54	AGA(*)	0.24
AUG(M)	0.81	ACG(T)	0.38	AAG(K)	0.46	AGG(*)	0.09
GUU(V)	1.24	GCU(A)	0.92	GAU(D)	0.64	GGU(G)	0.51
GUC(V)	1.24	GCC(A)	1.81	GAC(D)	1.36	GGC(G)	1.19
GUA(V)	1.21	GCA(A)	1.11	GAA(E)	1.36	GGA(G)	1.38
GUG(V)	0.32	GCG(A)	0.16	GAG(E)	0.64	GGG(G)	0.92
C. plagiotaenia							
Codon	RSCU	Codon	RSCU	Codon	RSCU	Codon	RSCU
UUU(F)	0.86	UCU(S)	1.42	UAU(Y)	0.83	UGU(C)	0.64
UUC(F)	1.14	UCC(S)	1.65	UAC(Y)	1.17	UGC(C)	1.36
UUA(L)	0.86	UCA(S)	1.5	UAA(*)	0	UGA(W)	1.8
UUG(L)	0.13	UCG(S)	0.15	UAG(*)	0	UGG(W)	0.2
CUU(L)	1.56	CCU(P)	0.93	CAU(H)	0.64	CGU(R)	0.62
CUC(L)	1.47	CCC(P)	1.67	CAC(H)	1.36	CGC(R)	0.82
CUA(L)	1.63	CCA(P)	1.23	CAA(Q)	1.81	CGA(R)	2.15
CUG(L)	0.34	CCG(P)	0.16	CAG(Q)	0.19	CGG(R)	0.41
AUU(I)	1.21	ACU(T)	0.92	AAU(N)	0.58	AGU(S)	0.2
AUC(I)	0.79	ACC(T)	1.45	AAC(N)	1.42	AGC(S)	1.07
AUA(M)	1.14	ACA(T)	1.5	AAA(K)	2	AGA(*)	0
AUG(M)	0.86	ACG(T)	0.13	AAG(K)	0	AGG(*)	0
GUU(V)	1.24	GCU(A)	0.95	GAU(D)	0.54	GGU(G)	0.56
GUC(V)	1.33	GCC(A)	1.67	GAC(D)	1.46	GGC(G)	1.43
GUA(V)	1.17	GCA(A)	1.27	GAA(E)	1.69	GGA(G)	1.38
GUG(V)	0.26	GCG(A)	0.12	GAG(E)	0.31	GGG(G)	0.64
C.ignobilis							
Codon	RCSU	Codon	RCSU	Codon	RCSU	Codon	RCSU
UUU(F)	0.71	UCU(S)	0.77	UAU(Y)	0.76	UGU(C)	0.34
UUC(F)	1.29	UCC(S)	1.88	UAC(Y)	1.24	UGC(C)	1.66
UUA(L)	0.7	UCA(S)	1.76	UAA(*)	1.8	UGA(W)	1.81
UUG(L)	0.06	UCG(S)	0.15	UAG(*)	0.2	UGG(W)	0.19
CUU(L)	1.49	CCU(P)	0.91	CAU(H)	0.61	CGU(R)	0.43
CUC(L)	1.57	CCC(P)	1.67	CAC(H)	1.39	CGC(R)	1.16
CUA(L)	1.93	CCA(P)	1.24	CAA(Q)	1.82	CGA(R)	2.12
CUG(L)	0.26	CCG(P)	0.17	CAG(Q)	0.18	CGG(R)	0.29
AUU(I)	1	ACU(T)	0.72	AAU(N)	0.65	AGU(S)	0.42
AUC(I)	1	ACC(T)	1.54	AAC(N)	1.35	AGC(S)	1.02

AUA(M)	1.29	ACA(T)	1.52	AAA(K)	1.77	AGA(*)	1.4
AUG(M)	0.71	ACG(T)	0.22	AAG(K)	0.23	AGG(*)	0.6
GUU(V)	0.99	GCU(A)	0.75	GAU(D)	0.72	GGU(G)	0.5
GUC(V)	1.26	GCC(A)	1.71	GAC(D)	1.28	GGC(G)	1.42
GUA(V)	1.55	GCA(A)	1.35	GAA(E)	1.77	GGA(G)	1.61
GUG(V)	0.21	GCG(A)	0.2	GAG(E)	0.23	GGG(G)	0.47
C.melampygus							
Codon	RCSU	Codon	RCSU	Codon	RCSU	Codon	RCSU
UUU(F)	0.75	UCU(S)	0.84	UAU(Y)	0.77	UGU(C)	0.41
UUC(F)	1.25	UCC(S)	1.81	UAC(Y)	1.23	UGC(C)	1.59
UUA(L)	0.75	UCA(S)	1.79	UAA(*)	1.52	UGA(W)	1.86
UUG(L)	0.08	UCG(S)	0.12	UAG(*)	0.57	UGG(W)	0.14
CUU(L)	1.51	CCU(P)	0.91	CAU(H)	0.56	CGU(R)	0.4
CUC(L)	1.51	CCC(P)	1.58	CAC(H)	1.44	CGC(R)	1.23
CUA(L)	1.92	CCA(P)	1.34	CAA(Q)	1.89	CGA(R)	2.32
CUG(L)	0.24	CCG(P)	0.17	CAG(Q)	0.11	CGG(R)	0.05
AUU(I)	1.2	ACU(T)	0.67	AAU(N)	0.6	AGU(S)	0.2
AUC(I)	0.8	ACC(T)	1.58	AAC(N)	1.4	AGC(S)	1.24
AUA(M)	1.28	ACA(T)	1.5	AAA(K)	1.84	AGA(*)	1.33
AUG(M)	0.72	ACG(T)	0.25	AAG(K)	0.16	AGG(*)	0.57
GUU(V)	1.13	GCU(A)	0.73	GAU(D)	0.56	GGU(G)	0.58
GUC(V)	1.25	GCC(A)	1.76	GAC(D)	1.44	GGC(G)	1.37
GUA(V)	1.45	GCA(A)	1.45	GAA(E)	1.77	GGA(G)	1.68
GUG(V)	0.18	GCG(A)	0.06	GAG(E)	0.23	GGG(G)	0.38
C.tille							
Codon	RCSU	Codon	RCSU	Codon	RCSU	Codon	RCSU
UUU(F)	0.74	UCU(S)	0.84	UAU(Y)	0.77	UGU(C)	0.41
UUC(F)	1.26	UCC(S)	1.81	UAC(Y)	1.23	UGC(C)	1.59
UUA(L)	0.75	UCA(S)	1.79	UAA(*)	1.52	UGA(W)	1.85
UUG(L)	0.08	UCG(S)	0.12	UAG(*)	0.57	UGG(W)	0.15
CUU(L)	1.52	CCU(P)	0.89	CAU(H)	0.55	CGU(R)	0.45
CUC(L)	1.5	CCC(P)	1.6	CAC(H)	1.45	CGC(R)	1.15
CUA(L)	1.9	CCA(P)	1.32	CAA(Q)	1.87	CGA(R)	2.35
CUG(L)	0.26	CCG(P)	0.19	CAG(Q)	0.13	CGG(R)	0.05
AUU(I)	1.19	ACU(T)	0.66	AAU(N)	0.59	AGU(S)	0.2
AUC(I)	0.81	ACC(T)	1.58	AAC(N)	1.41	AGC(S)	1.24
AUA(M)	1.29	ACA(T)	1.51	AAA(K)	1.84	AGA(*)	1.33
AUG(M)	0.71	ACG(T)	0.25	AAG(K)	0.16	AGG(*)	0.57
GUU(V)	1.14	GCU(A)	0.73	GAU(D)	0.58	GGU(G)	0.58
GUC(V)	1.24	GCC(A)	1.75	GAC(D)	1.42	GGC(G)	1.37
GUA(V)	1.44	GCA(A)	1.46	GAA(E)	1.79	GGA(G)	1.73
GUG(V)	0.18	GCG(A)	0.06	GAG(E)	0.21	GGG(G)	0.32
D.macarellus							
Codon	RCSU	Codon	RCSU	Codon	RCSU	Codon	RCSU
UUU(F)	0.75	UCU(S)	0.82	UAU(Y)	0.58	UGU(C)	0.32
UUC(F)	1.25	UCC(S)	2.2	UAC(Y)	1.42	UGC(C)	1.68
UUA(L)	0.57	UCA(S)	1.32	UAA(*)	1.33	UGA(W)	1.68
UUG(L)	0.19	UCG(S)	0.52	UAG(*)	1.44	UGG(W)	0.32
CUU(L)	1.52	CCU(P)	0.69	CAU(H)	0.52	CGU(R)	0.54
CUC(L)	1.56	CCC(P)	1.76	CAC(H)	1.48	CGC(R)	1.14
CUA(L)	1.55	CCA(P)	1.09	CAA(Q)	1.56	CGA(R)	1.68
CUG(L)	0.6	CCG(P)	0.46	CAG(Q)	0.44	CGG(R)	0.64
AUU(I)	1.01	ACU(T)	0.57	AAU(N)	0.57	AGU(S)	0.08

AUC(I)	0.99	ACC(T)	1.66	AAC(N)	1.43	AGC(S)	1.06
AUA(M)	1.01	ACA(T)	1.36	AAA(K)	1.69	AGA(*)	0.78
AUG(M)	0.99	ACG(T)	0.41	AAG(K)	0.31	AGG(*)	0.44
GUU(V)	1.15	GCU(A)	0.65	GAU(D)	0.4	GGU(G)	0.43
GUC(V)	1.51	GCC(A)	2.03	GAC(D)	1.6	GGC(G)	1.65
GUA(V)	1.25	GCA(A)	1.13	GAA(E)	1.42	GGA(G)	1.34
GUG(V)	0.09	GCG(A)	0.19	GAG(E)	0.58	GGG(G)	0.58
D.macrosoma							
Codon	RCSU	Codon	RCSU	Codon	RCSU	Codon	RCSU
UUU(F)	0.63	UCU(S)	0.7	UAU(Y)	0.53	UGU(C)	0.15
UUC(F)	1.37	UCC(S)	2.13	UAC(Y)	1.47	UGC(C)	1.85
UUA(L)	0.53	UCA(S)	1.38	UAA(*)	1.28	UGA(W)	1.76
UUG(L)	0.06	UCG(S)	0.38	UAG(*)	0.96	UGG(W)	0.24
CUU(L)	1.56	CCU(P)	0.76	CAU(H)	0.52	CGU(R)	0.4
CUC(L)	1.81	CCC(P)	1.93	CAC(H)	1.48	CGC(R)	1.19
CUA(L)	1.43	CCA(P)	1.05	CAA(Q)	1.43	CGA(R)	2.07
CUG(L)	0.62	CCG(P)	0.27	CAG(Q)	0.57	CGG(R)	0.35
AUU(I)	0.87	ACU(T)	0.7	AAU(N)	0.49	AGU(S)	0.2
AUC(I)	1.13	ACC(T)	1.62	AAC(N)	1.51	AGC(S)	1.21
AUA(M)	0.87	ACA(T)	1.35	AAA(K)	1.69	AGA(*)	0.96
AUG(M)	1.13	ACG(T)	0.33	AAG(K)	0.31	AGG(*)	0.8
GUU(V)	1.09	GCU(A)	0.69	GAU(D)	0.26	GGU(G)	0.46
GUC(V)	1.58	GCC(A)	1.96	GAC(D)	1.74	GGC(G)	1.7
GUA(V)	1.15	GCA(A)	1.14	GAA(E)	1.66	GGA(G)	1.32
GUG(V)	0.17	GCG(A)	0.2	GAG(E)	0.34	GGG(G)	0.53
D.maruadsi							
Codon	RCSU	Codon	RCSU	Codon	RCSU	Codon	RCSU
UUU(F)	0.69	UCU(S)	0.71	UAU(Y)	0.53	UGU(C)	0.38
UUC(F)	1.31	UCC(S)	2.09	UAC(Y)	1.47	UGC(C)	1.62
UUA(L)	0.47	UCA(S)	1.49	UAA(*)	1.8	UGA(W)	1.81
UUG(L)	0.05	UCG(S)	0.33	UAG(*)	0.2	UGG(W)	0.19
CUU(L)	1.5	CCU(P)	0.97	CAU(H)	0.48	CGU(R)	0.6
CUC(L)	1.76	CCC(P)	1.81	CAC(H)	1.52	CGC(R)	1.21
CUA(L)	1.82	CCA(P)	0.92	CAA(Q)	1.5	CGA(R)	1.77
CUG(L)	0.39	CCG(P)	0.3	CAG(Q)	0.5	CGG(R)	0.42
AUU(I)	0.96	ACU(T)	0.56	AAU(N)	0.45	AGU(S)	0.1
AUC(I)	1.04	ACC(T)	1.83	AAC(N)	1.55	AGC(S)	1.29
AUA(M)	0.93	ACA(T)	1.37	AAA(K)	1.82	AGA(*)	1.2
AUG(M)	1.07	ACG(T)	0.24	AAG(K)	0.18	AGG(*)	0.8
GUU(V)	1.15	GCU(A)	0.74	GAU(D)	0.39	GGU(G)	0.51
GUC(V)	1.44	GCC(A)	1.87	GAC(D)	1.61	GGC(G)	1.59
GUA(V)	1.29	GCA(A)	1.25	GAA(E)	1.67	GGA(G)	1.42
GUG(V)	0.12	GCG(A)	0.15	GAG(E)	0.33	GGG(G)	0.48
D.tabl							
Codon	RSCU	Codon	RSCU	Codon	RSCU	Codon	RSCU
UUU(F)	0.64	UCU(S)	1.07	UAU(Y)	0.58	UGU(C)	0.4
UUC(F)	1.36	UCC(S)	2.01	UAC(Y)	1.42	UGC(C)	1.6
UUA(L)	0.47	UCA(S)	1.41	UAA(*)	0	UGA(W)	1.78
UUG(L)	0.14	UCG(S)	0.26	UAG(*)	0	UGG(W)	0.22
CUU(L)	1.35	CCU(P)	1.04	CAU(H)	0.43	CGU(R)	0.46
CUC(L)	1.78	CCC(P)	1.7	CAC(H)	1.57	CGC(R)	0.97
CUA(L)	1.63	CCA(P)	1.16	CAA(Q)	1.57	CGA(R)	1.9
CUG(L)	0.64	CCG(P)	0.11	CAG(Q)	0.43	CGG(R)	0.67

AUU(I)	0.97	ACU(T)	0.52	AAU(N)	0.39	AGU(S)	0.1
AUC(I)	1.03	ACC(T)	1.88	AAC(N)	1.61	AGC(S)	1.15
AUA(M)	0.72	ACA(T)	1.37	AAA(K)	1.73	AGA(*)	0
AUG(M)	1.28	ACG(T)	0.23	AAG(K)	0.27	AGG(*)	0
GUU(V)	1.11	GCU(A)	0.62	GAU(D)	0.31	GGU(G)	0.64
GUC(V)	1.4	GCC(A)	2.01	GAC(D)	1.69	GGC(G)	1.36
GUA(V)	1.26	GCA(A)	1.1	GAA(E)	1.55	GGA(G)	1.26
GUG(V)	0.23	GCG(A)	0.27	GAG(E)	0.45	GGG(G)	0.74
E.bipinnulata							
Codon	RSCU	Codon	RSCU	Codon	RSCU	Codon	RSCU
UUU(F)	0.66	UCU(S)	1.35	UAU(Y)	0.78	UGU(C)	0.17
UUC(F)	1.34	UCC(S)	1.71	UAC(Y)	1.22	UGC(C)	1.83
UUA(L)	0.75	UCA(S)	1.56	UAA(*)	0	UGA(W)	1.69
UUG(L)	0.13	UCG(S)	0.13	UAG(*)	0	UGG(W)	0.31
CUU(L)	1.35	CCU(P)	1.09	CAU(H)	0.65	CGU(R)	0.41
CUC(L)	1.53	CCC(P)	1.57	CAC(H)	1.35	CGC(R)	0.67
CUA(L)	1.65	CCA(P)	1.1	CAA(Q)	1.67	CGA(R)	2.51
CUG(L)	0.59	CCG(P)	0.24	CAG(Q)	0.33	CGG(R)	0.41
AUU(I)	0.89	ACU(T)	0.81	AAU(N)	0.59	AGU(S)	0.31
AUC(I)	1.11	ACC(T)	1.42	AAC(N)	1.41	AGC(S)	0.94
AUA(M)	1.04	ACA(T)	1.61	AAA(K)	1.76	AGA(*)	0
AUG(M)	0.96	ACG(T)	0.16	AAG(K)	0.24	AGG(*)	0
GUU(V)	1.18	GCU(A)	0.75	GAU(D)	0.68	GGU(G)	0.63
GUC(V)	1.34	GCC(A)	1.72	GAC(D)	1.32	GGC(G)	1.27
GUA(V)	1.09	GCA(A)	1.32	GAA(E)	1.52	GGA(G)	1.44
GUG(V)	0.39	GCG(A)	0.21	GAG(E)	0.48	GGG(G)	0.66
G.speciosus							
Codon	RSCU	Codon	RSCU	Codon	RSCU	Codon	RSCU
UUU(F)	0.8	UCU(S)	0.96	UAU(Y)	0.86	UGU(C)	0.56
UUC(F)	1.2	UCC(S)	1.72	UAC(Y)	1.14	UGC(C)	1.44
UUA(L)	0.96	UCA(S)	1.84	UAA(*)	0	UGA(W)	1.87
UUG(L)	0.17	UCG(S)	0.22	UAG(*)	0	UGG(W)	0.13
CUU(L)	1.37	CCU(P)	1.16	CAU(H)	0.64	CGU(R)	0.68
CUC(L)	1.35	CCC(P)	1.41	CAC(H)	1.36	CGC(R)	0.84
CUA(L)	1.98	CCA(P)	1.34	CAA(Q)	1.92	CGA(R)	2.32
CUG(L)	0.17	CCG(P)	0.09	CAG(Q)	0.08	CGG(R)	0.16
AUU(I)	1.11	ACU(T)	0.64	AAU(N)	0.69	AGU(S)	0.3
AUC(I)	0.89	ACC(T)	1.6	AAC(N)	1.31	AGC(S)	0.96
AUA(M)	1.37	ACA(T)	1.68	AAA(K)	2	AGA(*)	0
AUG(M)	0.63	ACG(T)	0.08	AAG(K)	0	AGG(*)	0
GUU(V)	1.23	GCU(A)	0.9	GAU(D)	0.53	GGU(G)	0.72
GUC(V)	1.1	GCC(A)	1.68	GAC(D)	1.47	GGC(G)	1.32
GUA(V)	1.39	GCA(A)	1.34	GAA(E)	1.76	GGA(G)	1.53
GUG(V)	0.28	GCG(A)	0.08	GAG(E)	0.24	GGG(G)	0.42
M. cordyla							
Codon	RCSU	Codon	RCSU	Codon	RCSU	Codon	RCSU
UUU(F)	0.74	UCU(S)	0.78	UAU(Y)	0.69	UGU(C)	0.48
UUC(F)	1.26	UCC(S)	1.68	UAC(Y)	1.31	UGC(C)	1.52
UUA(L)	0.89	UCA(S)	1.86	UAA(*)	1.9	UGA(W)	1.86
UUG(L)	0.04	UCG(S)	0.13	UAG(*)	0	UGG(W)	0.14
CUU(L)	1.34	CCU(P)	0.65	CAU(H)	0.59	CGU(R)	0.63
CUC(L)	1.75	CCC(P)	1.87	CAC(H)	1.41	CGC(R)	1.07
CUA(L)	1.7	CCA(P)	1.32	CAA(Q)	1.86	CGA(R)	2.2

CUG(L)	0.29	CCG(P)	0.17	CAG(Q)	0.14	CGG(R)	0.1
AUU(I)	1.01	ACU(T)	0.63	AAU(N)	0.62	AGU(S)	0.28
AUC(I)	0.99	ACC(T)	1.58	AAC(N)	1.38	AGC(S)	1.28
AUA(M)	1.38	ACA(T)	1.55	AAA(K)	1.78	AGA(*)	1.33
AUG(M)	0.62	ACG(T)	0.25	AAG(K)	0.22	AGG(*)	0.76
GUU(V)	0.95	GCU(A)	0.75	GAU(D)	0.6	GGU(G)	0.49
GUC(V)	1.2	GCC(A)	1.79	GAC(D)	1.4	GGC(G)	1.47
GUA(V)	1.58	GCA(A)	1.37	GAA(E)	1.74	GGA(G)	1.55
GUG(V)	0.27	GCG(A)	0.09	GAG(E)	0.26	GGG(G)	0.49
P. niger							
Codon	RSCU	Codon	RSCU	Codon	RSCU	Codon	RSCU
UUU(F)	0.77	UCU(S)	1.16	UAU(Y)	0.86	UGU(C)	0.48
UUC(F)	1.23	UCC(S)	1.66	UAC(Y)	1.14	UGC(C)	1.52
UUA(L)	0.7	UCA(S)	1.89	UAA(*)	0	UGA(W)	1.77
UUG(L)	0.17	UCG(S)	0.08	UAG(*)	0	UGG(W)	0.23
CUU(L)	1.51	CCU(P)	1.07	CAU(H)	0.61	CGU(R)	0.36
CUC(L)	1.67	CCC(P)	1.62	CAC(H)	1.39	CGC(R)	1.04
CUA(L)	1.62	CCA(P)	1.22	CAA(Q)	1.9	CGA(R)	2.39
CUG(L)	0.33	CCG(P)	0.09	CAG(Q)	0.1	CGG(R)	0.21
AUU(I)	1.17	ACU(T)	0.68	AAU(N)	0.48	AGU(S)	0.13
AUC(I)	0.83	ACC(T)	1.57	AAC(N)	1.52	AGC(S)	1.09
AUA(M)	1.15	ACA(T)	1.61	AAA(K)	1.86	AGA(*)	0
AUG(M)	0.85	ACG(T)	0.15	AAG(K)	0.14	AGG(*)	0
GUU(V)	1.12	GCU(A)	0.84	GAU(D)	0.72	GGU(G)	0.48
GUC(V)	1.33	GCC(A)	1.62	GAC(D)	1.28	GGC(G)	1.39
GUA(V)	1.15	GCA(A)	1.41	GAA(E)	1.53	GGA(G)	1.46
GUG(V)	0.41	GCG(A)	0.14	GAG(E)	0.47	GGG(G)	0.67
P. dentex							
Codon	RSCU	Codon	RSCU	Codon	RSCU	Codon	RSCU
UUU(F)	0.79	UCU(S)	1.02	UAU(Y)	0.68	UGU(C)	0.44
UUC(F)	1.21	UCC(S)	1.86	UAC(Y)	1.32	UGC(C)	1.56
UUA(L)	0.67	UCA(S)	1.48	UAA(*)	0	UGA(W)	1.62
UUG(L)	0.23	UCG(S)	0.31	UAG(*)	0	UGG(W)	0.38
CUU(L)	1.37	CCU(P)	0.78	CAU(H)	0.43	CGU(R)	0.51
CUC(L)	1.62	CCC(P)	2.02	CAC(H)	1.57	CGC(R)	1.01
CUA(L)	1.62	CCA(P)	0.9	CAA(Q)	1.51	CGA(R)	1.97
CUG(L)	0.5	CCG(P)	0.3	CAG(Q)	0.49	CGG(R)	0.51
AUU(I)	1.01	ACU(T)	0.49	AAU(N)	0.61	AGU(S)	0.2
AUC(I)	0.99	ACC(T)	1.92	AAC(N)	1.39	AGC(S)	1.12
AUA(M)	1.29	ACA(T)	1.3	AAA(K)	1.46	AGA(*)	0
AUG(M)	0.71	ACG(T)	0.29	AAG(K)	0.54	AGG(*)	0
GUU(V)	1.03	GCU(A)	0.66	GAU(D)	0.5	GGU(G)	0.47
GUC(V)	1.41	GCC(A)	1.93	GAC(D)	1.5	GGC(G)	1.52
GUA(V)	1.12	GCA(A)	1.12	GAA(E)	1.27	GGA(G)	1.34
GUG(V)	0.45	GCG(A)	0.29	GAG(E)	0.73	GGG(G)	0.67
S. crumenophthalmus							
Codon	RCSU	Codon	RCSU	Codon	RCSU	Codon	RCSU
UUU(F)	0.69	UCU(S)	0.95	UAU(Y)	0.77	UGU(C)	0.37
UUC(F)	1.31	UCC(S)	1.65	UAC(Y)	1.23	UGC(C)	1.63
UUA(L)	0.79	UCA(S)	1.85	UAA(*)	1.88	UGA(W)	1.77
UUG(L)	0.06	UCG(S)	0.18	UAG(*)	0.71	UGG(W)	0.23
CUU(L)	1.54	CCU(P)	1	CAU(H)	0.46	CGU(R)	0.51
CUC(L)	1.83	CCC(P)	1.6	CAC(H)	1.54	CGC(R)	1.4

CUA(L)	1.37	CCA(P)	1.2	CAA(Q)	1.69	CGA(R)	1.63
CUG(L)	0.41	CCG(P)	0.2	CAG(Q)	0.31	CGG(R)	0.47
AUU(I)	1.19	ACU(T)	0.77	AAU(N)	0.69	AGU(S)	0.1
AUC(I)	0.81	ACC(T)	1.67	AAC(N)	1.31	AGC(S)	1.26
AUA(M)	1.06	ACA(T)	1.38	AAA(K)	1.85	AGA(*)	0.71
AUG(M)	0.94	ACG(T)	0.18	AAG(K)	0.15	AGG(*)	0.71
GUU(V)	1.51	GCU(A)	0.66	GAU(D)	0.57	GGU(G)	0.71
GUC(V)	1.21	GCC(A)	2.04	GAC(D)	1.43	GGC(G)	1.59
GUA(V)	1.15	GCA(A)	1.13	GAA(E)	1.56	GGA(G)	1.17
GUG(V)	0.13	GCG(A)	0.17	GAG(E)	0.44	GGG(G)	0.53
S. leptolepis							
Codon	RSCU	Codon	RSCU	Codon	RSCU	Codon	RSCU
UUU(F)	0.78	UCU(S)	1.23	UAU(Y)	0.74	UGU(C)	0.62
UUC(F)	1.22	UCC(S)	1.85	UAC(Y)	1.26	UGC(C)	1.38
UUA(L)	0.92	UCA(S)	1.44	UAA(*)	0	UGA(W)	1.75
UUG(L)	0.2	UCG(S)	0.23	UAG(*)	0	UGG(W)	0.25
CUU(L)	1.66	CCU(P)	1.21	CAU(H)	0.5	CGU(R)	0.82
CUC(L)	1.15	CCC(P)	1.52	CAC(H)	1.5	CGC(R)	0.87
CUA(L)	1.71	CCA(P)	1.09	CAA(Q)	1.59	CGA(R)	1.79
CUG(L)	0.36	CCG(P)	0.18	CAG(Q)	0.41	CGG(R)	0.51
AUU(I)	1.17	ACU(T)	0.64	AAU(N)	0.6	AGU(S)	0.13
AUC(I)	0.83	ACC(T)	1.77	AAC(N)	1.4	AGC(S)	1.13
AUA(M)	0.91	ACA(T)	1.38	AAA(K)	1.78	AGA(*)	0
AUG(M)	1.09	ACG(T)	0.21	AAG(K)	0.22	AGG(*)	0
GUU(V)	1.25	GCU(A)	0.76	GAU(D)	0.49	GGU(G)	0.44
GUC(V)	1.05	GCC(A)	1.86	GAC(D)	1.51	GGC(G)	1.15
GUA(V)	1.38	GCA(A)	1.24	GAA(E)	1.6	GGA(G)	1.56
GUG(V)	0.32	GCG(A)	0.14	GAG(E)	0.4	GGG(G)	0.85
S. dumerili							
Codon	RCSU	Codon	RCSU	Codon	RCSU	Codon	RCSU
UUU(F)	0.76	UCU(S)	1.07	UAU(Y)	0.63	UGU(C)	0.68
UUC(F)	1.24	UCC(S)	2.32	UAC(Y)	1.37	UGC(C)	1.32
UUA(L)	1.27	UCA(S)	1.19	UAA(*)	1.95	UGA(W)	1.37
UUG(L)	0.57	UCG(S)	0.77	UAG(*)	1.56	UGG(W)	0.63
CUU(L)	1.19	CCU(P)	1.14	CAU(H)	0.72	CGU(R)	0.58
CUC(L)	1.17	CCC(P)	1.65	CAC(H)	1.28	CGC(R)	0.81
CUA(L)	1.22	CCA(P)	0.81	CAA(Q)	1.21	CGA(R)	1.97
CUG(L)	0.58	CCG(P)	0.4	CAG(Q)	0.79	CGG(R)	0.64
AUU(I)	1.04	ACU(T)	0.73	AAU(N)	0.57	AGU(S)	0.16
AUC(I)	0.96	ACC(T)	1.35	AAC(N)	1.43	AGC(S)	0.48
AUA(M)	1.1	ACA(T)	1.35	AAA(K)	1.42	AGA(*)	0.39
AUG(M)	0.9	ACG(T)	0.58	AAG(K)	0.58	AGG(*)	0.1
GUU(V)	1.15	GCU(A)	0.83	GAU(D)	0.65	GGU(G)	0.66
GUC(V)	1.51	GCC(A)	1.86	GAC(D)	1.35	GGC(G)	1.13
GUA(V)	0.97	GCA(A)	1.08	GAA(E)	1.28	GGA(G)	1.33
GUG(V)	0.36	GCG(A)	0.22	GAG(E)	0.72	GGG(G)	0.88
S. lalandi							
Codon	RCSU	Codon	RCSU	Codon	RCSU	Codon	RCSU
UUU(F)	0.72	UCU(S)	0.71	UAU(Y)	0.44	UGU(C)	0.62
UUC(F)	1.28	UCC(S)	1.97	UAC(Y)	1.56	UGC(C)	1.38
UUA(L)	0.67	UCA(S)	1.49	UAA(*)	2	UGA(W)	1.55
UUG(L)	0.08	UCG(S)	0.45	UAG(*)	0.8	UGG(W)	0.45
CUU(L)	1.31	CCU(P)	0.88	CAU(H)	0.6	CGU(R)	0.31

CUC(L)	1.72	CCC(P)	1.95	CAC(H)	1.4	CGC(R)	0.99
CUA(L)	1.56	CCA(P)	0.85	CAA(Q)	1.67	CGA(R)	2.11
CUG(L)	0.66	CCG(P)	0.32	CAG(Q)	0.33	CGG(R)	0.58
AUU(I)	0.9	ACU(T)	0.61	AAU(N)	0.43	AGU(S)	0.24
AUC(I)	1.1	ACC(T)	1.55	AAC(N)	1.57	AGC(S)	1.15
AUA(M)	1.12	ACA(T)	1.39	AAA(K)	1.49	AGA(*)	1
AUG(M)	0.88	ACG(T)	0.46	AAG(K)	0.51	AGG(*)	0.2
GUU(V)	1.17	GCU(A)	0.64	GAU(D)	0.49	GGU(G)	0.51
GUC(V)	1.55	GCC(A)	1.97	GAC(D)	1.51	GGC(G)	1.5
GUA(V)	0.94	GCA(A)	1.14	GAA(E)	1.47	GGA(G)	1.29
GUG(V)	0.34	GCG(A)	0.26	GAG(E)	0.53	GGG(G)	0.7
S quinqueradiata							
Codon	RCSU	Codon	RCSU	Codon	RCSU	Codon	RCSU
UUU(F)	0.72	UCU(S)	0.75	UAU(Y)	0.5	UGU(C)	0.31
UUC(F)	1.28	UCC(S)	1.91	UAC(Y)	1.5	UGC(C)	1.69
UUA(L)	0.59	UCA(S)	1.42	UAA(*)	1.52	UGA(W)	1.47
UUG(L)	0.11	UCG(S)	0.44	UAG(*)	0.76	UGG(W)	0.53
CUU(L)	1.34	CCU(P)	0.84	CAU(H)	0.52	CGU(R)	0.28
CUC(L)	1.57	CCC(P)	1.99	CAC(H)	1.48	CGC(R)	0.97
CUA(L)	1.62	CCA(P)	0.75	CAA(Q)	1.58	CGA(R)	2.07
CUG(L)	0.76	CCG(P)	0.42	CAG(Q)	0.42	CGG(R)	0.69
AUU(I)	1.01	ACU(T)	0.6	AAU(N)	0.36	AGU(S)	0.33
AUC(I)	0.99	ACC(T)	1.54	AAC(N)	1.64	AGC(S)	1.16
AUA(M)	1.11	ACA(T)	1.49	AAA(K)	1.39	AGA(*)	1.33
AUG(M)	0.89	ACG(T)	0.36	AAG(K)	0.61	AGG(*)	0.38
GUU(V)	1.23	GCU(A)	0.59	GAU(D)	0.39	GGU(G)	0.43
GUC(V)	1.36	GCC(A)	2	GAC(D)	1.61	GGC(G)	1.54
GUA(V)	1.14	GCA(A)	1.1	GAA(E)	1.51	GGA(G)	1.09
GUG(V)	0.26	GCG(A)	0.31	GAG(E)	0.49	GGG(G)	0.93
S. rivoliana							
Codon	RSCU	Codon	RSCU	Codon	RSCU	Codon	RSCU
UUU(F)	0.75	UCU(S)	0.97	UAU(Y)	0.53	UGU(C)	0.5
UUC(F)	1.25	UCC(S)	1.83	UAC(Y)	1.47	UGC(C)	1.5
UUA(L)	0.81	UCA(S)	1.54	UAA(*)	0	UGA(W)	1.65
UUG(L)	0.19	UCG(S)	0.37	UAG(*)	0	UGG(W)	0.35
CUU(L)	1.36	CCU(P)	1.12	CAU(H)	0.42	CGU(R)	0.3
CUC(L)	1.51	CCC(P)	1.74	CAC(H)	1.58	CGC(R)	0.7
CUA(L)	1.61	CCA(P)	0.96	CAA(Q)	1.7	CGA(R)	2.3
CUG(L)	0.53	CCG(P)	0.18	CAG(Q)	0.3	CGG(R)	0.7
AUU(I)	1.03	ACU(T)	0.66	AAU(N)	0.47	AGU(S)	0.26
AUC(I)	0.97	ACC(T)	1.54	AAC(N)	1.53	AGC(S)	1.04
AUA(M)	1.19	ACA(T)	1.62	AAA(K)	1.64	AGA(*)	0
AUG(M)	0.81	ACG(T)	0.18	AAG(K)	0.36	AGG(*)	0
GUU(V)	1.31	GCU(A)	0.66	GAU(D)	0.4	GGU(G)	0.67
GUC(V)	1.29	GCC(A)	1.84	GAC(D)	1.6	GGC(G)	1.26
GUA(V)	0.89	GCA(A)	1.31	GAA(E)	1.64	GGA(G)	1.32
GUG(V)	0.51	GCG(A)	0.19	GAG(E)	0.36	GGG(G)	0.75
S. nigrofasciata							
Codon	RSCU	Codon	RSCU	Codon	RSCU	Codon	RSCU
UUU(F)	0.82	UCU(S)	0.9	UAU(Y)	0.44	UGU(C)	0.42
UUC(F)	1.18	UCC(S)	2.04	UAC(Y)	1.56	UGC(C)	1.58
UUA(L)	0.73	UCA(S)	1.4	UAA(*)	0	UGA(W)	1.53
UUG(L)	0.22	UCG(S)	0.37	UAG(*)	0	UGG(W)	0.47

CUU(L)	1.43	CCU(P)	1.07	CAU(H)	0.5	CGU(R)	0.46
CUC(L)	1.62	CCC(P)	1.7	CAC(H)	1.5	CGC(R)	0.81
CUA(L)	1.42	CCA(P)	0.89	CAA(Q)	1.66	CGA(R)	2.08
CUG(L)	0.57	CCG(P)	0.33	CAG(Q)	0.34	CGG(R)	0.66
AUU(I)	1.04	ACU(T)	0.76	AAU(N)	0.42	AGU(S)	0.4
AUC(I)	0.96	ACC(T)	1.53	AAC(N)	1.58	AGC(S)	0.9
AUA(M)	1.13	ACA(T)	1.42	AAA(K)	1.61	AGA(*)	0
AUG(M)	0.87	ACG(T)	0.3	AAG(K)	0.39	AGG(*)	0
GUU(V)	1.11	GCU(A)	0.58	GAU(D)	0.43	GGU(G)	0.54
GUC(V)	1.38	GCC(A)	1.89	GAC(D)	1.57	GGC(G)	1.49
GUA(V)	1.07	GCA(A)	1.33	GAA(E)	1.44	GGA(G)	1.07
GUG(V)	0.44	GCG(A)	0.19	GAG(E)	0.56	GGG(G)	0.9
T. blochii							
Codon	RCSU	Codon	RCSU	Codon	RCSU	Codon	RCSU
UUU(F)	0.77	UCU(S)	1.17	UAU(Y)	0.8	UGU(C)	0.43
UUC(F)	1.23	UCC(S)	1.83	UAC(Y)	1.2	UGC(C)	1.57
UUA(L)	1.28	UCA(S)	1.77	UAA(*)	1.94	UGA(W)	1.68
UUG(L)	0.51	UCG(S)	0.63	UAG(*)	1.71	UGG(W)	0.32
CUU(L)	1.05	CCU(P)	1.06	CAU(H)	0.94	CGU(R)	0.33
CUC(L)	1.23	CCC(P)	1.5	CAC(H)	1.06	CGC(R)	0.79
CUA(L)	1.56	CCA(P)	1.09	CAA(Q)	1.28	CGA(R)	2.23
CUG(L)	0.37	CCG(P)	0.36	CAG(Q)	0.72	CGG(R)	0.66
AUU(I)	1.15	ACU(T)	0.74	AAU(N)	0.69	AGU(S)	0.18
AUC(I)	0.85	ACC(T)	1.46	AAC(N)	1.31	AGC(S)	0.41
AUA(M)	1.19	ACA(T)	1.46	AAA(K)	1.47	AGA(*)	0.26
AUG(M)	0.81	ACG(T)	0.34	AAG(K)	0.53	AGG(*)	0.1
GUU(V)	1.28	GCU(A)	1.09	GAU(D)	0.76	GGU(G)	0.9
GUC(V)	1.06	GCC(A)	1.76	GAC(D)	1.24	GGC(G)	1.14
GUA(V)	1.23	GCA(A)	1.09	GAA(E)	1.44	GGA(G)	1.65
GUG(V)	0.42	GCG(A)	0.06	GAG(E)	0.56	GGG(G)	0.31
T. carolinus							
Codon	RCSU	Codon	RCSU	Codon	RCSU	Codon	RCSU
UUU(F)	0.8	UCU(S)	1.13	UAU(Y)	0.74	UGU(C)	0.53
UUC(F)	1.2	UCC(S)	1.4	UAC(Y)	1.26	UGC(C)	1.47
UUA(L)	0.69	UCA(S)	1.64	UAA(*)	0.89	UGA(W)	1.81
UUG(L)	0.16	UCG(S)	0.2	UAG(*)	0	UGG(W)	0.19
CUU(L)	1.33	CCU(P)	1.09	CAU(H)	0.63	CGU(R)	0.73
CUC(L)	1.47	CCC(P)	1.35	CAC(H)	1.37	CGC(R)	1.08
CUA(L)	1.89	CCA(P)	1.22	CAA(Q)	1.61	CGA(R)	1.51
CUG(L)	0.46	CCG(P)	0.35	CAG(Q)	0.39	CGG(R)	0.69
AUU(I)	0.98	ACU(T)	0.81	AAU(N)	0.78	AGU(S)	0.32
AUC(I)	1.02	ACC(T)	1.48	AAC(N)	1.22	AGC(S)	1.32
AUA(M)	1.22	ACA(T)	1.5	AAA(K)	1.68	AGA(*)	1.33
AUG(M)	0.78	ACG(T)	0.21	AAG(K)	0.32	AGG(*)	1.78
GUU(V)	1.13	GCU(A)	0.95	GAU(D)	0.59	GGU(G)	0.82
GUC(V)	1.31	GCC(A)	1.77	GAC(D)	1.41	GGC(G)	1.09
GUA(V)	1.33	GCA(A)	1.12	GAA(E)	1.68	GGA(G)	1.45
GUG(V)	0.23	GCG(A)	0.16	GAG(E)	0.32	GGG(G)	0.64
T. ovatus							
Codon	RCSU	Codon	RCSU	Codon	RCSU	Codon	RCSU
UUU(F)	0.8	UCU(S)	0.78	UAU(Y)	0.7	UGU(C)	0.64
UUC(F)	1.2	UCC(S)	1.59	UAC(Y)	1.3	UGC(C)	1.36
UUA(L)	0.71	UCA(S)	2.07	UAA(*)	1.91	UGA(W)	1.88

UUG(L)	0.06	UCG(S)	0.1	UAG(*)	0.52	UGG(W)	0.12
CUU(L)	1.3	CCU(P)	0.91	CAU(H)	0.64	CGU(R)	0.62
CUC(L)	1.67	CCC(P)	1.61	CAC(H)	1.36	CGC(R)	0.77
CUA(L)	1.88	CCA(P)	1.26	CAA(Q)	1.62	CGA(R)	2.36
CUG(L)	0.38	CCG(P)	0.23	CAG(Q)	0.38	CGG(R)	0.26
AUU(I)	1.02	ACU(T)	0.66	AAU(N)	0.64	AGU(S)	0.37
AUC(I)	0.98	ACC(T)	1.72	AAC(N)	1.36	AGC(S)	1.1
AUA(M)	1.32	ACA(T)	1.5	AAA(K)	1.82	AGA(*)	1.22
AUG(M)	0.68	ACG(T)	0.12	AAG(K)	0.18	AGG(*)	0.35
GUU(V)	1.21	GCU(A)	0.86	GAU(D)	0.53	GGU(G)	0.62
GUC(V)	1.11	GCC(A)	1.77	GAC(D)	1.47	GGC(G)	1.25
GUA(V)	1.51	GCA(A)	1.28	GAA(E)	1.68	GGA(G)	1.59
GUG(V)	0.17	GCG(A)	0.09	GAG(E)	0.32	GGG(G)	0.54
T. japonicus							
Codon	RCSU	Codon	RCSU	Codon	RCSU	Codon	RCSU
UUU(F)	0.73	UCU(S)	1.07	UAU(Y)	0.39	UGU(C)	0.28
UUC(F)	1.27	UCC(S)	1.71	UAC(Y)	1.61	UGC(C)	1.72
UUA(L)	0.58	UCA(S)	1.54	UAA(*)	1.5	UGA(W)	1.84
UUG(L)	0.08	UCG(S)	0.27	UAG(*)	0.5	UGG(W)	0.16
CUU(L)	1.58	CCU(P)	0.85	CAU(H)	0.57	CGU(R)	0.48
CUC(L)	1.67	CCC(P)	1.98	CAC(H)	1.43	CGC(R)	1.11
CUA(L)	1.7	CCA(P)	1	CAA(Q)	1.72	CGA(R)	1.98
CUG(L)	0.39	CCG(P)	0.17	CAG(Q)	0.28	CGG(R)	0.43
AUU(I)	1.08	ACU(T)	0.63	AAU(N)	0.49	AGU(S)	0.22
AUC(I)	0.92	ACC(T)	1.77	AAC(N)	1.51	AGC(S)	1.19
AUA(M)	1.07	ACA(T)	1.45	AAA(K)	1.71	AGA(*)	1.33
AUG(M)	0.93	ACG(T)	0.15	AAG(K)	0.29	AGG(*)	0.67
GUU(V)	1.09	GCU(A)	0.71	GAU(D)	0.46	GGU(G)	0.39
GUC(V)	1.55	GCC(A)	1.8	GAC(D)	1.54	GGC(G)	1.61
GUA(V)	1.28	GCA(A)	1.38	GAA(E)	1.72	GGA(G)	1.54
GUG(V)	0.08	GCG(A)	0.11	GAG(E)	0.28	GGG(G)	0.45
T. trachurus							
Codon	RCSU	Codon	RCSU	Codon	RCSU	Codon	RCSU
UUU(F)	0.68	UCU(S)	1.04	UAU(Y)	0.44	UGU(C)	0.3
UUC(F)	1.32	UCC(S)	1.78	UAC(Y)	1.56	UGC(C)	1.7
UUA(L)	0.52	UCA(S)	1.46	UAA(*)	1.6	UGA(W)	1.88
UUG(L)	0.06	UCG(S)	0.35	UAG(*)	0.48	UGG(W)	0.12
CUU(L)	1.68	CCU(P)	0.89	CAU(H)	0.55	CGU(R)	0.38
CUC(L)	1.59	CCC(P)	1.92	CAC(H)	1.45	CGC(R)	1.24
CUA(L)	1.7	CCA(P)	1	CAA(Q)	1.6	CGA(R)	2.05
CUG(L)	0.45	CCG(P)	0.19	CAG(Q)	0.4	CGG(R)	0.33
AUU(I)	1.02	ACU(T)	0.71	AAU(N)	0.57	AGU(S)	0.15
AUC(I)	0.98	ACC(T)	1.64	AAC(N)	1.43	AGC(S)	1.23
AUA(M)	1.03	ACA(T)	1.52	AAA(K)	1.75	AGA(*)	1.28
AUG(M)	0.97	ACG(T)	0.12	AAG(K)	0.25	AGG(*)	0.64
GUU(V)	1.04	GCU(A)	0.67	GAU(D)	0.43	GGU(G)	0.46
GUC(V)	1.51	GCC(A)	1.83	GAC(D)	1.57	GGC(G)	1.64
GUA(V)	1.35	GCA(A)	1.36	GAA(E)	1.71	GGA(G)	1.49
GUG(V)	0.1	GCG(A)	0.15	GAG(E)	0.29	GGG(G)	0.41
U. helvola							
Codon	RSCU	Codon	RSCU	Codon	RSCU	Codon	RSCU
UUU(F)	0.72	UCU(S)	1.14	UAU(Y)	0.72	UGU(C)	0.32
UUC(F)	1.28	UCC(S)	1.71	UAC(Y)	1.28	UGC(C)	1.68

UUA(L)	0.69	UCA(S)	1.77	UAA(*)	0	UGA(W)	1.75
UUG(L)	0.11	UCG(S)	0.18	UAG(*)	0	UGG(W)	0.25
CUU(L)	1.55	CCU(P)	1.07	CAU(H)	0.59	CGU(R)	0.47
CUC(L)	1.61	CCC(P)	1.72	CAC(H)	1.41	CGC(R)	0.88
CUA(L)	1.65	CCA(P)	1.03	CAA(Q)	1.71	CGA(R)	2.13
CUG(L)	0.38	CCG(P)	0.18	CAG(Q)	0.29	CGG(R)	0.52
AUU(I)	1.14	ACU(T)	0.63	AAU(N)	0.6	AGU(S)	0.18
AUC(I)	0.86	ACC(T)	1.7	AAC(N)	1.4	AGC(S)	1.01
AUA(M)	1.2	ACA(T)	1.55	AAA(K)	1.78	AGA(*)	0
AUG(M)	0.8	ACG(T)	0.12	AAG(K)	0.22	AGG(*)	0
GUU(V)	1.28	GCU(A)	0.85	GAU(D)	0.63	GGU(G)	0.6
GUC(V)	1.24	GCC(A)	1.73	GAC(D)	1.38	GGC(G)	1.39
GUA(V)	1.16	GCA(A)	1.27	GAA(E)	1.65	GGA(G)	1.27
GUG(V)	0.32	GCG(A)	0.15	GAG(E)	0.35	GGG(G)	0.74
U. secunda							
Codon	RSCU	Codon	RSCU	Codon	RSCU	Codon	RSCU
UUU(F)	0.72	UCU(S)	1.14	UAU(Y)	0.72	UGU(C)	0.32
UUC(F)	1.28	UCC(S)	1.71	UAC(Y)	1.28	UGC(C)	1.68
UUA(L)	0.7	UCA(S)	1.77	UAA(*)	0	UGA(W)	1.75
UUG(L)	0.11	UCG(S)	0.18	UAG(*)	0	UGG(W)	0.25
CUU(L)	1.53	CCU(P)	1.12	CAU(H)	0.61	CGU(R)	0.47
CUC(L)	1.63	CCC(P)	1.68	CAC(H)	1.39	CGC(R)	0.88
CUA(L)	1.64	CCA(P)	1.03	CAA(Q)	1.67	CGA(R)	2.13
CUG(L)	0.39	CCG(P)	0.18	CAG(Q)	0.33	CGG(R)	0.52
AUU(I)	1.14	ACU(T)	0.64	AAU(N)	0.59	AGU(S)	0.18
AUC(I)	0.86	ACC(T)	1.69	AAC(N)	1.41	AGC(S)	1.01
AUA(M)	1.21	ACA(T)	1.55	AAA(K)	1.78	AGA(*)	0
AUG(M)	0.79	ACG(T)	0.12	AAG(K)	0.22	AGG(*)	0
GUU(V)	1.26	GCU(A)	0.85	GAU(D)	0.64	GGU(G)	0.6
GUC(V)	1.24	GCC(A)	1.74	GAC(D)	1.36	GGC(G)	1.39
GUA(V)	1.18	GCA(A)	1.27	GAA(E)	1.67	GGA(G)	1.27
GUG(V)	0.32	GCG(A)	0.14	GAG(E)	0.33	GGG(G)	0.74

Table S7. Comparison of anticodons of tRNAs within 37 Carangidae species. *Dr=D. russelli, Ac= A. ciliaris, Ai= A. indica, Ad= A. djedaba, Ak= A. kleinii, Am= A. mate, Ca= C. armatus, Cb=C. bajad, Ce= C. equula, Cm=c. malabaricus, Cp= C. plagiotaenia, Ci= C. ignobilis, Cmel=C. melampygus, Ct= C. tille, Dmac = D. macerellus, Dm= D. macrosoma, Dmar= D. maruadsi,, Dt= D. tabl, Eb= E. bipinnulata, Gs= G. speciosus, Mc= M. cordyla, Pn= P. niger, Pd=P. dentex, Sc=S. crumenophthalmus, Sle= S. leptolepis, Sd= S. dumerili, Sl=S. lalandi, Sq=S. quinqueradiata, Sr= S. rivoliana, Sn= S. nigrofasciata, Tb=T. blochii, Tc=T. carolinus, To= T. ovatus, Tj= T. japonicas, Tt= T. trachurus, Uh= U. helvola, Us= U. secunda.*

Locus	tRNA F	tRNA V	tRNA L	tRNA I	tRNA Q	tRNA M	tRNA W	tRNA A	tRNA N	tRNA C	tRNA Y	tRNA S	tRNA D	tRNA K	tRNA G	tRNA R	tRNA H	tRNA S	tRNA L	tRNA E	tRNA T	tRNA P
Dr	GAA	TAC	TAA	GAT	TTG	CAT	TCA	TGC	GTT	GCA	GTA	TGA	GTC	TTT	TCC	TCG	GTG	GCT	TAG	TCC	TGT	TGG
Ac	GAA	TAC	TAA	GAT	TTG	CAT	TCA	TGC	GTT	GCA	GTA	TGA	GTC	TTT	TCC	TCG	GTG	GCT	TAG	TCC	TGT	TGG
Ai	GAA	TAC	TAA	GAT	TTG	CAT	TCA	TGC	GTT	GCA	GTA	TGA	GTC	TTT	TCC	TCG	GTG	GCT	TAG	TCC	TGT	TGG
Ad	GAA	TAC	TAA	GAT	TTG	CAT	TCA	TGC	GTT	GCA	GTA	TGA	GTC	TTT	TCC	TCG	GTG	GCT	TAG	TCC	TGT	TGG
Ak	GAA	TAC	TAA	GAT	TTG	CAT	TCA	TGC	GTT	GCA	GTA	TGA	GTC	TTT	TCC	TCG	GTG	GCT	TAG	TCC	TGT	TGG
Am	GAA	TAC	TAA	GAT	TTG	CAT	TCA	TGC	GTT	GCA	GTA	TGA	GTC	TTT	TCC	TCG	GTG	GCT	TAG	TCC	TGT	TGG
Ca	GAA	TAC	TAA	GAT	TTG	CAT	TCA	TGC	GTT	GCA	GTA	TGA	GTC	TTT	TCC	TCG	GTG	GCT	TAG	TCC	TGT	TGG
Cb	GAA	TAC	TAA	GAT	TTG	CAT	TCA	TGC	GTT	GCA	GTA	TGA	GTC	TTT	TCC	TCG	GTG	GCT	TAG	TCC	TGT	TGG
Ce	GAA	TAC	TAA	GAT	TTG	CAT	TCA	TGC	GTT	GCA	GTA	TGA	GTC	TTT	TCC	TCG	GTG	GCT	TAG	TCC	TGT	TGG
Cm	GAA	TAC	TAA	GAT	TTG	CAT	TCA	TGC	GTT	GCA	GTA	TGA	GTC	TTT	TCC	TCG	GTG	GCT	TAG	TCC	TGT	TGG
Cp	GAA	TAC	TAA	GAT	TTG	CAT	TCA	TGC	GTT	GCA	GTA	TGA	GTC	TTT	TCC	TCG	GTG	GCT	TAG	TCC	TGT	TGG
Ci	GAA	TAC	TAA	GAT	TTG	CAT	TCA	TGC	GTT	GCA	GTA	TGA	GTC	TTT	TCC	TCG	GTG	GCT	TAG	TCC	TGT	TGG
Cme	GAA	TAC	TAA	GAT	TTG	CAT	TCA	TGC	GTT	GCA	GTA	TGA	GTC	TTT	TCC	TCG	GTG	GCT	TAG	TCC	TGT	TGG
Ct	GAA	TAC	TAA	GAT	TTG	CAT	TCA	TGC	GTT	GCA	GTA	TGA	GTC	TTT	TCC	TCG	GTG	GCT	TAG	TCC	TGT	TGG
Dmac	GAA	TAC	TAA	GAT	TTG	CAT	TCA	TGC	GTT	GCA	GTA	TGA	GTC	TTT	TCC	TCG	GTG	GCT	TAG	TCC	TGT	TGG
Dm	GAA	TAC	TAA	GAT	TTG	CAT	TCA	TGC	GTT	GCA	GTA	TGA	GTC	TTT	TCC	TCG	GTG	GCT	TAG	TCC	TGT	TGG

Dmar	GAA	TAC	TAA	GAT	TTG	CAT	TCA	TGC	GTT	GCA	GTA	TGA	GTC	TTT	TCC	TCG	GTG	GCT	TAG	TCC	TGT	TGG
Dt	GAA	TAC	TAA	GAT	TTG	CAT	TCA	TGC	GTT	GCA	GTA	TGA	GTC	TTT	TCC	TCG	GTG	GCT	TAG	TCC	TGT	TGG
Eb	GAA	TAC	TAA	GAT	TTG	CAT	TCA	TGC	GTT	GCA	GTA	TGA	GTC	TTT	TCC	TCG	GTG	GCT	TAG	TCC	TGT	TGG
Gs	GAA	TAC	TAA	GAT	TTG	CAT	TCA	TGC	GTT	GCA	GTA	TGA	GTC	TTT	TCC	TCG	GTG	GCT	TAG	TCC	TGT	TGG
Mc	GAA	TAC	TAA	GAT	TTG	CAT	TCA	TGC	GTT	GCA	GTA	TGA	GTC	TTT	TCC	TCG	GTG	GCT	TAG	TCC	TGT	TGG
Pn	GAA	TAC	TAA	GAT	TTG	CAT	TCA	TGC	GTT	GCA	GTA	TGA	GTC	TTT	TCC	TCG	GTG	GCT	TAG	TCC	TGT	TGG
Pd	GAA	TAC	TAA	GAT	TTG	CAT	TCA	TGC	GTT	GCA	GTA	TGA	GTC	TTT	TCC	TCG	GTG	GCT	TAG	TCC	TGT	TGG
Sc	GAA	TAC	TAA	GAT	TTG	CAT	TCA	TGC	GTT	GCA	GTA	TGA	GTC	TTT	TCC	TCG	GTG	GCT	TAG	TCC	TGT	TGG
Sle	GAA	TAC	TAA	GAT	TTG	CAT	TCA	TGC	GTT	GCA	GTA	TGA	GTC	TTT	TCC	TCG	GTG	GCT	TAG	TCC	TGT	TGG
Sd	GAA	TAC	TAA	GAT	TTG	CAT	TCA	TGC	GTT	GCA	GTA	TGA	GTC	TTT	TCC	TCG	GTG	GCT	TAG	TCC	TGT	TGG
Sl	GAA	TAC	TAA	GAT	TTG	CAT	TCA	TGC	GTT	GCA	GTA	TGA	GTC	TTT	TCC	TCG	GTG	GCT	TAG	TCC	TGT	TGG
Sq	GAA	TAC	TAA	GAT	TTG	CAT	TCA	TGC	GTT	GCA	GTA	TGA	GTC	TTT	TCC	TCG	GTG	GCT	TAG	TCC	TGT	TGG
Sr	GAA	TAC	TAA	GAT	TTG	CAT	TCA	TGC	GTT	GCA	GTA	TGA	GTC	TTT	TCC	TCG	GTG	GCT	TAG	TCC	TGT	TGG
Sn	GAA	TAC	TAA	GAT	TTG	CAT	TCA	TGC	GTT	GCA	GTA	TGA	GTC	TTT	TCC	TCG	GTG	GCT	TAG	TCC	TGT	TGG
Tb	GAA	TAC	TAA	GAT	TTG	CAT	TCA	TGC	GTT	GCA	GTA	TGA	GTC	TTT	TCC	TCG	GTG	GCT	TAG	TCC	TGT	TGG
Tc	GAA	TAC	TAA	GAT	TTG	CAT	TCA	TGC	GTT	GCA	GTA	TGA	GTC	TTT	TCC	TCG	GTG	GCT	TAG	TCC	TGT	TGG
To	GAA	TAC	TAA	GAT	TTG	CAT	TCA	TGC	GTT	GCA	GTA	TGA	GTC	TTT	TCC	TCG	GTG	GCT	TAG	TCC	TGT	TGG
Tj	GAA	TAC	TAA	GAT	TTG	CAT	TCA	TGC	GTT	GCA	GTA	TGA	GTC	TTT	TCC	TCG	GTG	GCT	TAG	TCC	TGT	TGG
Tt	GAA	TAC	TAA	GAT	TTG	CAT	TCA	TGC	GTT	GCA	GTA	TGA	GTC	TTT	TCC	TCG	GTG	GCT	TAG	TCC	TGT	TGG
Uh	GAA	TAC	TAA	GAT	TTG	CAT	TCA	TGC	GTT	GCA	GTA	TGA	GTC	TTT	TCC	TCG	GTG	GCT	TAG	TCC	TGT	TGG
Us	GAA	TAC	TAA	GAT	TTG	CAT	TCA	TGC	GTT	GCA	GTA	TGA	GTC	TTT	TCC	TCG	GTG	GCT	TAG	TCC	TGT	TGG

Table S8. Occurrence of nucleotide pair types in 25 pair sites common to 22 tRNAs in the mitochondrial genome of 37 Carangidae species

tRNA Phe																						
		Base pairing in AA stem								Base pairing in DHU stem				Base pairing in AC stem					Base pairing in TΨU stem			
		1-87	2-86	3-85	4-84	5-83	6-82	7-81	8-80	11-33	12-32	13-31	14-30	35-53	36-52	37-51	38-50	39-49	59-79	60-78	61-77	62-76
Watson and crick base pairs	T-A	2			35	3		1				36	25	37							4	
	A-T						4								37	32		37				
	G-C		37				23		37	32						5	7				1	37
	C-G			37		1		34			36						30		37	37	27	
Wobble base pair	T-G				2	2		2			1										1	
	G-T						4			5												
Others	A-A					30																
	A-C						1														4	
	A-G					1																
	C-A											1										
	C-C						5															
	C-T																					
	G-A																					
	GG																					
	T-C																					
	T-T																					
Total		2	37	37	37	37	37	37	37	37	37	37	25	37	37	37	37	37	37	37	37	37
Pairing (%)		100	100	100	100	21.62	82.35	100	100	100	100	97.29	100	100	100	100	100	100	100	100	89.18	100
Non-Pairing (%)		0	0	0	0	83.78	17.64	0	0	0	0	2.70	0	0	0	0	0	0	0	0	10.81	0

Table S8. cont.

tRNA Val																							
		Base pairing in AA stem								Base pairing in DHU stem				Base pairing in AC stem					Base pairing in TΨU stem				
		1-87	2-86	3-85	4-84	5-83	6-82	7-81	8-80	11-33	12-32	13-31	14-30	35-53	36-52	37-51	38-50	39-49	59-79	60-78	61-77	62-76	63-75
Watson and crick base pairs	T-A	2						9				37	3	36		37			35				
	A-T			37			3						1							25	4		
	G-C				37	5	34		37	35										8	28	37	
	C-G		37					26			37				37		37		2				37
Wobble base pair	T-G							2															
	G-T									2										3			
Others	A-A																						
	A-C					32															5		
	A-G																						
	C-A													1									
	C-C																	7					
	C-T																	30					
	G-A																						
	GG																						
	T-C																						
	T-T																						
Total		2	37	37	37	37	37	37	37	37	37	37	4	37	37	37	37	37	37	37	37	37	37
Pairing (%)		100	100	100	100	13.51	100	100	100	100	100	100	100	97.05	100	100	100	0.00	100	100	86.48	100	100
Non Pairing (%)		0	0	0	0	86.48	0	0	0	0	0	0	0	2.94	0	0	0	100	0	0	14.70	0	0

Table S8. cont.

tRNA Leu (TAA)																						
		Base pairing in AA stem							Base pairing in DHU stem				Base pairing in AC stem					Base pairing in TΨU stem				
		1-87	2-86	3-85	4-84	5-83	6-82	7-81	11-33	12-32	13-31	14-30	35-53	36-52	37-51	38-50	39-49	59-79	60-78	61-77	62-76	63-75
Watson and crick base pairs	T-A		37	37														37		34		
	A-T				37		7				37		37	37	37							
	G-C	37				37		37	37							37	33					
	C-G									37									37		37	37
Wobble base pairs	T-G																			3		
	G-T											37										
Others	A-A																					
	A-C						25										4					
	A-G																					
	C-A																					
	C-C																					
	C-T																					
	G-A																					
	GG																					
	T-C						2															
	T-T						3															
Total		37	37	37	37	37	37	37	37	37	37	37	37	37	37	37	37	37	37	37	37	37
Pairing (%)		100	100	100	100	100	18.91	100	100	100	100	100	100	100	100	100	89.18	100	100	100	100	100
Non Pairing (%)		0	0	0	0	0	81.08	0	0	0	0	0	0	0	0	0	10.81	0	0	0	0	0

Table S8. cont.

tRNA Ile																						
		Base pairing in AA stem								Base pairing in DHU stem			Base pairing in AC stem					Base pairing in TΨU stem				
		1-87	2-86	3-85	4-84	5-83	6-82	7-81	8-80	11-33	12-32	13-31	35-53	36-52	37-51	38-50	39-49	59-79	60-78	61-77	62-76	63-75
Watson and crick base pairs	T-A						1	31	5			37					37		3			
	A-T	26	3		34	2									37							
	G-C		37	37	3	35	3		25													
	C-G						1			37	37			37		37		37	37	37	37	37
Wobble base pairs	T-G						2	3														
	G-T	1																				
Others	A-A																					
	A-C																					
	A-G																					
	C-A						30	3					37									
	C-C																					
	C-T																					
	G-A																					
	GG																					
	T-C																					
	T-T																					
Total		27	37	37	37	37	37	37	30	37	37	37	37	37	37	37	37	37	37	37	37	37
Pairing (%)		100	100	100	100	100	18.91	91.89	100	100	100	100	0.00	100	100	100	100	100	100	100	100	100
Non Pairing (%)		0	0	0	0	0	81.08	8.10	0	0	0	0	100	0	0	0	0	0	0	0	0	0

Table S8. cont.

tRNA Gln																							
		Base pairing in AA stem								Base pairing in DHU stem				Base pairing in AC stem					Base pairing in TΨU stem				
		1-87	2-86	3-85	4-84	5-83	6-82	7-81	8-80	11-33	12-32	13-31	14-30	35-53	36-52	37-51	38-50	39-49	59-79	60-78	61-77	62-76	63-75
Watson and crick base pairs	T-A	5	37								37									32			
	A-T			37			37	37						37	37		37						
	G-C				37				37	36		37				36		37					
	C-G																			5	37	37	37
Wobble base pairs	T-G												37										
	G-T					37																	
Others	A-A																						
	A-C									1													
	A-G																						
	C-A																						
	C-C																						
	C-T																						
	G-A																						
	GG																						
	T-C															1							
	T-T																		37				
Total		5	37	37	37	37	37	37	37	37	37	37	37	37	37	37	37	37	37	37	37	37	37
Pairing (%)		100	100	100	100	100	100	100	100	97.27	100	100	100	100	100	97.27	100	100	0.00	100	100	100	100
Non Pairing (%)		0	0	0	0	0	0	0	0	2.70	0	0	0	0	0	2.70	0	0	100	0	0	0	0

Table S8. cont.

tRNA Met																							
		Base pairing in AA stem								Base pairing in DHU stem				Base pairing in AC stem					Base pairing in TΨU stem				
		1-87	2-86	3-85	4-84	5-83	6-82	7-81	8-80	11-33	12-32	13-31	14-30	35-53	36-52	37-51	38-50	39-49	59-79	60-78	61-77	62-76	63-75
Watson and crick base pairs	T-A	35			3								32	37	37						37		
	A-T		37	5		37		6		37													
	G-C			32			29	1	37		37					37	37	37		5			
	C-G				27							37							36			37	37
Wobble base pairs	T-G				7																		
	G-T						1	25												11			
Others	A-A																						
	A-C						4																
	A-G						3																
	C-A																						
	C-C																						
	C-T																			1			
	G-A																						
	GG																						
	T-C							5															
	T-T																			20			
Total		35	37	37	37	37	37	37	37	37	37	37	32	37	37	37	37	37	36	37	37	37	37
Pairing (%)		100	100	100	100	100	81.08	86.8	100	100	100	100	100	100	100	100	100	100	100	43.24	100	100	100
Non Pairing (%)		0	0	0	0	0	18.91	13.51	0	0	0	0	0	0	0	0	0	0	0	56.75	0	0	0

Table S8. cont.

tRNA Trp																						
		Base pairing in AA stem							Base pairing in DHU stem				Base pairing in AC stem					Base pairing in TΨU stem				
		1-87	2-86	3-85	4-84	5-83	6-82	7-81	11-33	12-32	13-31	14-30	35-53	36-52	37-51	38-50	39-49	59-79	60-78	61-77	62-76	63-75
Watson and crick base pairs	T-A							37			6	2							1		37	
	A-T	37		8		36							37		7							
	G-C		37	12	37	1			37	37				36	21	36						
	C-G						35										37	37	29	37		37
Wobble base pairs	T-G											35										
	G-T						2															
Others	A-A										31											
	A-C			17										1	9	1						
	A-G																					
	C-A																		7			
	C-C																					
	C-T																					
	G-A																					
	GG																					
	T-C																					
	T-T																					
Total		37	37	37	37	37	37	37	37	37	37	37	37	37	37	37	37	37	37	37	37	37
Pairing (%)		100	100	54.05	100	100	100	100	100	100	16.21	100	100	97.29	75.67	97.29	100	100	78.37	100	100	100
Non Pairing (%)		0	0	45.94	0	0	0	0	0	0	83.78	0	0	2.70	24.32	2.70	0	0	18.91	0	0	0

Table S8. cont.

tRNA Ala																							
		Base pairing in AA stem								Base pairing in DHU stem				Base pairing in AC stem					Base pairing in TΨU stem				
		1-87	2-86	3-85	4-84	5-83	6-82	7-81	8-80	11-33	12-32	13-31	14-30	35-53	36-52	37-51	38-50	39-49	59-79	60-78	61-77	62-76	63-75
Watson and crick base pairs	T-A	20						35	37			37	37	9		37		10					
	A-T		32	37																			
	G-C				37			2		3							37						
	C-G						37				37			9	33				37		37	37	37
Wobble base pairs	T-G													10	4			27					
	G-T		5			37				30										37			
Others	A-A																						
	A-C																						
	A-G																						
	C-A																						
	C-C																						
	C-T																						
	G-A																						
	GG																						
	T-C																						
	T-T									4				9									
Total		20	37	37	37	37	37	37	37	37	37	37	37	37	37	37	37	37	37	37	37	37	37
Pairing (%)		100	100	100	100	100	100	100	100	89.18	100	100	100	75.67	100	100	100	100	100	100	100	100	100
Non Pairing (%)		0	0	0	0	0	0	0	0	10.81	0	0	0	24.32	0	0	0	0	0	0	0	0	0

Table S8. cont.

tRNA Asn																							
		Base pairing in AA stem							Base pairing in DHU stem					Base pairing in AC stem					Base pairing in TΨU stem				
		1-87	2-86	3-85	4-84	5-83	6-82	7-81	11-33	12-32	13-31	14-30	15-29	35-53	36-52	37-51	38-50	39-49	59-79	60-78	61-77	62-76	63-75
Watson and crick base pairs	T-A	37				15					14			5	37	37					37		
	A-T		37		37		22										37			5			
	G-C			37			5	37	33									37		14			
	C-G					8				37		37		32					37			37	37
Wobble base pairs	T-G					14					23												
	G-T						10						14							18			
Others	A-A																						
	A-C																						
	A-G																						
	C-A																						
	C-C																						
	C-T																						
	G-A																						
	GG																						
	T-C																						
	T-T																						
Total		37	37	37	37	37	37	37	33	37	37	37	14	37	37	37	37	37	37	37	37	37	37
Pairing (%)		100	100	100	100	100	100	100	100	100	100	100	100	100	100	100	100	100	100	100	100	100	100
Non Pairing (%)		0	0	0	0	0	0	0	0	0	0	0	0	0	0	0	0	0	0	0	0	0	0

Table S8. cont.

tRNA Cys																							
		Base pairing in AA stem								Base pairing in DHU stem				Base pairing in AC stem					Base pairing in TΨU stem				
		1-87	2-86	3-85	4-84	5-83	6-82	7-81	8-80	11-33	12-32	13-31	14-30	35-53	36-52	37-51	38-50	39-49	59-79	60-78	61-77	62-76	63-75
Watson and crick base pairs	T-A				5						37		32	32							35		1
	A-T	37	37												26	31		32					
	G-C			37					37	3		37				6	37			37			
	C-G				32	6	33	20					3	5	9				37			36	36
Wobble base pairs	T-G					31	4	17														1	
	G-T									37					1								
Others	A-A																						
	A-C																	5					
	A-G																						
	C-A																				1		
	C-C																						
	C-T																						
	G-A																						
	GG																						
	T-C																						
	T-T														1								
Total		37	37	37	37	37	37	37	37	37	37	37	35	37	37	37	37	37	37	37	37	37	37
Pairing (%)		100	100	100	100	100	100	100	100	100	100	100	100	100	97.29	100	100	97.29	100	100	97.29	100	100
Non Pairing (%)		0	0	0	0	0	0	0	0	0	0	0	0	0	2.70	0	0	13.51	0	0	2.70	0	0

Table S8. cont.

tRNA Tyr																						
		Base pairing in AA stem							Base pairing in DHU stem				Base pairing in AC stem					Base pairing in TΨU stem				
		1-87	2-86	3-85	4-84	5-83	6-82	7-81	11-33	12-32	13-31	14-30	35-53	36-52	37-51	38-50	39-49	59-79	60-78	61-77	62-76	63-75
Watson and crick base pairs	T-A			36							37			37				37		37		
	A-T				37	4		37														
	G-C	37	36			1	34		37						37	37						
	C-G									37									37		37	37
Wobble base pairs	T-G			1																		
	G-T		1			32	3						37									
Others	A-A																					
	A-C																37					
	A-G																					
	C-A																					
	C-C																					
	C-T																					
	G-A											37										
	GG																					
	T-C																					
	T-T																					
Total		37	37	37	37	37	37	37	37	37	37	37	37	37	37	37	37	37	37	37	37	37
Pairing (%)		100	100	100	100	100	100	100	100	100	100	0	100	100	100	100	0.00	100	100	100	100	100
Non Pairing (%)		0	0	0	0	0	0	0	0	0	0	100	0	0	0	0	100	0	0	0	0	0

Table S8. cont.

tRNA Ser (TGA)																				
		Base pairing in AA stem						Base pairing in DHU stem			Base pairing in AC stem					Base pairing in TΨU stem				
		1-87	2-86	3-85	4-84	5-83	6-82	11-33	12-32	13-31	35-53	36-52	37-51	38-50	39-49	59-79	60-78	61-77	62-76	63-75
Watson and crick base pairs	T-A											37	37							
	A-T	37		37	37	34		31		37	3									
	G-C		37				37				5			37	37					
	C-G								37							37	37	3	37	37
Wobble base pairs	T-G																	34		
	G-T					3		6			29									
Others	A-A																			
	A-C																			
	A-G																			
	C-A																			
	C-C																			
	C-T																			
	G-A																			
	GG																			
	T-C																			
	T-T																			
Total		37	37	37	37	37	37	37	37	37	37	37	37	37	37	37	37	37	37	37
Pairing (%)		100	100	100	100	100	100	100	100	100	100	100	100	100	100	100	100	100	100	100
Non Pairing (%)		0	0	0	0	0	0	0	0	0	0	0	0	0	0	0	0	0	0	0

Table S8. cont.

tRNA Asp																							
		Base pairing in AA stem								Base pairing in DHU stem				Base pairing in AC stem					Base pairing in TΨU stem				
		1-87	2-86	3-85	4-84	5-83	6-82	7-81	8-80	11-33	12-32	13-31	14-30	35-53	36-52	37-51	38-50	39-49	59-79	60-78	61-77	62-76	63-75
Watson and crick base pairs	T-A	1					2				37			15	37							5	
	A-T		37	37		37		32				37	37										
	G-C				37			5		37						37				32			
	C-G						33		37					6			37	37	25		37	32	37
Wobble base pairs	T-G						2												12				
	G-T																			5			
Others	A-A													5									
	A-C																						
	A-G																						
	C-A													11									
	C-C																						
	C-T																						
	G-A																						
	GG																						
	T-C																						
	T-T																						
Total		1	37	37	37	37	37	37	37	37	37	37	37	37	37	37	37	37	37	37	37	37	37
Pairing (%)		100	100	100	100	100	100	100	100	100	100	100	100	56.75	100	100	100	100	100	100	100	100	100
Non Pairing (%)		0	0	0	0	0	0	0	0	0	0	0	0	43.24	0	0	0	0	0	0	0	0	0

Table S8. cont.

tRNA Lys																						
		Base pairing in AA stem								Base pairing in DHU stem				Base pairing in AC stem					Base pairing in TΨU stem			
		1-87	2-86	3-85	4-84	5-83	6-82	7-81	8-80	11-33	12-32	13-31	14-30	35-53	36-52	37-51	38-50	39-49	59-79	60-78	61-77	62-76
Watson and crick base pairs	T-A	4				37						37		37	37							
	A-T						15	37					1			37					37	
	G-C			37					37	37							37					
	C-G		37		37						37							37	37	37		37
Wobble base pairs	T-G																					
	G-T																					
Others	A-A																					
	A-C						22															
	A-G																					
	C-A																					
	C-C																					
	C-T																					
	G-A																					
	GG																					
	T-C																					
	T-T																					
Total		4	37	37	37	37	37	37	37	37	37	37	1	37	37	37	37	37	37	37	37	37
Pairing (%)		100	100	100	100	100	40.54	100	100	100	100	100	100	100	100	100	100	100	100	100	100	100
Non Pairing (%)		0	0	0	0	0	59.45	0	0	0	0	0	0	0	0	0	0	0	0	0	0	0

Table S8. cont.

tRNA Gly																						
		Base pairing in AA stem							Base pairing in DHU stem				Base pairing in AC stem					Base pairing in TΨU stem				
		1-87	2-86	3-85	4-84	5-83	6-82	7-81	11-33	12-32	13-31	14-30	35-53	36-52	37-51	38-50	39-49	59-79	60-78	61-77	62-76	63-75
Watson and crick base pairs	T-A		37		37	37	37			37					37							
	A-T	37							5		37		3				37		37	37		
	G-C													37		37		37				
	C-G			37				37				4									37	37
Wobble base pairs	T-G											33										
	G-T								32													
Others	A-A																					
	A-C												29									
	A-G																					
	C-A																					
	C-C												2									
	C-T																					
	G-A																					
	GG																					
	T-C												3									
	T-T																					
Total		37	37	37	37	37	37	37	37	37	37	37	37	37	37	37	37	37	37	37	37	37
Pairing (%)		100	100	100	100	100	100	100	100	100	100	100	8.10	100	100	100	100	100	100	100	100	100
Non Pairing (%)		0	0	0	0	0	0	0	0	0	0	0	91.89	0	0	0	0	0	0	0	0	0

Table S8. cont.

tRNA Arg																							
		Base pairing in AA stem								Base pairing in DHU stem				Base pairing in AC stem					Base pairing in TΨU stem				
		1-87	2-86	3-85	4-84	5-83	6-82	7-81	8-80	11-33	12-32	13-31	14-30	35-53	36-52	37-51	38-50	39-49	59-79	60-78	61-77	62-76	63-75
Watson and crick base pairs	T-A	6				22			37		37	30	37	25	35	37				10			
	A-T		37				9	31										37	33	3	23		
	G-C			37	36		3	3		31							37						
	C-G					7														20		37	37
Wobble base pairs	T-G					6									2					4			
	G-T						2			6											14		
Others	A-A																		1				
	A-C				1		10																
	A-G																						
	C-A											7		12									
	C-C					1																	
	C-T							1															
	G-A																						
	GG																						
	T-C						2																
	T-T					1	11	2															
Total		6	37	37	37	37	37	37	37	37	37	37	37	37	37	37	37	37	34	37	37	37	37
Pairing (%)		100	100	100	97.29	94.59	37.83	91.89	100	100	100	81.08	100	67.56	100	100	100	100	97.29	100	100	100	100
Non Pairing (%)		0	0	0	2.70	5.40	62.16	8.10	0	0	0	18.91	0	32.43	0	0	0	0	2.70	0	0	0	0

Table S8. cont.

tRNA His																						
		Base pairing in AA stem							Base pairing in DHU stem				Base pairing in AC stem					Base pairing in TΨU stem				
		1-87	2-86	3-85	4-84	5-83	6-82	7-81	11-33	12-32	13-31	14-30	35-53	36-52	37-51	38-50	39-49	59-79	60-78	61-77	62-76	63-75
Watson and crick base pairs	T-A		37				32			37	37	37	9	37				9		32		
	A-T			1	20	37		9							37		37					
	G-C	37		15	16			28	30							37						
	C-G						3						27					7	37	4	37	37
Wobble base pairs	T-G						2											21		1		
	G-T								7													
Others	A-A																					
	A-C			21	1																	
	A-G																					
	C-A												1									
	C-C																					
	C-T																					
	G-A																					
	GG																					
	T-C																					
	T-T																					
Total		37	37	37	37	37	37	37	37	37	37	37	37	37	37	37	37	37	37	37	37	37
Pairing (%)		100	100	43.24	97.29	100	100	100	100	100	100	100	97.29	100	100	100	100	100	100	100	100	100
Non Pairing (%)		0	0	56.75	2.70	0	0	0	0	0	0	0	2.70	0	0	0	0	0	0	0	0	0

Table S8. cont.

tRNA Ser (GCT)																					
		Base pairing in AA stem								Base pairing in AC stem						Base pairing in TΨU stem					
		1-87	2-86	3-85	4-84	5-83	6-82	7-81	8-80	35-53	36-52	37-51	38-50	39-49	40-48	59-79	60-78	61-77	62-76	63-75	64-74
Watson and crick base pairs	T-A																	3		1	
	A-T			37				37			5	33	33	16	30			28	33		
	G-C		37		37		37		37		22	3	3	14	7	36	37				
	C-G	34								30										32	37
Wobble base pairs	T-G																				
	G-T											1		4		1		6			
Others	A-A					37															
	A-C										7		1								
	A-G																				
	C-A																			4	
	C-C										1										
	C-T																		4		
	G-A																				
	GG																				
	T-C										1										
	T-T										1			3							
Total		34	37	37	37	37	37	37	37	30	37	37	37	37	37	37	37	37	37	37	37
Pairing (%)		100	100	100	100	0.00	100	100	100	100	72.97	100	97.05	91.89	100	100	100	100	89.18	89.18	100
Non Pairing (%)		0	0	0	0	100	0	0	0	0	27.02	0	2.94	8.10	0	0	0	0	10.81	10.81	0

Table S8. cont.

tRNA Leu (TAG)																						
		Base pairing in AA stem							Base pairing in DHU stem				Base pairing in AC stem					Base pairing in TΨU stem				
		1-87	2-86	3-85	4-84	5-83	6-82	7-81	11-33	12-32	13-31	14-30	35-53	36-52	37-51	38-50	39-49	59-79	60-78	61-77	62-76	63-75
Watson and crick base pairs	T-A			37			37					37	37	37			37					
	A-T							37			37								37	37		
	G-C	37							37	37					37	37		37				
	C-G		37			37															37	37
Wobble base pairs	T-G																					
	G-T																					
Others	A-A																					
	A-C																					
	A-G																					
	C-A																					
	C-C				33																	
	C-T				4																	
	G-A																					
	GG																					
	T-C																					
	T-T																					
Total		37	37	37	37	37	37	37	37	37	37	37	37	37	37	37	37	37	37	37	37	37
Pairing (%)		100	100	100	0.00	100	100	100	100	100	100	100	100	100	100	100	100	100	100	100	100	100
Non Pairing (%)		0	0	0	100	0	0	0	0	0	0	0	0	0	0	0	0	0	0	0	0	0

Table S8. cont.

tRNA Glu																							
		Base pairing in AA stem								Base pairing in DHU stem				Base pairing in AC stem					Base pairing in TΨU stem				
		1-87	2-86	3-85	4-84	5-83	6-82	7-81	8-80	11-33	12-32	13-31	14-30	35-53	36-52	37-51	38-50	39-49	59-79	60-78	61-77	62-76	63-75
Watson and crick base pairs	T-A			37	37	1		34			37	37			37								
	A-T	5	3						2					3									
	G-C		2						34	37			37			37	37		37	32			
	C-G					35	1	3													5	37	37
Wobble base pairs	T-G					1	36											37					
	G-T		32						1					34						5	4		
Others	A-A																						
	A-C																						
	A-G																						
	C-A																						
	C-C																						
	C-T																						
	G-A																						
	GG																						
	T-C																						
	T-T																				28		
Total		5	37	37	37	37	37	37	37	37	37	37	37	37	37	37	37	37	37	37	37	37	37
Pairing (%)		100	100	100	100	100	100	100	100	100	100	100	100	100	100	100	100	100	100	100	24.32	100	100
Non Pairing (%)		0	0	0	0	0	0	0	0	0	0	0	0	0	0	0	0	0	0	0	75.67	0	0

Table S8. cont.

tRNA Thr																						
		Base pairing in AA stem							Base pairing in DHU stem				Base pairing in AC stem					Base pairing in TΨU stem				
		1-87	2-86	3-85	4-84	5-83	6-82	7-81	11-33	12-32	13-31	14-30	35-53	36-52	37-51	38-50	39-49	59-79	60-78	61-77	62-76	63-75
Watson and crick base pairs	T-A					34					37		5				31		1	34		
	A-T			37			37															
	G-C	37						37	37						37	37						
	C-G		37							37		37	32	37				37	35	1	37	37
Wobble base pairs	T-G																6					
	G-T																					
Others	A-A																					
	A-C																					
	A-G																					
	C-A																		1	2		
	C-C				31																	
	C-T				3																	
	G-A																					
	GG																					
	T-C				2																	
	T-T				1	3																
Total		37	37	37	37	37	37	37	37	37	37	37	37	37	37	37	37	23	37	37	37	37
Pairing (%)		100	100	100	0.0	87	100	100	100	100	100	100	100	100	100	100	100	100	97.29	94.59	100	100
Non Pairing (%)		0	0	0	0	13	0	0	0	0	0	0	0	0	0	0	0	0	2.70	5.40	0	0

Table S8. cont.

tRNA Pro																							
		Base pairing in AA stem								Base pairing in DHU stem				Base pairing in AC stem					Base pairing in TΨU stem				
		1-87	2-86	3-85	4-84	5-83	6-82	7-81	8-80	11-33	12-32	13-31	14-30	35-53	36-52	37-51	38-50	39-49	59-79	60-78	61-77	62-76	63-75
Watson and crick base pairs	T-A	6									37	36			37				33			31	
	A-T			37		36	7	28	29							37							
	G-C				35		10		3								5						
	C-G		37											37				37	4	37	37	5	37
Wobble base pairs	T-G												37									1	
	G-T				2	1	18	9	5	37							32						
Others	A-A																						
	A-C																						
	A-G																						
	C-A																						
	C-C																						
	C-T																						
	G-A											1											
	GG																						
	T-C																						
	T-T						2																
Total		6	37	37	37	37	37	37	37	37	37	37	37	37	37	37	37	37	37	37	37	37	37
Pairing (%)		100	100	100	100	100	94.59	100	100	100	100	94.59	100	100	100	100	100	100	100	100	100	100	100
Non Pairing (%)		0	0	0	0	0	5.40	0	0	0	0	5.40	0	0	0	0	0	0	0	0	0	0	0

Table S9. Occurrence of termination associated sequences (TAS) in the control region of 37 species of Carangidae. *Dr= D. russelli, Ac= A. ciliaris, Ai= A. indica, Ad= A. djedaba, Ak= A. kleinii, Am= A. mate, Ca= C. armatus, Cb= C. bajad, Ce= C. equula, Cm= C. malabaricus, Cp= C. plagiotaenia, Ci= C. ignobilis, Ct= C. tille, Cmel=C. melampygus, Dm= D. macrosoma, Dmac = D. macerellus, Dmar= D. maruadsi, Dt= D. tabl, Eb= E. bipinnulata, Gs= G. speciosus, Mc= M. cordyla, Pn= P. niger, Pd= P. dentex, Tt= T. trachurus, Tj= T. japonicas, Sc=S. crumenophthalmus, Sd= S. dumerili, Sl=S. lalandi, Sq=S. quinqueradiata, Sle= S.leptolepis, Sr= Seriola rivoliana, Sn= S. nigrofasciata, Tb=T. blochii, To= T.ovatus, Tc=T. carolinus, Uh= U. helvola, Us= U. secunda*

Species	TACAT	ATGTA
Dr	5	4
Ac	3	3
Ai	5	6
Ad	3	5
Ak	3	3
Am	4	4
Ca	8	4
Cb	7	3
Ce	3	4
Cp	7	3
Cm	2	2
Ci	5	4
Ct	2	1
Cme	2	2
Dmac	6	3
Dm	5	4
Dmar	5	3
Dt	5	3
Eb	4	2
Gs	3	4
Mc	5	2
Pn	3	3
Pd	3	2
Tt	4	4
Tj	4	4
Sc	4	3
Sd	3	3
Sl	5	4
Sq	3	3
Sle	3	4
Sr	5	3
Sn	3	3
Tb	4	2
To	7	3
Tc	5	4
Us	3	3
Uh	3	6

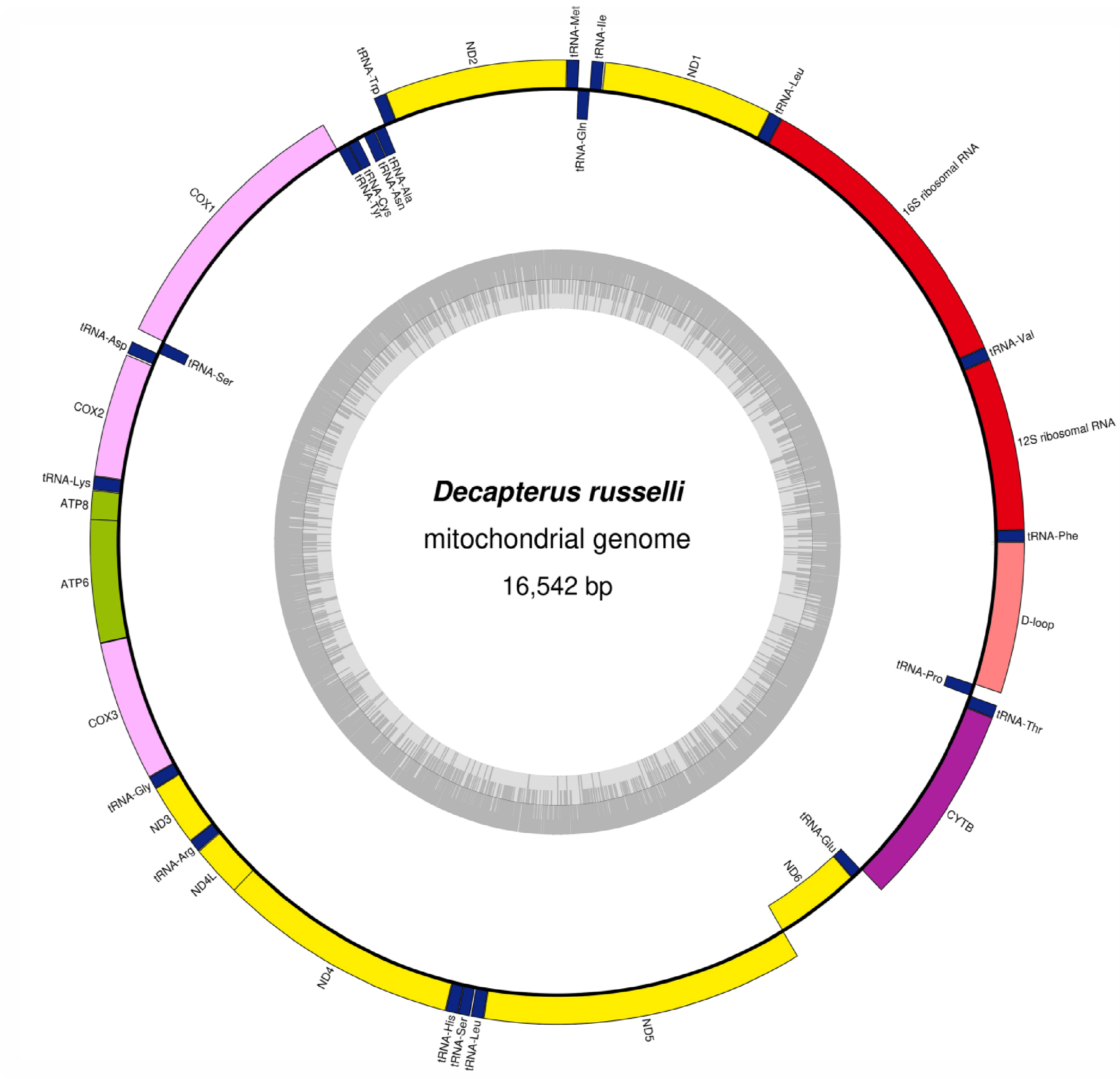

Figure S1. Map of the *Decapterus russelli* mitochondrial genome generated by OrganellarGenomeDRAW (https://chlorobox.mpimp-golm.mpg.de/OGDraw.html.)

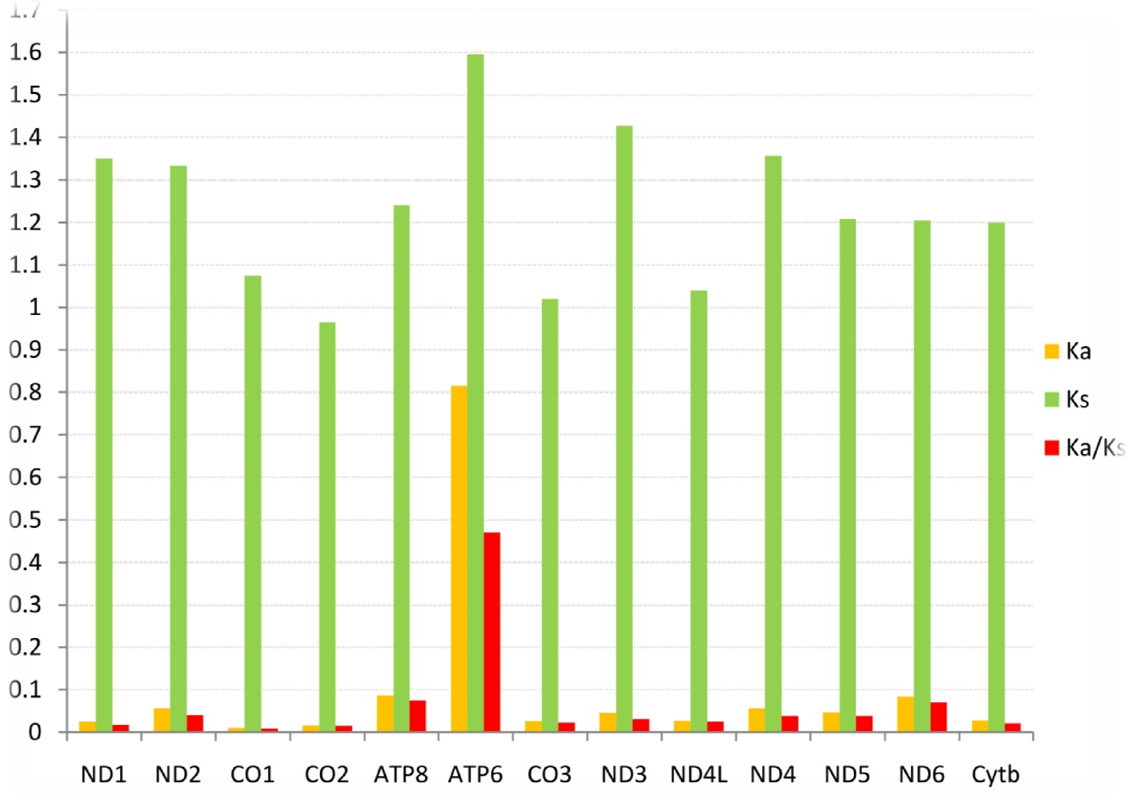

Figure S2. Evolutionary rate of *D. russelli* mitogenome. The rate of non-synonymous substitution, rate of synonymous substitution and the ratio of the rate of non-synonymous substitution to the rate of synonymous substitution for each PCGs.

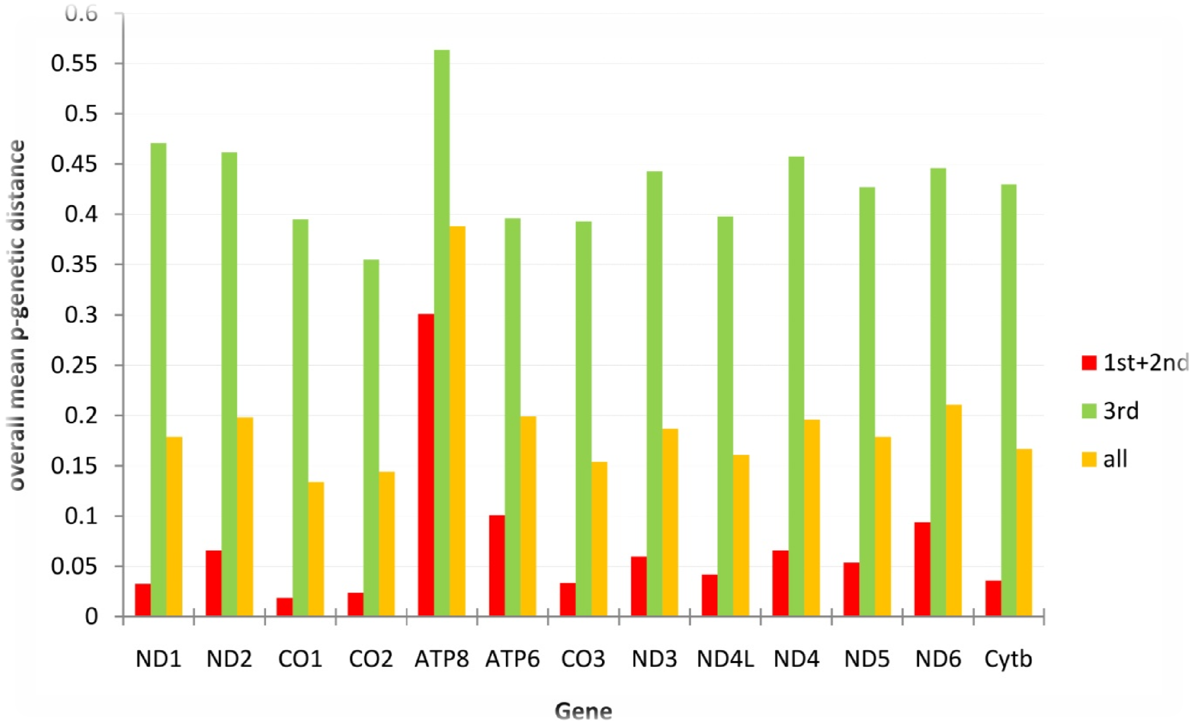

Figure S3. Overall mean p-genetic distance of 37 Carangidae species for each 13 protein coding genes. They were calculated based on the first and second nucleotide position, on the third nucleotide position of the amino acid codons, and on the full sequence of the protein coding genes respectively.

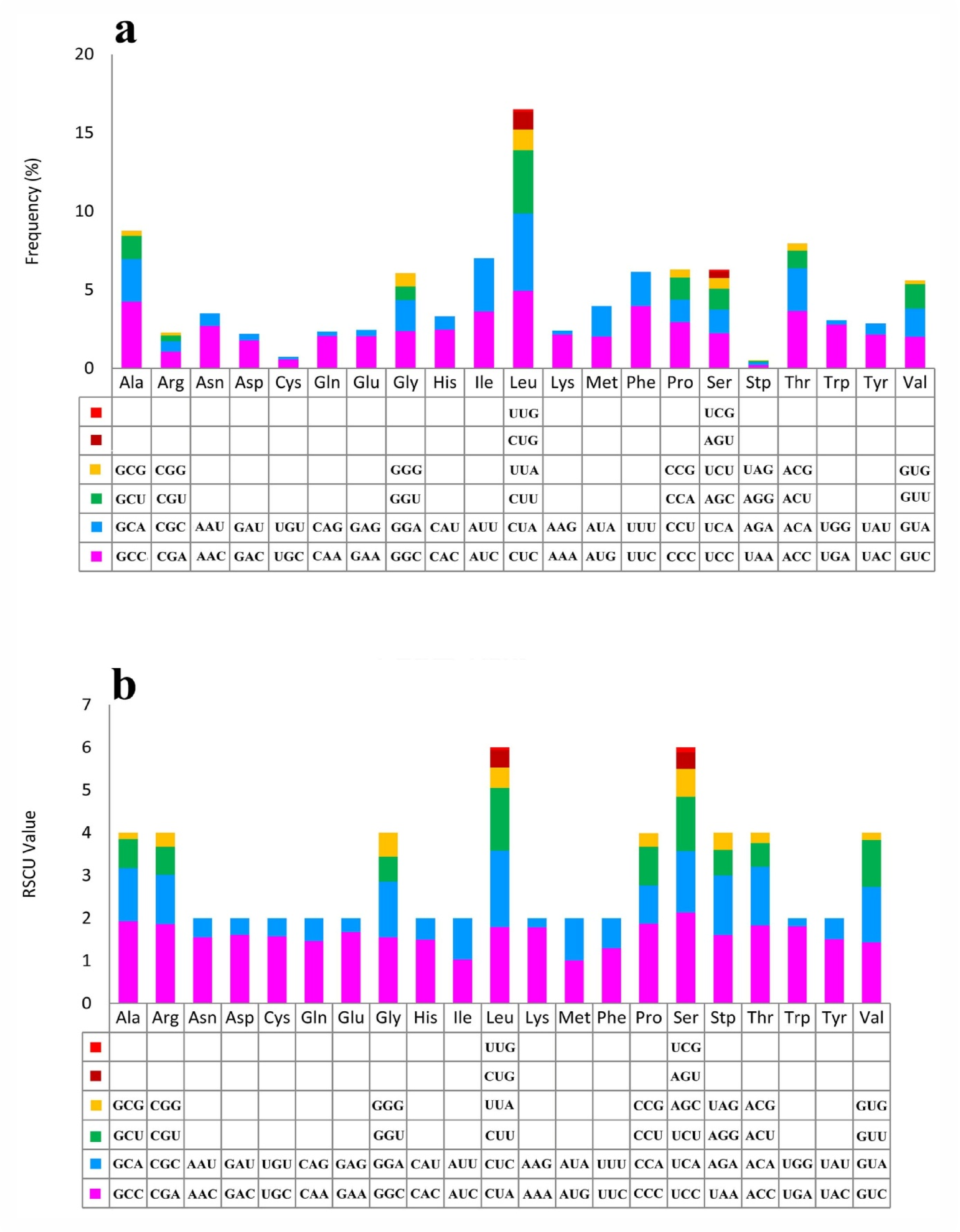

Figure S4. Codon frequency (**a**) RSCU (Relative Synonymous Codon Usage) (**b**) of the mitochondrial genome of *D. russelli.*

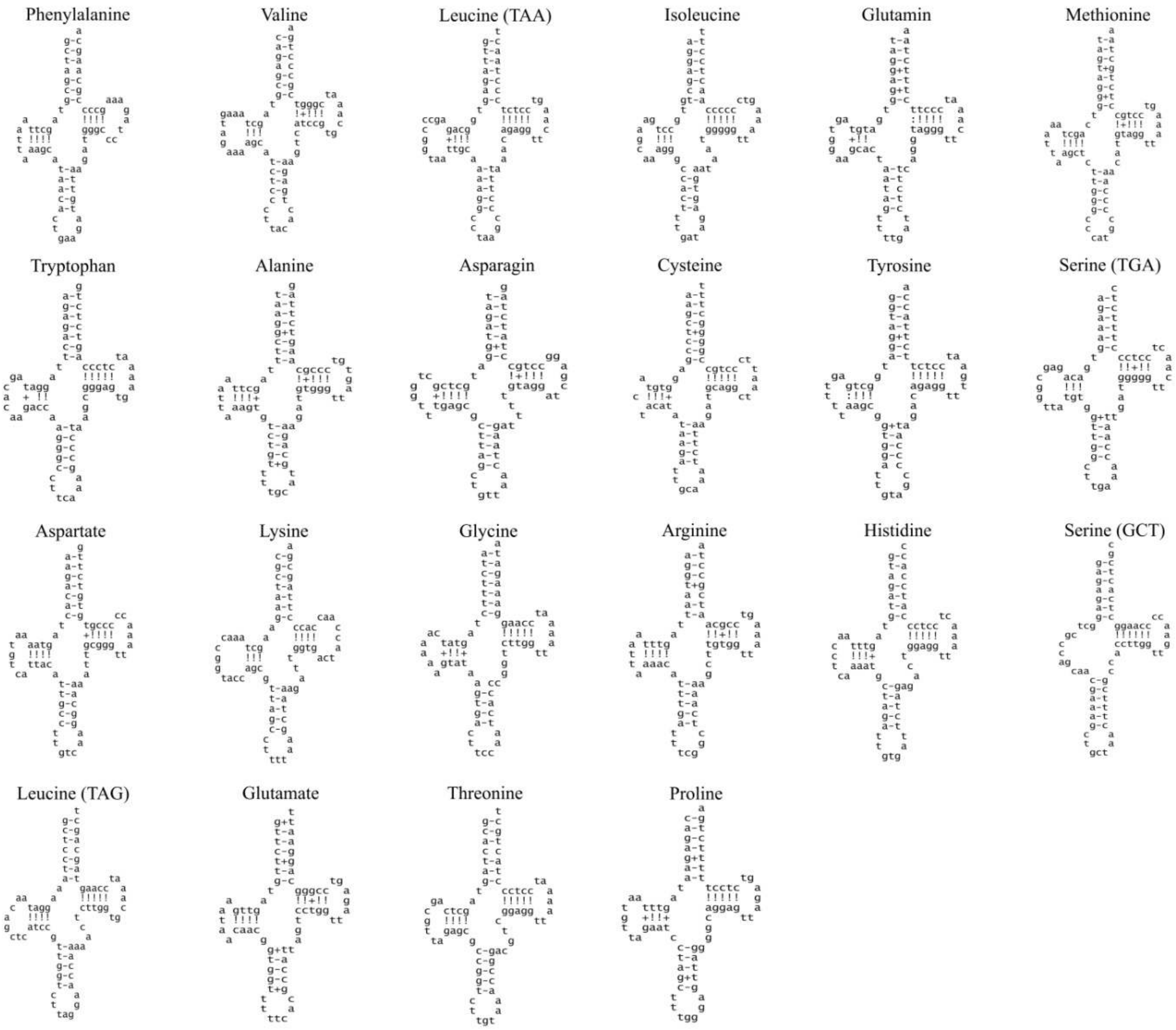

Figure S5. Putative secondary structures for 22 tRNA genes in the mitochondrial genome of *D. russelli.* Watson-crick and wobble base pairing (GT) are illustrated by – and + respectively. The secondary structure of tRNAs were predicted by ARWEN software (http://130.235.244.92/ARWEN/).

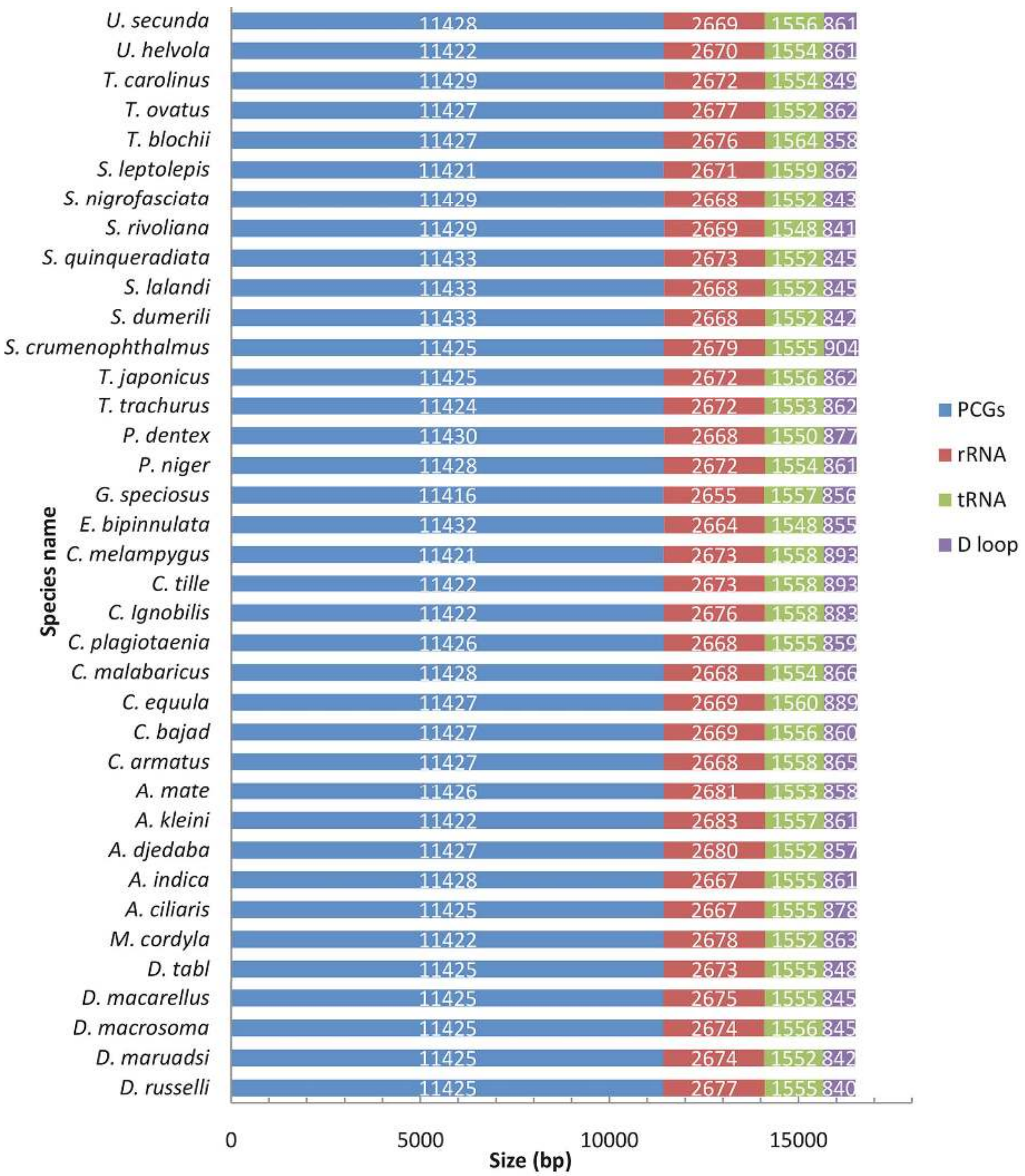

Figure S6. Variation in the length (bp) of PCGs, rRNA, tRNA and control region in the mitogenome of 37 species of Carangidae

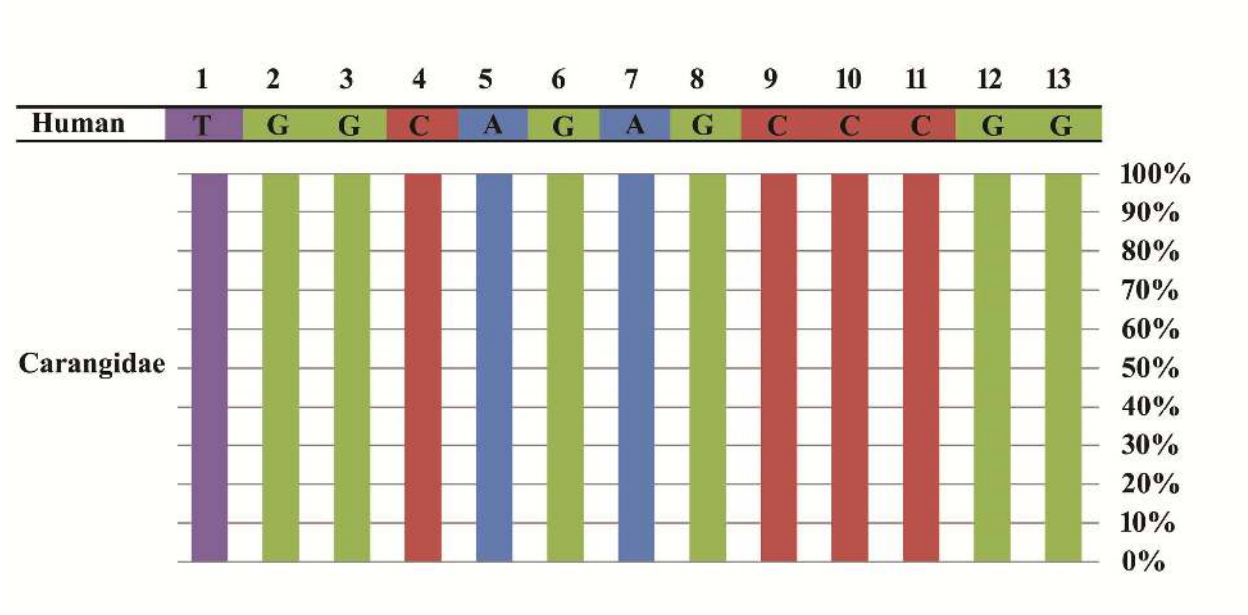

Figure S7. Base frequencies (%) of the mitochondrial transcription termination factor binding site in the tRNA-Leu (TAA) gene in the mitogenomes of 37 Carangids.

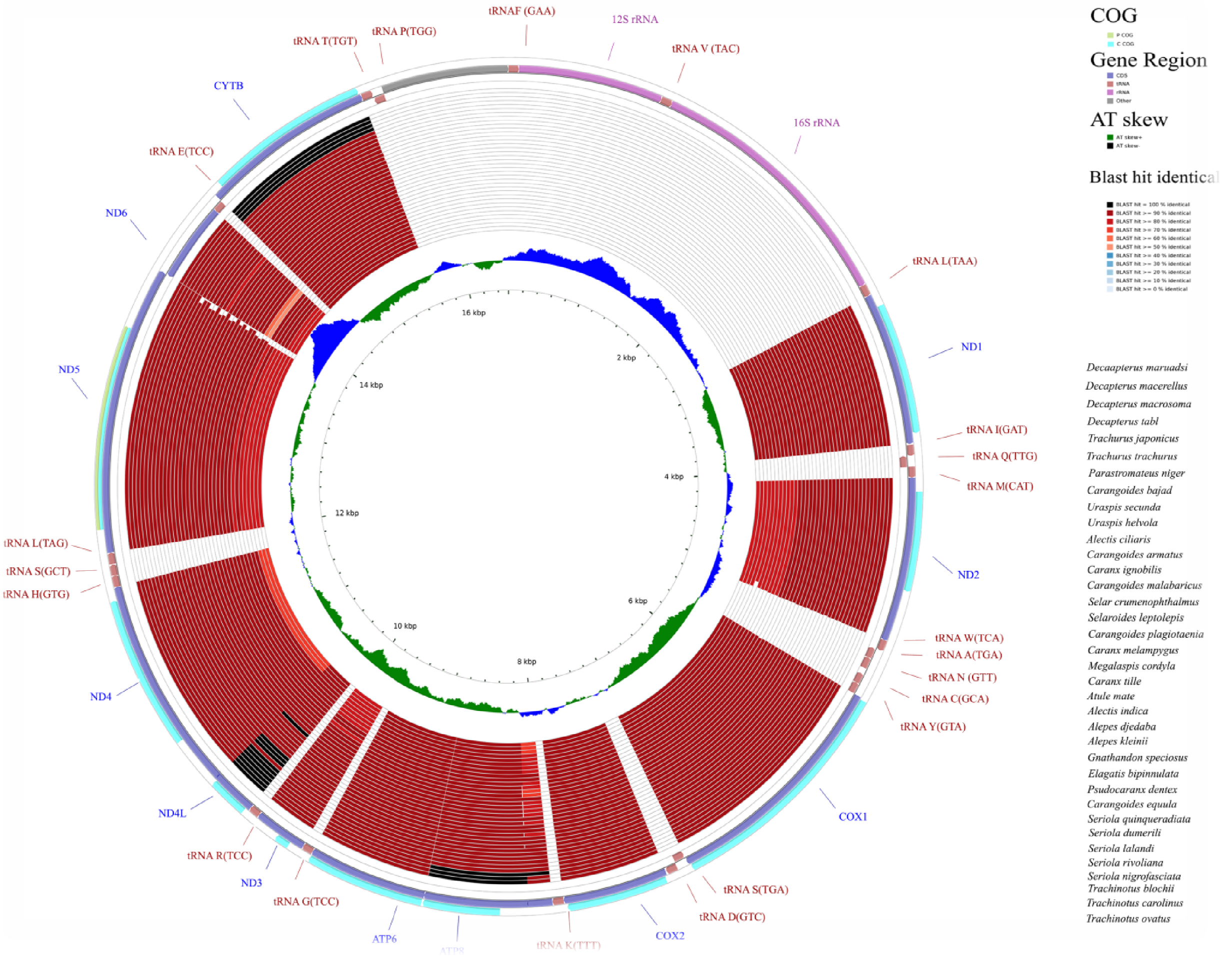

Figure S8. Graphical map of the blast results showing mitochondrial coding DNA sequence identity between *D. russelli* and 36 other Carangidae using CG view comparison tool (CCT) (https://github.com/paulstothard/cgview_comparison_tool). CCT arranges blast result in an order where sequence that most similar to the reference (*D. russelli*) is placed closer to the outer edge of the map. The clusters of orthologous groups of proteins (COGs), gene regions, AT skew, blast identity and species are shown from outside to inside.

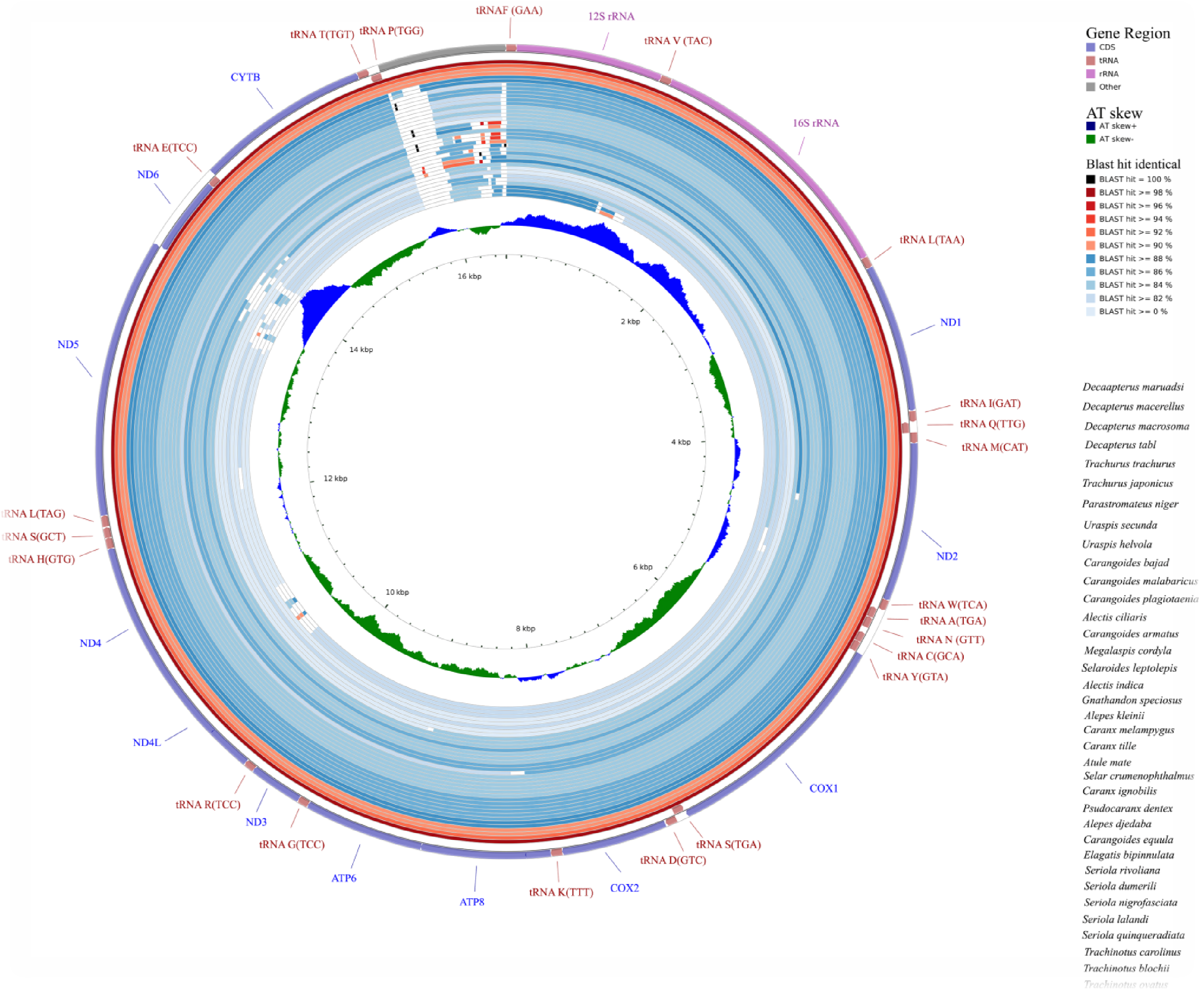

Figure S9. Graphical map of the blast results showing nucleotide identity between the complete mitogenome of *D. russelli* and 36 other Carangids using CG view comparison tool (CCT) (https://github.com/paulstothard/cgview_comparison_tool). CCT arranges blast result in an order where sequence that most similar to the reference (*D. russelli*) is placed closer to the outer edge of the map. The gene regions, AT skew, blast identity and species are shown from outside to inside.

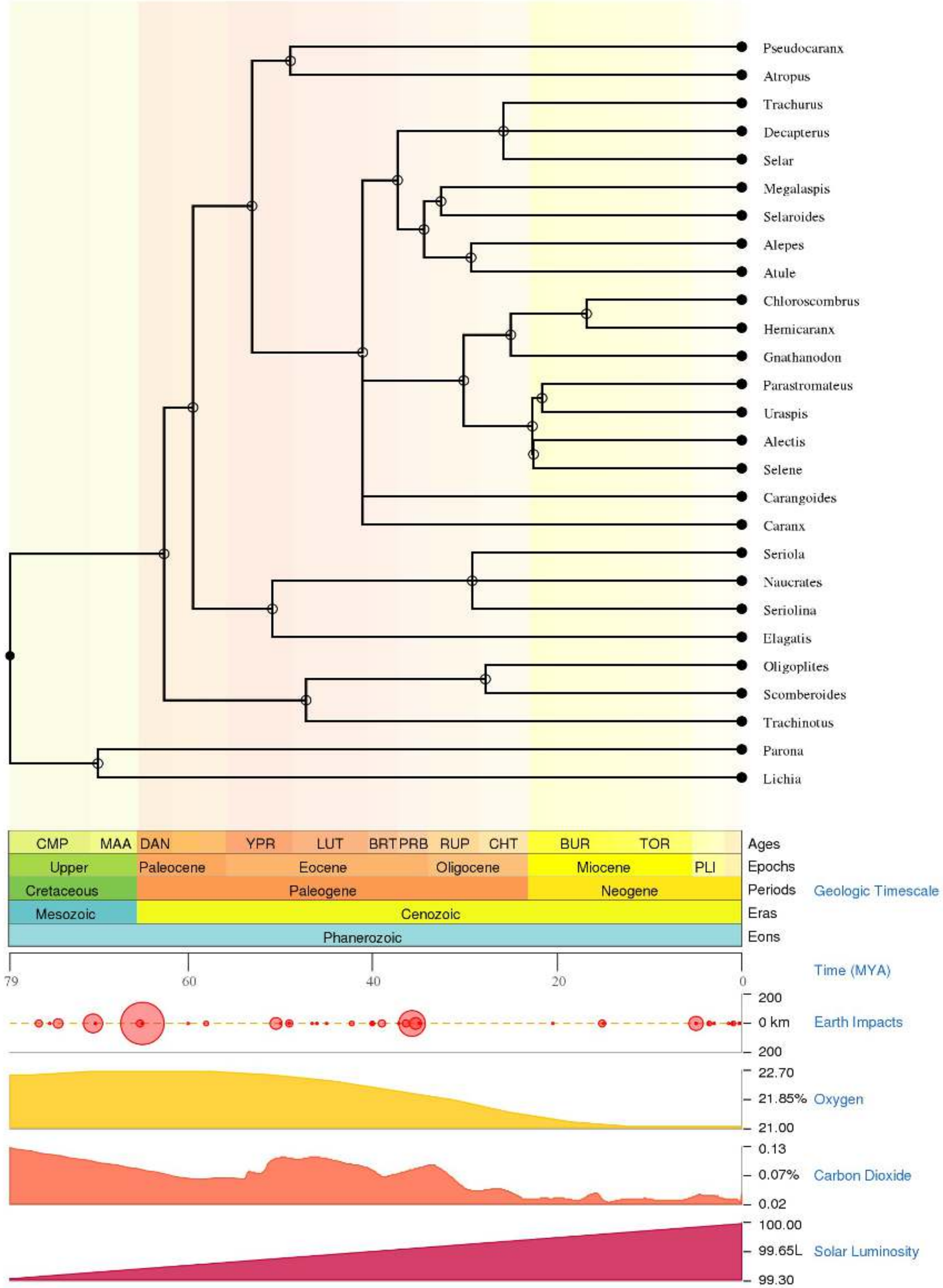

Figure S10. The divergence times of 27 genera of Carangidae generated by the Timetree database (http://www.timetree.org.). The changes in environmental parameters (Oxygen and Carbon dioxide level and solar luminosity) which affected the differentiation of the Carangidae are also shown.
